# The Effect of the Mediterranean Diet on Metabolic Health: A Systematic Review and Meta-Analysis of Controlled Trials in Adults

**DOI:** 10.3390/nu12113342

**Published:** 2020-10-30

**Authors:** Angeliki Papadaki, Eric Nolen-Doerr, Christos S. Mantzoros

**Affiliations:** 1 Centre for Exercise, Nutrition and Health Sciences, School for Policy Studies, University of Bristol, Bristol BS8 1TZ, UK; angeliki.papadaki@bristol.ac.uk; 2 Division of Endocrinology, Department of Medicine, Beth Israel Deaconess Medical Center, Harvard Medical School, Boston, MA 02215, USA; eric.nolen-doerr@bmc.org; 3 Department of Endocrinology, Boston VA Healthcare System, Boston, MA 02130, USA

**Keywords:** Mediterranean diet, metabolic syndrome, comorbidities, controlled trials, systematic review, meta-analysis

## Abstract

The Mediterranean diet (MD) may provide metabolic benefits but no systematic review to date has examined its effect on a multitude of outcomes related to metabolic health. This systematic review with meta-analysis (International Prospective Register of Systematic Reviews, PROSPERO; number CRD42019141459) aimed to examine the MD’s effect on metabolic syndrome (MetSyn) incidence, components and risk factors (primary outcomes), and incidence and/or mortality from MetSyn-related comorbidities and receipt of pharmacologic treatment for MetSyn components and comorbidities (secondary outcomes). We searched Pubmed, Embase, Cumulative Index to Nursing and Allied Health Literature (CINAHL), and Web of Science for controlled trials published until June 2019, comparing the MD with no treatment, usual care, or different diets in adults. Studies not published in English and not promoting the whole MD were excluded. Two authors independently extracted data and assessed risk of bias using the Cochrane Collaboration’s and Risk of Bias in non-randomised studies (ROBINS-I) tools. Reporting followed the Preferred Reporting Items for Systematic Reviews and Meta-Analyses (PRISMA) guidelines. Random-effects meta-analyses, subgroup analyses and meta-regressions were performed, and heterogeneity was quantified using the I^2^ statistic. We identified 2654 reports and included 84 articles reporting 57 trials (*n* = 36,983). In random effects meta-analyses, the MD resulted in greater beneficial changes in 18 of 28 MetSyn components and risk factors (body weight, body mass index, waist circumference, systolic and diastolic blood pressure, glucose, insulin, homeostatic model assessment of insulin resistance (HOMA-IR) index, total-, low-density lipoprotein (LDL)- and high-density lipoprotein (HDL)-cholesterol, triglycerides, alanine transaminase, hepatic fat mass, C-reactive protein, interleukin-6, tumour necrosis factor-a, and flow-mediated dilatation) and lower risk of cardiovascular disease incidence (risk ratio (RR) = 0.61, 95% confidence intervals (CI) 0.42–0.80; I^2^ = 0%), and stroke (RR = 0.67, 95% CI 0.35–0.98; I^2^ = 0%). Only six studies reported effects on pharmacotherapy use, and pooled analysis indicated no differences between diet groups. Lack of consistency in comparator groups and other study characteristics across studies resulted in high heterogeneity for some outcomes, which could not be considerably explained by meta-regressions. However, a consistent direction of beneficial effect of the MD was observed for the vast majority of outcomes examined. Findings support MD’s beneficial effect on all components and most risk factors of the MetSyn, in addition to cardiovascular disease and stroke incidence. More studies are needed to establish effects on other clinical outcomes and use of pharmacotherapy for MetSyn components and comorbidities. Despite the high levels of heterogeneity for some outcomes, this meta-analysis enabled the comparison of findings across studies and the examination of consistency of effects. The consistent direction of effect, suggesting the MD’s benefits on metabolic health, supports the need to promote this dietary pattern to adult populations.

## 1. Introduction

The prevalence of the metabolic syndrome (MetSyn) is increasing worldwide [1], with approximately one-quarter of the world’s population estimated to present with the syndrome [2]. MetSyn is associated with increased risk of comorbidities, including cardiovascular disease (CVD) [3], type 2 diabetes [4], non-alcoholic fatty liver disease (NAFLD) [5], and several forms of cancer [6]. Various therapies are routinely prescribed for some of these comorbidities, such as statins for primary [7], and coronary artery bypass grafting (CABG) or percutaneous coronary intervention (PCI) for secondary [8], prevention of CVD. Despite these therapies, dietary modifications continue to have an imperative role in MetSyn and MetSyn-related comorbidity prevention and management [9].

The Mediterranean diet (MD) is characterised by high intake of olive oil and plant foods (fruits, vegetables, legumes, nuts and non-refined cereals), low-to-moderate intake of dairy products, fish and poultry, moderate intake of alcohol, and low intake of red meat and sweets [10]. Two meta-analyses showed that the MD is associated with decreased risk of MetSyn [11] and produces favourable changes in MetSyn components [11,12]. These prior meta-analyses did not, however, report on other MetSyn risk factors, such as obesity markers and markers of inflammation, oxidative stress, insulin resistance, endothelial function and NAFLD, which would be important to establish the MD’s effect on all factors related to the MetSyn. The MD has also been linked to decreased risk of MetSyn-related comorbidities [13,14,15,16], but its effect on use of pharmacologic treatment for MetSyn components and comorbidities has not yet been examined. In addition, some earlier meta-analyses examining the effect of the MD on different health outcomes used questionable definitions of what constitutes this dietary pattern, or included studies where the dietary intervention only advised on the consumption of specific foods that are part of the MD [17,18], thus introducing biased conclusions. Overall, no study to date has systematically assessed and synthesised the evidence on the effect of the whole MD on MetSyn incidence and its components and risk factors, concurrently to MetSyn-related comorbidities and changes in use of pharmacotherapy for the treatment of MetSyn components and comorbidities. This is essential for establishing the MD’s effect on metabolic health [1].

To address this gap, and answer the research question ‘What is the effect of the MD on metabolic health in adults?’, we conducted a systematic review and meta-analysis of evidence from controlled trials of the effect of the MD, compared to no treatment, usual care, or different diets, on MetSyn incidence, MetSyn components (used in defining the MetSyn), and additional risk factors (primary objectives). Secondary objectives were incidence and/or mortality from MetSyn-related comorbidities and intermediate markers of these comorbidities, as well as changes in use of pharmacologic treatment for MetSyn components and comorbidities. This work updates and broadens the scope of earlier meta-analyses [11,12,13,14,15] and applies rigorous methodology to evaluate the available evidence.

## 2. Materials and Methods

The protocol for this systematic review and meta-analysis was registered with the International Prospective Register of Systematic Reviews (PROSPERO, number CRD42019141459). Reporting followed the Preferred Reporting Items for Systematic reviews and Meta-Analyses (PRISMA) guidelines (Supplementary Materials Table S1) [19].

### 2.1. Data Sources and Searches

The systematic search Supplementary Materials Table S2) included subject headings and keyword terms for the MD, MetSyn, and related comorbidities, and was conducted in PubMed, Embase, Web of Science Core Collection, and CINAHL (Cumulative Index to Nursing & Allied Health Literature), from inception until 17 June 2019. The search was limited to papers published in English. Reference lists of eligible papers were also hand-searched.

### 2.2. Study Selection

Studies were included if they were controlled trials (randomised and non-randomised) reporting pre- and post-intervention findings, and: (1) Participants were adults who were non-pregnant, non-lactating, and free of conditions that might affect their ability to eat certain foods; (2) the intervention promoted the whole MD or MD-style diet, with or without physical activity (as long as physical activity was equally promoted in the control group), and; (3) the MD was compared with no treatment, usual care, or advice to follow a different diet. Eligible studies focused on outcomes commonly assessed in everyday clinical practice in order to enhance relevance and translational potential to healthcare practitioners. Studies were included if they reported at least one of the following: (1) MetSyn incidence; (2) MetSyn components (waist circumference, blood pressure, and blood concentrations of high-density lipoprotein (HDL)-cholesterol, triglycerides, and glucose), and additional risk factors (anthropometric and biochemical markers, markers of oxidative stress, inflammation, endothelial function and insulin resistance); (3) incidence and/or mortality from MetSyn-related (type 2 diabetes, NAFLD, CVDs and cancer), and/or intermediate (e.g., pre-diabetes) comorbidities, and; (4) outcomes related to pharmacologic treatment for MetSyn components and the aforementioned comorbidities.

**Figure 1:**
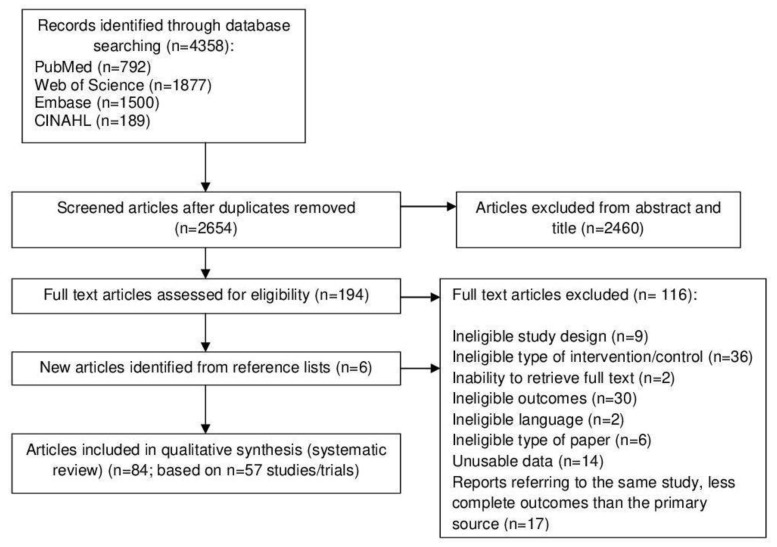
Flow diagram of literature search and study selection.

The titles and abstracts of identified papers were independently screened by two reviewers and the full text of all relevant papers was reviewed against the inclusion and exclusion criteria (Table 1; Supplementary Materials Table S3). Disagreements were resolved through discussion. Figure 1 illustrates the literature search and study selection process.

### 2.3. Data Extraction and Quality Assessment

Data extracted included study and population characteristics, and outcomes, including baseline, post-intervention, and follow-up values, as well as between-group changes in outcomes, where available. Studies were assessed as having low, high, or unclear risk of bias using the Cochrane risk of bias tool for randomised controlled trials [20]. For papers reporting on non-randomised controlled trials, the ROBINS-I (Risk of Bias in Non-randomised Studies of Interventions) tool was used to assess risk of bias [21]. Data were extracted and risk of bias was assessed independently by two reviewers into a piloted Excel form. Disagreements were resolved through discussion or third-party adjudication. Authors were contacted if clarification on any aspect of their reporting was required.

### 2.4. Data Synthesis and Analysis

For each included paper, effect sizes were summarised for each outcome by: (1) Calculating risk ratios (RRs) or hazard ratios (HRs) from the reported data (if these were not reported already), and; (2) calculating the mean difference between the intervention and control condition from the pre-intervention to the post-intervention period (and, where available, from the pre-intervention to the follow-up period). Data on outcomes were transformed, where applicable, into the same measurement units using standard conversion factors, to allow comparisons between studies, and a pooled analysis. If an included paper reported multiple outcomes of interest to the current review, each outcome was evaluated and reported independently. If an eligible and included paper reported on the follow-up measurements of an intervention study (for which the post-intervention results were reported in a separate, also included, paper), and both papers reported on the same outcome, findings from the follow-up were reported in the qualitative synthesis, but only the post-intervention findings were meta-analysed. If a paper was based on a study that had two comparison groups, we included the control condition where another diet was promoted (instead of usual care, no treatment, or usual diet).

As heterogeneity between studies was expected, a random-effects meta-analysis was used to summarise intervention effect estimates, expressed as RRs, HRs, or mean differences (inverse variance) with 95% confidence intervals (CIs). Heterogeneity between studies was assessed using the I^2^ statistic, with values >50% indicating substantial heterogeneity [22]. Papers reporting outcomes as median (range) or for which an appropriate combination of means, standard deviations, standard errors, or CIs for the outcomes of interest was not available (for example, to calculate the standard deviation of the difference between time points), were not included in the meta-analysis [23]. Where appropriate, a priori subgroup analyses were conducted to examine whether supplementing dietary advice on the MD with MD foods would result in greater effectiveness, compared to advice alone. This might have important translational implications, particularly in non-Mediterranean countries like the United States, where the MD is recommended within the dietary guidelines [24], but it is uncertain if Americans would find it easy to follow [25]. If the MD was suggested to be an effective strategy to improve MetSyn outcomes but barriers to using MD foods indeed obstructed dietary adherence, health systems might need to consider ‘prescribing’ MD foods to help adults adopt this diet for greater effects. However, there is no evidence to date to support this. Potential sources of heterogeneity across studies were further assessed using meta-regression, by comparing results from studies grouped according to study-level characteristics, including location (posthoc, Mediterranean vs. not), health status (posthoc, healthy vs. unhealthy at baseline), intervention duration (a priori, <6 m and ≥6 m, categories created based on data available), sample size (a priori, <150 and ≥150 participants, categories created based on data available), intervention diet (posthoc, MD vs. MD and another dietary component, e.g., a reduced-energy MD), and type of comparator (posthoc, no treatment; low-fat diet; reduced energy, low-fat diet; low-fat, high-carbohydrate diet; healthy diet or dietary guidelines, and; the National Cholesterol Education programme diet). Subgroup analyses were only conducted for outcomes with at least 10 studies included [26].

As the inclusion of non-randomised trials might introduce bias, particularly relating to confounding [21], a posthoc sensitivity analysis was conducted to examine the effect of excluding findings from these trials from the analysis. Further sensitivity analyses were conducted after exclusion of cross-over trials (a priori) and studies with ≥1000 participants (posthoc). Due to the low number of studies reporting on MetSyn incidence and the secondary outcomes, subgroup and sensitivity analyses were conducted only for MetSyn components and risk factors. STATA 13.0 (StataCorp LP, College Station, TX, USA) was used to conduct the meta-analyses and meta-regressions and to assess evidence of publication bias by visual evaluation of funnel plots and Egger tests (the statistical code is available from the authors upon request). Review Manager 5.3 was used to create risk of bias figures.

## 3. Results

Eighty-four papers (representing 57 unique trials) were included in the qualitative synthesis (Figure 1) [27,28,29,30,31,32,33,34,35,36,37,38,39,40,41,42,43,44,45,46,47,48,49,50,51,52,53,54,55,56,57,58,59,60,61,62,63,64,65,66,67,68,69,70,71,72,73,74,75,76,77,78,79,80,81,82,83,84,85,86,87,88,89,90,91,92,93,94,95,96,97,98,99,100,101,102,103,104,105,106,107,108,109,110,111], reporting on 36,983 participants (mean age 55.5 ± 11.5 years, range 21.2–71.0 years). The list of the 116 excluded papers can be found in Supplementary Materials Table S4.

### 3.1. Study Characteristics

Of the 84 papers (Supplementary Materials Table S5), 8 reported non-randomised controlled trials, 8 had a cross-over design, 43 were based in Mediterranean regions, and 49 on studies that supplemented advice with MD foods (Supplementary Materials Table S6). Sample sizes ranged from 11 [77] to 7447 [51,53]. Thirteen papers reported on studies that recruited healthy adults. Intervention duration varied from 10 days [65] to 7 years [78,79]. Thirty-six papers reported on studies where both the intervention and control groups received physical activity instructions. The dietary component of the intervention received by the intervention groups comprised of education on: The MD (*n* = 55); an energy-controlled MD (*n* = 1), a low-energy MD (*n* = 15), and a low-energy MD (if required) (*n* = 3); a low-energy, low-carbohydrate MD (*n* = 4); an Indo-MD (*n* = 2); and a low-glycaemic index MD (*n* = 2). The control groups received no treatment (*n* = 11); usual care (*n* = 1); and received advice to change their dietary habits in 72 papers (low-fat diet, *n* = 27; reduced energy low-fat diet, *n* = 10; low-fat, high-carbohydrate diet, *n* = 2; healthy eating, prudent diet, or general dietary guidelines, *n* = 13; usual Swedish diet, *n* = 1; usual German diet, *n* = 1; a north American diet, *n* = 2; reduced energy central European diet, *n* = 2; a high-fat or high-saturated fat diet, *n* = 2; a reduced energy and very low carbohydrate diet, *n* = 1; the National Cholesterol Education programme diet, *n* = 3; a low-glycemic index diet, *n* = 2; a vegan diet, *n* = 1; a reduced energy vegetarian diet, *n* = 1, and; a reduced energy prudent diet, *n* = 2) (Supplementary Materials Table S5).

Compliance to the intervention was assessed via conventional dietary assessment methods (e.g., food frequency questionnaires, 24-h recalls, food diaries, short adherence questionnaires, or a combination of these) in 53 papers; a combination of dietary assessment methods and biomarkers in 21 papers; compliance checklists in three papers; diet history interviews in two papers, and; biomarkers only in one paper. Three studies [72,73,102] did not report the method for assessing compliance while one study used supervision to assess compliance to treatment [83].

### 3.2. Metabolic Syndrome Incidence, Components and Risk Factors

Only one paper (reporting on two comparisons in 5801 participants) [31] examined the effect of the MD on MetSyn incidence risk; no between-group differences were found when the MD was supplemented with olive oil (control vs. MD: HR 1.10; 95% CI, 0.94–1.30), nor when it was supplemented with nuts (control vs. MD: 1.08; 0.92–1.27), compared to a low-fat control diet. The qualitative synthesis findings on MetSyn risk factors (for papers and/or outcomes not included in the pooled analysis) are presented in Supplementary Materials Tables S7–S9. The pooled analysis (Table 2 and Supplementary Materials Figures S1–S28) showed that the MD had a greater beneficial effect, compared to a control condition, on 18 of 28 MetSyn risk factors (body weight, body mass index, waist circumference, systolic and diastolic blood pressure, glucose, insulin, homeostatic model assessment of insulin resistance (HOMA-IR) index, total-, low-density lipoprotein (LDL)- and HDL-cholesterol, triglycerides, alanine transaminase, hepatic fat mass, C-reactive protein, interleukin-6, tumor necrosis factor-a, and flow-mediated dilatation), including all MetSyn components. There was substantial between-study heterogeneity for most of these meta-analyses, with null heterogeneity found only in four meta-analyses involving very few studies (Table 2).

### 3.3. Metabolic Syndrome-Related Comorbidities

The MD resulted in lower risk of CVD (RR 0.61; 95%CI, 0.42–0.80; I^2^ = 0%; two studies), and stroke incidence (0.67; 0.35–0.98; I^2^ = 0%; two studies), compared to a control condition. There were no between-group differences in CVD mortality (0.72; 0.43–1.01; I^2^ = 0%; three studies), sudden cardiac death (0.45; −0.15–1.04; I^2^ = 0%; two studies), heart failure incidence (0.69; 0.08–1.30; I^2^ = 59.4%; two studies), type 2 diabetes incidence (0.81; 0.61–1.02; I^2^ = 0%; two studies), and fatal (0.68; 0.23–1.12; I^2^ = 0%; two studies), and non-fatal MI (0.45; −0.001–0.900; I^2^ = 0%; two studies), despite effect estimates in the expected direction (Table 3 and Supplementary Materials Figures S29–S36). Findings from individual studies not included in the pooled analysis suggested a beneficial effect of the MD in reducing the risk of the incidence of angina (RR 0.60; 0.40–0.91; 1000 participants), pre-diabetes (0.19; 0.10–0.35; 406 participants), and breast cancer (HR 0.49; 95% CI, 0.25–0.94; 4152 participants), but not stroke mortality (RR 0.67; 0.11–4.02; 1000 participants), MI incidence (HR 0.80; 0.53–1.21; 7447 participants), or overall cancer incidence (RR 1.00;0.14–7.10; 1000 participants) or mortality (RR 1.00; 0.06–16.01; 1000 participants) (Supplementary Materials Table S9).

### 3.4. Pharmacotherapy

Six studies reported on the effect of the MD on pharmacologic treatment of MetSyn components and/or related comorbidities [47,48,53,62,68,91,93]. In the pooled analysis (Table 3 and Supplementary Figures S37–S41), there were no differences between the MD and a control condition in the need for blood pressure medication (RR 0.99; 95% CI, 0.96–1.02; I^2^ = 0%; three studies), lipid-lowering agents (1.01; 0.95–1.08; I^2^ = 0%; two studies), anti-platelet therapy (0.99; 0.90–1.08; I^2^ = 0%; two studies), insulin (0.99; 0.78–1.20; I^2^ = 0%; two studies), or oral anti-diabetic agents (0.83;0.58–1.09; I^2^ = 64.2%; three studies). Evidence from one study in 1000 participants suggested a beneficial effect of the MD, compared to a control condition, on the use of nitrates (RR 0.67; 95% CI, 0.54–0.83), β-blockers (0.55; 0.37–0.80), and disopyramide (0.28; 0.13–0.60) [93]. In the one study reporting a beneficial effect of the MD on hyperglycaemic drug use (HR 0.63; 95% CI, 0.50–0.81) [47] in 215 participants, this effect was maintained at follow-up (0.68; 0.50–0.89) [48] (Supplementary Table S10).

### 3.5. Quality Assessment

Supplementary Materials Figure S42 summarises the included randomised controlled trials’ risk of bias assessment. Overall, there was a low risk of bias for selective outcome reporting and random sequence generation. Acceptable participant retention and/or reporting of attrition bias were considered to be achieved by 50% of papers. Risk of bias for blinding of participants and personnel, a challenging task in behaviour change interventions, was evaluated as high for the vast majority of papers. The majority of papers were also considered to have high risk of other bias, due to e.g., the self-reported nature of assessing intervention compliance. Approximately 50% of papers did not report whether allocation was concealed and >50% whether assessment of outcomes was blinded. Across the 72 papers, risk of bias was considered low, unclear, and high for 41%, 26%, and 33% of the domains, respectively.

Visual evaluation of the funnel plots (Supplementary Materials Figures S43–S83) defined some asymmetry in some outcomes, but the Egger tests suggested small study effects only for body mass index (bias, −5.49; standard error (SE), 1.14; *p* < 0.001), waist circumference (bias, −6.77; SE, 2.85; *p* = 0.026), systolic (bias, 1.82; SE, 0.83; *p* = 0.039) and diastolic blood pressure (bias, 2.00; SE, 0.73; *p* = 0.011), total cholesterol (bias, 4.86; SE, 1.32; *p* = 0.001), and triglyceride concentrations (bias, 2.51; SE, 0.65; *p* < 0.001). For other outcomes, Egger tests did not suggest publication bias. Based on the ROBINS-I tool, two non-randomised controlled trials were evaluated as having serious risk of bias and one as having critical risk of bias, whereas there was no information on which to base a judgment about risk of bias in remaining studies (Supplementary Materials Table S11).

### 3.6. Subgroup Analyses

The subgroup analyses based on meta-regression are presented in Supplementary Materials Table S12. There was a greater effect of the MD compared with control diets on body weight (−2.82 vs. −0.41 kg; *p* = 0.011; *p*-heterogeneity < 0.001) and body mass index (−0.70 vs. 0.11 kg/m^2^; *p* < 0.001; *p*-heterogeneity < 0.001) when interventions were not supplemented with foods, compared with the subgroups of studies that provided foods. There was also a greater effect of the MD on blood glucose when studies did not supplement foods (−5.81 vs. −0.20 mg/dL; *p* = 0.016; *p*-heterogeneity < 0.001), were conducted in Mediterranean, compared to non-Mediterranean, countries (−5.47 vs. −0.31 mg/dL; *p* = 0.025; *p*-heterogeneity < 0.001), and when intervention duration was ≥6 months, compared to <6 months (−6.97 vs. −0.18 mg/dL; *p* = 0.002; *p*-heterogeneity < 0.001). A greater reduction in body weight (−2.97 vs. −1.09 kg; *p* = 0.043; *p*-heterogeneity < 0.001) and body mass index (−0.63 vs. −0.19 kg/m^2^; *p* = 0.035; *p*-heterogeneity < 0.001) was also observed in studies where the MD was promoted alongside another dietary component, compared to alone. Food supplementation accounted for approximately 18%, 55%, 17%, and 19% of the heterogeneity observed for body weight, body mass index, diastolic blood pressure, and blood glucose, respectively, whereas intervention duration accounted for 27% and 19% of the variance in blood glucose and C-reactive protein concentrations, respectively. However, high levels of within-group heterogeneity were observed for most subgroups, suggesting these findings might be uncertain.

### 3.7. Sensitivity Analyses

Sensitivity analysis, by excluding the non-randomised controlled trials [35,80,85,86,87,101,102], was conducted for 18 MetSyn components and risk factors (Supplementary Materials Table S13). Findings and heterogeneity levels largely remained unchanged, but were attenuated for waist circumference (−0.94 cm; 95% CI, −2.08–0.19; I^2^ = 100%; 24 studies), HDL-cholesterol (0.93 mg/dL; −0.06–1.92; I^2^ = 98%; 31 studies), and alanine transaminase (−2.39 UI/L; −5.77–0.99; I^2^ = 96%; 7 studies), whereas reduction of glycosylated hemoglobin (HbA1c) was larger in the MD, compared to a control condition (−0.29%; −0.40–−0.18; I^2^ = 4%; 5 studies). In addition, results were largely similar when cross-over trials [29,35,65,77,88,97,108,109] were excluded, although this attenuated the between-group difference in insulin concentrations (−0.77 μU/mL; 95%CI, −1.71–0.17; I^2^ = 97%; 15 studies) and hepatic fat mass (−2.04%; −5.95–1.88; I^2^ = 86%; 2 studies). Results remained unchanged when studies with ≥1000 participants [31,51,62,93,103] were excluded from the analyses. Excluding non-randomised trials reduced the levels of heterogeneity for HbA1c from 78% to 4%, but otherwise heterogeneity remained substantial (Supplementary Materials Table S13).

## 4. Discussion

This systematic review of 84 papers and 57 controlled trials aimed to evaluate the effect of the MD, compared to usual care, no treatment, or a different diet, on MetSyn incidence, MetSyn components and risk factors, in addition to related comorbidity outcomes and treatment for these health outcomes. To our knowledge, this is the most comprehensive list of MetSyn-related outcomes systematically assessed. We moved beyond evaluating effects on the factors used to define the MetSyn, to examining additional risk factors, such as markers of obesity, oxidative stress, inflammation, endothelial function, insulin resistance, and NAFLD, allowing for a more comprehensive metabolic health assessment. We found no evidence that the MD leads to lower incidence of the MetSyn, but there were greater beneficial changes in the majority of the MetSyn factors examined, in addition to lower risk of CVD incidence, and incidence of stroke. The qualitative synthesis also suggested that the MD lowers the risk of angina, pre-diabetes, and breast cancer, and results in lower need for the use of hyperglycaemic drugs, nitrates, β-blockers, and disopyramide, albeit from a small number of studies. There was no evidence for an effect on other comorbidity endpoints or use of other medications. Nevertheless, effect estimates for most of these outcomes favoured the MD. However, findings from this work should be viewed in light of the small number of studies for some outcomes, the considerable bias identified within a proportion of the included studies, and the substantial between-study heterogeneity found for most pooled analyses.

To our knowledge, this is the first study to examine the effect of the MD on MetSyn incidence. Results from only one study in 5801 participants showed no evidence of reduction in incidence risk [31]. An earlier meta-analysis of two clinical trials showed a 20% reduced risk of MetSyn presence [11], but this referred to studies reporting MetSyn prevalence at post-intervention, rendering comparisons with our findings challenging. More studies are therefore needed to establish the MD’s effect on MetSyn incidence. We also found that the MD results in beneficial changes in all components defining the MetSyn, in addition to the majority of the additional risk factors assessed. There was no evidence of an effect for other risk factors, despite effect estimates favouring the MD over a control treatment. These findings are supported by earlier meta-analyses (in 4133 and 16,689 participants), which showed a beneficial effect of the MD in the components defining the MetSyn [11,12]. Comparable to our findings, another meta-analysis in 2300 participants showed that the MD results in greater beneficial changes in several markers of inflammation and endothelial function, including C-reactive protein, interleukin-6, and flow-mediated dilatation [112]. Our review extends knowledge from these meta-analyses, incorporating more recent studies in 36,983 participants and evaluating, for the first time, the effect of the MD on markers of oxidative stress and NAFLD, demonstrating greater beneficial changes of the MD, compared to a control condition, in alanine transaminase concentrations, and hepatic fat mass, albeit from a small number of studies. More studies are therefore needed to examine the effect of the MD on these biomarkers of metabolic health.

It is noteworthy that, consistent with an earlier meta-analysis of clinical trials in 3436 participants reporting a decrease in body weight of 1.75 kg (95% CI −2.86–−0.64) [113], our analysis of 12,751 participants showed a similar finding (−1.72 kg; −2.40–−1.05; I^2^ = 98.6%), although results were slightly attenuated when the MD was promoted alone (−1.09 kg; *p* = 0.016) vs. alongside other dietary components, such as energy restriction (−2.97 kg; *p* < 0.001). The impact that adhering to the MD would have on caloric intake and body weight, stemming from e.g., the MD’s recommendation to have olive oil with every meal and nuts every day [10], has been documented as an important barrier to adhering to this dietary pattern by both healthy adults [114], and adults at high risk of developing CVD [115]. Given absence of evidence suggesting the MD promotes obesity, health practitioners should therefore consider not abstaining from promoting the MD to their patients over concerns that it is ‘fattening’. Even though the MD is suggested to be transferable [116], whether non-Mediterranean populations could adhere to this dietary pattern remains to be established [25]. Qualitative studies in the United Kingdom have suggested that cultural differences, cost, lack of cooking skills, and time could hinder acceptability and adherence to the MD [114,115]. Similar qualitative research and feasibility studies are needed in other non-Mediterranean countries, such as the United States, a country where a Mediterranean-style diet is recommended as a USDA-approved food pattern by the 2015 US Dietary Guidelines [24]. This will help explore facilitators and barriers to the MD, as well as the perceived intervention characteristics that would be acceptable to the diverse US population, before embarking on large randomised controlled trials testing the diet’s effectiveness on metabolic health.

Concomitant to concerns about the type of support that Americans and other non-Mediterranean populations might need to adopt the MD, are issues regarding the potential need to provide MD foods for free to facilitate dietary adherence. Our findings showed, for the first time, that while supplementing dietary advice with foods from the MD led to beneficial changes in some MetSyn factors, subgroup analyses revealed there is no evidence it leads to greater health benefits beyond those obtained by advice alone. Indeed, studies not providing foods led to greater changes in body weight, body mass index, and blood glucose concentrations, compared to those supplementing MD advice with foods. It might have been that participants consumed supplemented foods as an addition to, instead of substituting them for other foods in their diet, which might have affected health outcomes. However, this is challenging to confirm due to the varying levels of reporting of compliance with the dietary prescriptions across the reviewed studies. Also, the types and amounts of foods provided in the reviewed studies varied widely, from providing some components of the MD (e.g., olive oil, nuts, legumes, fish) to the majority [29,73,83,88], or all meals [61,85,86]. It is not suggested that providing MD foods is void of benefit. In certain clinical situations, supplementation could be integral in a patient’s care plan formulated under shared processes. However, more studies are needed to delineate nuanced impacts of food supplementation in these unique populations and socioeconomic settings. Head-to-head trials addressing these questions are thus needed.

Our results are overall consistent with earlier meta-analyses showing that the MD reduces risk of CVD, stroke [117], and breast cancer [15] incidence, but not heart failure [117]. In contrast to other reviews, we found no evidence of an effect of the MD on CVD mortality [118], and type 2 diabetes [119], while we additionally demonstrated a reduction in angina and pre-diabetes risk, albeit from one study. Comparisons between the current and earlier pooled analyses are hindered by different search strategies, study inclusion and exclusion criteria, and differing MD definitions. Nevertheless, we reported a 39% and 33% reduction in CVD and stroke incidence risk, respectively. This is a higher reduction compared to that demonstrated by statins for the primary prevention of CVD (RR 0.75; 95% CI, 0.70–0.81), and stroke (RR 0.78; 95% CI 0.68–0.89) [7], and for the secondary prevention of CVD in patients with type 2 diabetes (RR 0.85; 95% CI 0.79–0.91) [120]. The level of risk reduction demonstrated by our findings is comparable to the reduction in stroke incidence risk exhibited by PCI, when compared to CABG (RR 0.64; 95% CI, 0.48–0.85) [8]. It has also been shown that the MD reduces major vascular event risk by 37% (RR 0.63; 95% CI, 0.53–0.75) [117], whereas PCI, compared to CABG, increases this risk by 36% (RR 1.36; 95% CI 1.16–1.60) [8]. Although these comparisons should be interpreted with caution, they suggest that the MD could be an adjunctive therapeutic means for the primary and secondary prevention of CVD. However, randomised controlled trials comparing the MD versus statins and/or secondary prevention treatments are needed to confirm this hypothesis.

To our knowledge, no other review has systematically evaluated the MD’s effect on the need for pharmacologic treatment for MetSyn components and related comorbidities. Only six studies reported on the changes of the need for treatments, and of these, only one reported treatment as a primary outcome, showing that the MD reduced the need for antihyperglycemic drug therapy at four years [47]; maintained at the six-year follow-up [48]. All other studies reported changes in the proportion of participants requiring treatment post-intervention as a secondary outcome, and, thus, may not have had sufficient statistical power to detect any effects. Nevertheless, the beneficial effects of the MD on the use of nitrates, β-blockers, and disopyramide, albeit from one study in 1000 participants [93], as well as the results by Esposito et al. in 215 participants [47,48], suggest that the MD might be a promising means of reducing the economic burden that pharmacotherapy poses to patients and national health systems globally. However, more well-designed studies, using treatment for MetSyn components and related comorbidities as a primary outcome, are needed to ascertain this.

### Strengths and Limitations

Strengths of the current review include the use of rigorous methodology, according to current guidelines of conduct and reporting [19,20], to systematically evaluate, for the first time, the effect of the MD on a wide range of outcomes implicated in metabolic health. In contrast to earlier meta-analyses [17,18], we evaluated studies promoting the whole MD. We included both randomised and non-randomised controlled trials to obtain an indication of all intervention effects; however, only eight of the 84 reports involved non-randomised designs, and results following the sensitivity analysis largely remained unchanged after the exclusion of these. This work also has limitations. Despite a comprehensive search strategy, we included papers published in English only and we cannot exclude the possibility that eligible studies might not have been included due to the search terms. There were few studies identified and/or events documented for some outcomes. This reflects the challenge of conducting nutrition trials with hard clinical endpoints, where participants need to be followed for many years for such events to be observed. As some of the outcomes of this review constituted secondary outcomes for some studies, these might not have been sufficiently powered to provide valid conclusions. Further, although methods of assessment of compliance to the dietary treatments were documented in the majority of included papers, actual reporting of compliance findings was limited for most studies, and not deemed sufficient to delineate which intervention components may have contributed to the observed effects. Further, although risk of bias across studies was similar to that reported in earlier meta-analyses of complex interventions [23], several sources of bias identified as having unclear or high risk may affect our findings’ interpretation.

A further limitation is that significant and substantial between-study heterogeneity was observed for most MetSyn components and risk factors, particularly for outcomes with a large number of studies included. We accounted for this by applying random-effects models to account for variations across studies, and further assessed sources of heterogeneity by conducting subgroup analyses and meta-regressions, when the number of studies per health outcome allowed [26]. This level of heterogeneity, despite being similar to earlier meta-analyses of complex MD interventions [12,113], was not considerably explained by the subgroup analyses and meta-regressions, which creates uncertainty as to what the true effect of the MD might be, and limits the interpretation and generalizabilityof the findings. Other participant, study, or intervention characteristics, such as recruitment methods, intervention intensity, and delivery methods or sociocultural contexts, might have contributed to the heterogeneity across studies, but these are challenging to discern from published papers. Despite these high levels of heterogeneity for some outcomes, we proceeded with the meta-analysis, as this allowed us to compare findings across studies and examine the consistency of observed effects, in addition to facilitating the interpretation of results, which would be challenging without the pooled findings. As such, even though our results should be interpreted with caution, the consistent direction of effect that was observed in the majority of outcomes points towards the benefits of the MD.

## 5. Conclusions

This review of controlled trials provides evidence that the MD beneficially affects several outcomes implicated in metabolic health, including MetSyn components and several metabolic risk factors, in addition to incidence of CVD and stroke. Nevertheless, pooled findings should be interpreted with caution due to the substantial between-study heterogeneity found for most analyses and the small number of studies for some outcomes. Trials with appropriate comparator groups and more homogeneous designs are needed to help confirm whether the MD results in greater health benefits if combined with other intervention components, such as energy restriction, or if foods are provided to participants alongside advice to follow the diet, over and above those acquired separately by MD advice. More studies are also needed in non-Mediterranean populations, such as the United States of America, Northern Europe, and Australasia, to establish the feasibility and acceptability of the MD in these regions. Finally, more high-quality and adequately powered trials are needed to provide robust evidence on the effect of the MD on MetSyn incidence, other related comorbidities, such as NAFLD, and use of pharmacotherapy for MetSyn components and comorbidities, and delineate the biological mechanisms responsible for any health benefits. This would help establish whether the MD should form part of dietary guidelines globally, be widely promoted by healthcare practitioners and adopted by the general population for metabolic health benefits. Nevertheless, given the high health and economic burden of the MetSyn, its comorbidities, and the resulting need for pharmacotherapy to treat these, in conjunction with the consistent direction of beneficial effects observed in the current report, practitioners should not abstain from recommendingthe MD as a whole, and its food components, in intakes illustrated in the updated Mediterranean Diet Pyramid [10], to their adult patients for a wide range of metabolic health benefits.

## Figures and Tables

**Table 1:** Criteria for inclusion of studies.

Parameter	Criterion
Population	Adults aged ≥18 years
Intervention	Mediterranean or Mediterranean-style diet
Comparator	No treatment, usual care, or advice to follow a different diet
Outcome	(1) Metabolic syndrome incidence; (2) Metabolic syndrome components and additional risk factors (anthropometric and biochemical markers, markers of oxidative stress, inflammation, endothelial function and insulin resistance); (3) Incidence and/or mortality from metabolic syndrome-related (type 2 diabetes, non-alcoholic fatty liver disease, cardiovascular diseases, such as coronary heart disease, stroke and heart failure, and cancer) and/or intermediate (e.g., pre-diabetes) comorbidities, and; (4) Outcomes related to medication/therapy received for metabolic syndrome components and metabolic syndrome-related comorbidities
Study design	Controlled trials

**Table 2:** Effect of the Mediterranean diet on anthropometric, blood pressure, biochemical, insulin resistance, oxidative stress, inflammatory, and endothelial function markers related to the metabolic syndrome *.

Outcome	No. of Studies	No. of Participants	Effect Estimate (MD, 95% CI)	*p*-Value	I^2^
**Anthropometric markers**					
Body weight (kg)	40	12,571	−1.72 (−2.40, −1.05)	<0.001	98.6%
Body mass index (kg/m^2^)	37	5679	−0.41 (−0.71, −0.10)	0.010	98.6%
Waist circumference (cm) (MetSyn component)	27	9690	−1.47 (−2.54, −0.39)	0.007	99.6%
Total fat mass (kg)	9	963	−0.47 (−1.53, 0.60)	0.389	85.1%
Total body fat (%)	8	661	−0.12 (−1.60, 1.37)	0.878	89.7%
**Blood pressure (MetSyn component)**					
Systolic blood pressure (mm Hg)	27	4930	−1.34 (−2.00, −0.67)	<0.001	93.6%
Diastolic blood pressure (mm Hg)	27	4930	−0.81 (−1.30, −0.32)	0.001	92.8%
**Biochemical and insulin resistance markers**					
Glucose (mg/dL) (MetSyn component)	31	3662	−2.98 (−4.54, −1.42)	<0.001	98.1%
Insulin (μU/mL)	20	2184	−0.94 (−1.72, −0.16)	0.019	97.2%
HOMA-IR index	18	2098	−0.42 (−0.70, −0.15)	0.003	97.7%
HbA1c (%)	18	869	−0.15 (−0.41, 0.12)	0.274	81.3%
Total cholesterol (mg/dL)	37	4603	−5.70 (−9.96, −1.43)	0.009	98.6%
LDL-cholesterol (mg/dL)	29	3633	−8.24 (−13.50, −2.99)	0.002	99.6%
HDL-cholesterol (mg/dL) (MetSyn component)	36	4433	1.30 (0.38, 2.21)	0.005	98.1%
Triglycerides (mg/dL) (MetSyn component)	38	4658	−12.30 (−15.60, −8.99)	<0.001	94.8%
Non-HDL-cholesterol (mg/dL)	2	584	−1.39 (−19.40, 16.61)	0.880	42.3%
Total:HDL-cholesterol ratio	6	670	−0.83 (−2.67, 1.01)	0.378	99.6%
Homocysteine (μmol/L)	2	171	−0.04 (−0.61, 0.53)	0.882	0.0%
AST (UI/L)	3	193	−3.44 (−7.56, 0.68)	0.102	97.7%
ALT (UI/L)	8	729	−5.66 (−9.44, −1.87)	0.003	97.3%
GGT (UI/L)	7	393	−2.51 (−5.38, 0.35)	0.086	63.7%
Hepatic fat mass (%)	3	224	−2.80 (−5.52, −0.08)	0.044	79.0%
**Oxidative stress markers**					
Oxidized LDL-cholesterol (U/L)	2	970	4.38 (−16.49, 25.25)	0.681	97.7%
**Inflammatory markers**					
C-reactive protein (mg/L)	13	1071	−0.77 (−1.14, −0.39)	<0.001	92.6%
Interleukin-6 (pg/mL)	4	261	−0.61 (−0.93, −0.30)	<0.001	0.0%
Adiponectin (μg/mL)	4	546	0.76 (−1.16, 2.67)	0.438	70.4%
Tumor necrosis factor-a (pg/mL)	2	283	−0.81 (−1.03, −0.60)	<0.001	0.0%
**Markers of endothelial function**					
Flow-mediated dilatation (%)	3	206	1.49 (0.61, 2.37)	<0.001	0.0%

ALT, alanine transaminase; AST, aspartame transaminase; CI, confidence intervals; GGT, gamma glutamyl transferase; HDL, high-density lipoprotein cholesterol; HbA1c, glycosylated hemoglobin; HOMA-IR, homeostatic model assessment of insulin resistance; LDL, low density lipoprotein cholesterol; MD, mean difference; MetSyn, metabolic syndrome. * Findings are based on random-effects meta-analysis (inverse variance). I^2^ represents the magnitude of heterogeneity.

**Table 3:** Effect of the Mediterranean diet on metabolic syndrome-related comorbidities and pharmacologic treatment for metabolic syndrome components and related comorbidities *.

Outcome	No. of Studies	Intervention		Control		Effect Estimate (RR, 95% CI)	*p*-Value	I^2^
		Events	Total	Events	Total			
**Metabolic Syndrome-related comorbidities**								
CVD mortality	3	90	5503	96	2955	0.72 (0.43, 1.01)	0.090	0.0%
CVD incidence	2	119	703	201	703	0.61 (0.42, 0.80)	<0.001	0.0%
Sudden cardiac death	2	15	703	34	703	0.45 (−0.15, 1.04)	0.142	0.0%
Stroke incidence	2	88	5496	71	2951	0.67 (0.35, 0.98)	<0.001	0.0%
Heart failure incidence	2	73	5470	67	2933	0.69 (0.08, 1.30)	0.300	59.4%
Non-fatal myocardial infarction	2	26	801	60	804	0.45 (−0.001, 0.900)	0.051	0.0%
Fatal myocardial infarction	2	30	703	44	703	0.68 (0.23, 1.12)	0.090	0.0%
Type 2 diabetes incidence	2	207	2598	144	1349	0.81 (0.61, 1.02)	0.051	0.0%
**Pharmacotherapy**								
Use of blood pressure lowering drugs	3	2444	3299	1130	1657	0.99 (0.96, 1.02)	0.550	0.0%
Use of lipid-lowering agents	2	1552	2738	602	1090	1.01 (0.95, 1.08)	0.690	0.0%
Use of anti-platelet therapy	2	818	2738	338	1090	0.99 (0.90, 1.08)	0.830	0.0%
Use of insulin	2	271	2738	109	1090	0.99 (0.78, 1.20)	0.890	0.0%
Use of oral antidiabetic agents	3	1112	2846	520	1197	0.83 (0.58, 1.09)	0.230	64.2%

CVD, cardiovascular disease; RR, risk ratio. * Findings are based on random-effects meta-analysis (inverse variance). I^2^ represents the magnitude of heterogeneity.

## Supplementary Materials

The following are available online at https://www.mdpi.com/2072-6643/12/11/3342/s1, Supplementary Materials Table S1: PRISMA checklist, Table S2: Search strategy, Table S3: Study selection criteria, Table S4: Table of excluded papers (*N* = 116), Table S5: Characteristics of included studies, Table S6: Foods provided to the intervention group, for papers reporting on studies that supplemented the dietary advice intervention with foods, Table S7: Summary of the findings on anthropometric and blood pressure markers (between-group differences) from the papers not included in the pooled analysis, Table S8: Summary of the findings on biochemical and markers of insulin resistance (between-group differences) from the papers (and/or outcomes) not included in the pooled analysis, Table S9: Summary of the findings on oxidative stress, inflammatory and endothelial function markers (between-group differences) from the papers (and/or outcomes) not included in the pooled analysis, Table S10: Summary of the findings on metabolic syndrome-related comorbidities (between-group differences) from the papers (and/or outcomes) not included in the pooled analysis, Table S11: Summary of the findings on metabolic syndrome and/or related comorbidity treatment (between-group differences) from the papers (and/or outcomes) not included in the pooled analysis, Table S12: Detailed risk of bias for each included paper reporting a non-randomised controlled trial, Table S13: Effect of the Mediterranean diet on anthropometric, blood pressure, biochemical, insulin resistance, oxidative stress, inflammatory and endothelial function markers related to the metabolic syndrome, according to different subgroups, Table S14: Effect of the Mediterranean diet on anthropometric, blood pressure, biochemical, insulin resistance, oxidative stress, inflammatory and endothelial function markers related to the metabolic syndrome (sensitivity analysis, following the exclusion of non-randomised controlled trials, cross-over trials and trials with ≥1000 participants), Figures S1–S28: Forest plots of controlled trials evaluating the effect of the Mediterranean diet on anthropometric, blood pressure, biochemical, insulin resistance, oxidative stress, inflammatory and endothelial function markers related to the metabolic syndrome, Figures S29–S36: Forest plots of controlled trials evaluating the effect of the Mediterranean diet on metabolic syndrome-related comorbidities, Figures S37–S41: Forest plots of controlled trials evaluating the effect of the Mediterranean diet on metabolic syndrome and/or related comorbidity treatment, Figure S42: Risk of bias for papers reporting a randomised controlled trial, Figures S43–S83: Funnel plots and Egger test of studies evaluating the effect of the Mediterranean diet on anthropometric, blood pressure, biochemical, insulin resistance, oxidative stress, inflammatory and endothelial function markers related to the metabolic syndrome, metabolic syndrome-related comorbidities and metabolic syndrome and/or related comorbidity treatment (SE, standard error).
