# Supplementary material for: The Effect of the Mediterranean Diet on Metabolic Health: A Systematic Review and Meta-Analysis of Controlled Trials in Adults
Source: Nutrients. 2020 Oct 30;12(11):3342. doi: 10.3390/nu12113342 (PMC7692768; doi:10.3390/nu12113342)
Supplement: Supplementary file 1 [file nutrients-12-03342-s001.pdf]

Supplementary **number and description**	**Page**
**Supplementary Table S1:** PRISMA checklist	2-3
**Supplementary Table S2:** Search strategy	4-5
**Supplementary Table S3:** Study selection criteria	6
**Supplementary Table S4:** Table of excluded papers (*N*=116)	7-10
**Supplementary Table S5:** Characteristics of included studies	11-18
**Supplementary Table S6:** Foods provided to the intervention group, for papers reporting on studies that supplemented the dietary advice intervention with foods	19-22
**Supplementary Table S7:** Summary of the findings on anthropometric and blood pressure markers (between-group differences) from the papers not included in the pooled analysis	23
**Supplementary Table S8:** Summary of the findings on biochemical and markers of insulin resistance (between-group differences) from the papers (and/or outcomes) not included in the pooled analysis	24-25
**Supplementary Table S9:** Summary of the findings on oxidative stress, inflammatory and endothelial function markers (between-group differences) from the papers (and/or outcomes) not included in the pooled analysis	26
**Supplementary Figures S1–S28:** Forest plots of controlled trials evaluating the effect of the Mediterranean diet on anthropometric, blood pressure, biochemical, insulin resistance, oxidative stress, inflammatory and endothelial function markers related to the metabolic syndrome	27-45
**Supplementary Figures S29–S36.** Forest plots of controlled trials evaluating the effect of the Mediterranean diet on metabolic syndrome-related comorbidities	46-49
**Supplementary Table S10:** Summary of the findings on metabolic syndrome-related comorbidities (between-group differences) from the papers (and/or outcomes) not included in the pooled analysis	50
**Supplementary Figures S37–S41.** Forest plots of controlled trials evaluating the effect of the Mediterranean diet on metabolic syndrome and/or related comorbidity treatment	51-53
**Supplementary Table S11:** Summary of the findings on metabolic syndrome and/or related comorbidity treatment (between-group differences) from the papers (and/or outcomes) not included in the pooled analysis	54
**Supplementary Figure S42:** Risk of bias for papers reporting a randomised controlled trial	55
**Supplementary Figures S43–S83:** Funnel plots and Egger test of studies evaluating the effect of the Mediterranean diet on anthropometric, blood pressure, biochemical, insulin resistance, oxidative stress, inflammatory and endothelial function markers related to the metabolic syndrome, metabolic syndrome-related comorbidities and metabolic syndrome and/or related comorbidity treatment (SE, standard error)	56-69
**Supplementary Table S12:** Detailed risk of bias for each included paper reporting a non-randomised controlled trial	70
**Supplementary Table S13:** Effect of the Mediterranean diet on anthropometric, blood pressure, biochemical, insulin resistance, oxidative stress, inflammatory and endothelial function markers related to the metabolic syndrome, according to different subgroups	71-87
**Supplementary Table S14:** Effect of the Mediterranean diet on anthropometric, blood pressure, biochemical, insulin resistance, oxidative stress, inflammatory and endothelial function markers related to the metabolic syndrome (sensitivity analysis, following the exclusion of non-randomised controlled trials, cross-over trials and trials with ≥1,000 participants)	88-92

Supplementary Table S1. PRISMA checklist

Section/topic	#	Checklist item	Reported on page #
**TITLE**			
Title	1	Identify the report as a systematic review, meta-analysis, or both.	Title page
**ABSTRACT**			
Structured summary	2	Provide a structured summary including, as applicable: background; objectives; data sources; study eligibility criteria, participants, and interventions; study appraisal and synthesis methods; results; limitations; conclusions and implications of key findings; systematic review registration number.	Abstract
**INTRODUCTION**			
Rationale	3	Describe the rationale for the review in the context of what is already known.	2
Objectives	4	Provide an explicit statement of questions being addressed with reference to participants, interventions, comparisons, outcomes, and study design (PICOS).	2
**METHODS**			
Protocol and registration	5	Indicate if a review protocol exists, if and where it can be accessed (e.g., Web address), and, if available, provide registration information including registration number.	Abstract and p 2
Eligibility criteria	6	Specify study characteristics (e.g., PICOS, length of follow-up) and report characteristics (e.g., years considered, language, publication status) used as criteria for eligibility, giving rationale.	3 and Supplement: Study Selection Criteria
Information sources	7	Describe all information sources (e.g., databases with dates of coverage, contact with study authors to identify additional studies) in the search and date last searched.	2-3 and Supplement: Search Strategy
Search	8	Present full electronic search strategy for at least one database, including any limits used, such that it could be repeated.	Supplement: Search Strategy
Study selection	9	State the process for selecting studies (i.e., screening, eligibility, included in systematic review, and, if applicable, included in the meta-analysis).	3-4 and Supplement: Study Selection Criteria
Data collection process	10	Describe method of data extraction from reports (e.g., piloted forms, independently, in duplicate) and any processes for obtaining and confirming data from investigators.	4
Data items	11	List and define all variables for which data were sought (e.g., PICOS, funding sources) and any assumptions and simplifications made.	3-5 and Supplement: Study Selection Criteria
Risk of bias in individual studies	12	Describe methods used for assessing risk of bias of individual studies (including specification of whether this was done at the study or outcome level), and how this information is to be used in any data synthesis.	4
Summary measures	13	State the principal summary measures (e.g., risk ratio, difference in means).	4-5
Synthesis of results	14	Describe the methods of handling data and combining results of studies, if done, including measures of consistency (e.g., I^2) for each meta-analysis.	4-5

Section/topic	#	Checklist item	Reported on page #
Risk of bias across studies	15	Specify any assessment of risk of bias that may affect the cumulative evidence (e.g., publication bias, selective reporting within studies).	4-5
Additional analyses	16	Describe methods of additional analyses (e.g., sensitivity or subgroup analyses, meta-regression), if done, indicating which were pre-specified.	5
**RESULTS**			
Study selection	17	Give numbers of studies screened, assessed for eligibility, and included in the review, with reasons for exclusions at each stage, ideally with a flow diagram.	5, Figure 1 and Supplement: Table of excluded papers
Study characteristics	18	For each study, present characteristics for which data were extracted (e.g., study size, PICOS, follow-up period) and provide the citations.	5-6, and Supplement: Table 3
Risk of bias within studies	19	Present data on risk of bias of each study and, if available, any outcome level assessment (see item 12).	10 and Supplement: Figures 43-83
Results of individual studies	20	For all outcomes considered (benefits or harms), present, for each study: (a) simple summary data for each intervention group (b) effect estimates and confidence intervals, ideally with a forest plot.	6-8, Tables 2-3 and Supplement: Figures 1-28, 29-36 and 37-41 and Supplement: Tables 5-7
Synthesis of results	21	Present results of each meta-analysis done, including confidence intervals and measures of consistency.	6-8, Tables 2-3 and Supplement: Figures 1-28, 29-36 and 37-41
Risk of bias across studies	22	Present results of any assessment of risk of bias across studies (see Item 15).	10, Supplement: Figure 42
Additional analysis	23	Give results of additional analyses, if done (e.g., sensitivity or subgroup analyses, meta-regression [see Item 16]).	10-11 and Supplement: Tables 11-12
**DISCUSSION**			
Summary of evidence	24	Summarize the main findings including the strength of evidence for each main outcome; consider their relevance to key groups (e.g., healthcare providers, users, and policy makers).	11-14
Limitations	25	Discuss limitations at study and outcome level (e.g., risk of bias), and at review-level (e.g., incomplete retrieval of identified research, reporting bias).	11-14
Conclusions	26	Provide a general interpretation of the results in the context of other evidence, and implications for future research.	11-14
**FUNDING**			
Funding	27	Describe sources of funding for the systematic review and other support (e.g., supply of data); role of funders for the systematic review.	p 15

Supplementary Table S2. Search strategy

Search strategy (databases searched from inception until 17th June 2019)

Strategy for Pubmed (field labels were used to restrict specific terms/phrases to Medical Subject Headings [MeSH] or by publication type [pt] and title/abstract [tiab] fields)
1. "mediterranean diet"[tiab] or "mediterranean lifestyle"[tiab] or "mediterranean dietary pattern"[tiab] or "mediterranean style diet"[tiab] or "mediterranean-style diet"[tiab] or "mediterranean diet score"[tiab] or "mediterranean diet index"
2. Diet, Mediterranean [MeSH]
3. "metabolic syndrome"[tiab] or "metabolic risk"[tiab] or "metabolic risks"[tiab] or "metabolic markers"[tiab] or "cardiovascular risk factors"[tiab] or "cardiovascular disease risk"[tiab] or "cardiovascular disease risks"[tiab] or "vascular markers"[tiab] or "adiposity"[tiab] or "overweight"[tiab] or "obesity"[tiab] or "obese"[tiab] or "body weight"[tiab] or "weight"[tiab] or "abdominal fat"[tiab] or "body composition"[tiab] or "BMI"[tiab] or "body mass"[tiab] or "waist circumference"[tiab] or "weight loss"[tiab] or "blood pressure"[tiab] or "cholesterol"[tiab] or "triglycerides"[tiab] or "inflammation"[tiab] or "inflammatory markers"[tiab] or "insulin resistance"[tiab] or "oxidative stress"[tiab] or "endothelial function"[tiab]
4. "type 2 diabetes"[tiab] or "non-alcoholic fatty liver disease"[tiab] or "nonalcoholic fatty liver disease"[tiab] or "non alcoholic fatty liver disease"[tiab] or "NAFLD"[tiab] or "non-alcoholic steatohepatitis"[tiab] or "nonalcoholic steatohepatitis"[tiab] or "non alcoholic steatohepatitis"[tiab] or "NASH"[tiab] or "cardiovascular disease"[tiab] or "cardiovascular diseases"[tiab] or "heart disease"[tiab] or "heart diseases"[tiab] or "coronary heart disease"[tiab] or "coronary artery disease"[tiab] or "stroke"[tiab] or "heart failure"[tiab] or "myocardial infarction"[tiab] or "cancer"[tiab]
5. Metabolic Syndrome [MeSH] or Inflammation [MeSH] or Insulin Resistance [MeSH] or Oxidative Stress [MeSH]
6. Obesity [MeSH] or Obesity, Abdominal [MeSH] or Weight loss [MeSH] or Adiposity [MeSH] or Diabetes mellitus, type 2 [MeSH] or Non-alcoholic Fatty Liver Disease [MeSH] or Cardiovascular diseases [MeSH] or Heart Diseases [MeSH] or Coronary Disease [MeSH] or Coronary Artery Disease [MeSH] or Stroke [MeSH] or Cerebrovascular disorders [MeSH] or Heart failure [MeSH] or Myocardial infarction [MeSH] or Neoplasms [MeSH]
7. "Intervention"[tiab] or "interventions"[tiab] or "controlled trial"[tiab] or "controlled trials"[tiab] or "clinical trial"[tiab] or "RCT"[tiab] or "RCTs"[tiab] or "randomized controlled trial"[tiab] or "randomised controlled trial"[tiab] or "programme"[tiab] or "program"[tiab]
8. "Cross-Sectional Studies"[MeSH] or Editorial [pt] or Letter [pt] or "case control" [tiab] or "case study" [tiab] or "Case-Control Studies" [MeSH] or "prospective cohort" [tiab] or "cohort studies" [MeSH] or "cohort study" [tiab] or "Longitudinal Studies" [MeSH]
9. #1 or #2
10. #3 or #4 or #5 or #6
Final search: #9 AND #10 AND #7 NOT #8
Results: 792

Strategy for Embase (field labels were used to restrict specific terms/phrases by publication type and title/abstract/keyword [ti,ab,kw] fields).
1. ("mediterranean diet$" or "mediterranean lifestyle$" or "mediterranean dietary pattern" or "mediterranean style diet$" or "mediterranean-style diet$" or "mediterranean diet score" or "mediterranean diet index").ti,ab,kw.
2. ("metabolic syndrome" or "metabolic risk$" or "metabolic marker$" or "cardiovascular risk factors" or "cardiovascular disease risk$" or "vascular marker$" or adiposity or overweight or obesity or obese or "body weight" or weight or "abdominal fat" or "abdominal obesity" or "body composition" or BMI or "body mass" or "waist circumference" or "weight loss" or "blood pressure" or cholesterol or triglycerides or inflammation or "inflammatory marker$" or "insulin resistance" or "oxidative stress" or "endothelial function" or "type 2 diabetes" or "non-alcoholic fatty liver disease$" or "nonalcoholic fatty liver disease$" or "non alcoholic fatty liver disease$" or NAFLD or "non-alcoholic steatohepatitis" or "nonalcoholic steatohepatitis" or "non alcoholic steatohepatitis" or NASH or "cardiovascular disease$" or "heart disease$" or "coronary heart disease" or "coronary disease$" or "coronary artery disease$" or stroke or "cerebrovascular disease$" or "heart failure" or "myocardial infarction" or cancer$ or neoplasm$).ti,ab,kw.
3. (Intervention$ or "controlled trial$" or "clinical trial$" or RCT$ or "randomized controlled trial$" or "randomised controlled trial$" or programme$ or program$ not "Cross-Sectional Studies" not "case control" not "case study" not "case-control$" not "prospective cohort$" not "cohort study" not "cohort studies" not "longitudinal studies").ti,ab,kw.
Final search: #1 AND #2 AND #3
Results: 1500

Strategy for Web of Science (All terms were searched in "Topic").
1. "mediterranean diet*" or "mediterranean lifestyle*" or "mediterranean dietary pattern" or "mediterranean style diet*" or "mediterranean-style diet*" or "mediterranean diet score" or "mediterranean diet index"
2. "metabolic syndrome" or "metabolic risk*" or "metabolic marker*" or "cardiovascular risk factors" or "cardiovascular disease risk*" or "vascular marker*" or adiposity or overweight or obesity or obese or "body weight" or weight or "abdominal fat" or "abdominal obesity" or "body composition" or BMI or "body mass" or "waist circumference" or "weight loss" or "blood pressure" or cholesterol or triglycerides or inflammation or "inflammatory marker*" or "insulin resistance" or "oxidative stress" or "endothelial function" or "type 2 diabetes" or "non-alcoholic fatty liver disease*" or "nonalcoholic fatty liver disease*" or "non alcoholic fatty liver disease*" or NAFLD or "non-alcoholic steatohepatitis" or "nonalcoholic steatohepatitis" or "non alcoholic steatohepatitis" or NASH or

"cardiovascular disease*" or "heart disease*" or "coronary heart disease" or "coronary disease*" or "coronary artery disease*" or stroke or "cerebrovascular disease*" or "heart failure" or "myocardial infarction" or cancer* or neoplasm*
3. intervention* or "controlled trial*" or "clinical trial*" or "RCT*" or "randomized controlled trial*" or "randomised controlled trial*" or programme* or program*
4. "Cross-Sectional Studies" or Editorial or Letter or "case control" or "case study" or "case-control studies" or "prospective cohort" or "cohort study" or "cohort studies" or "longitudinal studies"
Final search: #1 AND #2 AND #3 NOT #4 (No limitations were used)
Results: 1877

Strategy for CINAHL (field labels were used to restrict specific terms/phrases by publication type and title/abstract fields)
1. TI ("mediterranean diet" or "mediterranean lifestyle" or "mediterranean dietary pattern" or "mediterranean style diet" or "mediterranean-style diet" or "mediterranean diet score" or "mediterranean diet index") OR AB ("mediterranean diet" or "mediterranean lifestyle" or "mediterranean dietary pattern" or "mediterranean style diet" or "mediterranean-style diet" or "mediterranean diet score" or "mediterranean diet index")
2. TI ("metabolic syndrome" or "metabolic risk" or "metabolic risks" or "metabolic markers" or "cardiovascular risk factors" or "cardiovascular disease risk" or "cardiovascular disease risks" or "vascular markers" or adiposity or overweight or obesity or obese or "body weight" or weight or "abdominal fat" or "abdominal obesity" or "body composition" or BMI or "body mass" or "waist circumference" or "weight loss" or "blood pressure" or cholesterol or triglycerides or inflammation or "inflammatory markers" or "insulin resistance" or "oxidative stress" or "endothelial function" or "type 2 diabetes" or "non-alcoholic fatty liver disease" or "nonalcoholic fatty liver disease" or "non alcoholic fatty liver disease" or NAFLD or "non-alcoholic steatohepatitis" or "nonalcoholic steatohepatitis" or "non alcoholic steatohepatitis" or NASH or "cardiovascular disease" or "cardiovascular diseases" or "heart disease" or "heart diseases" or "coronary heart disease" or "coronary disease" or "coronary artery disease" or stroke or "cerebrovascular disease" or "heart failure" or "myocardial infarction" or cancer or neoplasms) OR AB ("metabolic syndrome" or "metabolic risk" or "metabolic risks" or "metabolic markers" or "cardiovascular risk factors" or "cardiovascular disease risk" or "cardiovascular disease risks" or "vascular markers" or adiposity or overweight or obesity or obese or "body weight" or weight or "abdominal fat" or "abdominal obesity" or "body composition" or BMI or "body mass" or "waist circumference" or "weight loss" or "blood pressure" or cholesterol or triglycerides or inflammation or "inflammatory markers" or "insulin resistance" or "oxidative stress" or "endothelial function" or "type 2 diabetes" or "non-alcoholic fatty liver disease" or "nonalcoholic fatty liver disease" or "non alcoholic fatty liver disease" or NAFLD or "non-alcoholic steatohepatitis" or "nonalcoholic steatohepatitis" or "non alcoholic steatohepatitis" or NASH or "cardiovascular disease" or "cardiovascular diseases" or "heart disease" or "heart diseases" or "coronary heart disease" or "coronary disease" or "coronary artery disease" or stroke or "cerebrovascular disease" or "heart failure" or "myocardial infarction" or cancer or neoplasms)
3. TI (intervention or interventions or "controlled trial" or "controlled trials" or "clinical trial" or RCT or RCTs or "randomized controlled trial" or "randomised controlled trial" or programme or program) OR AB (intervention or interventions or "controlled trial" or "controlled trials" or "clinical trial" or RCT or RCTs or "randomized controlled trial" or "randomised controlled trial" or programme or program)
4. PT (Editorial or Letter)
5. TI ("cross-sectional" or "case control" or "case study" or "case-control" or "prospective cohort" or "cohort study" or "longitudinal") OR AB ("cross-sectional" or "case control" or "case study" or "case-control" or "prospective cohort" or "cohort study" or "longitudinal")
Final search: #1 AND #2 AND #3 NOT #4 NOT #5 (limit to humans)
Results: 189

Supplementary Table S3. Study selection criteria

Population

Studies were included in the review if they involved adults (aged ≥18 years), including those with established metabolic syndrome (MetSyn), metabolic risk factors (e.g. hyperlipidemia) and/or MetSyn-related comorbidities, e.g. type 2 diabetes, non-alcohol fatty liver disease (NAFLD), cardiovascular disease (CVD), cancer. Studies were excluded if participants were children and/or adolescents, pregnant or lactating women and adults with psychiatric conditions, HIV or conditions that might affect the ability to eat certain foods, such as asthma, renal failure or diseases of the gastrointestinal tract.

Intervention(s)

Studies were included if the intervention group received an intervention promoting the whole Mediterranean diet (MD) or MD-style diet. As physical activity was an integral component of the traditional Mediterranean lifestyle and forms an essential component of lifestyle modification for MetSyn prevention and management, studies were also eligible if the intervention promoted the MD concurrently with physical activity, as long as physical activity was equally promoted in the control group. Studies in which the intervention contained further components, such as stress management or smoking cessation, or in which there was a focus on specific foods or components of the MD (instead of the whole diet), were excluded.

Comparison(s)

Studies were included if the comparator/control group received no treatment, usual care, or advice to follow a different diet (e.g. a low-fat diet), with or without physical activity, as long as physical activity was also promoted equally to the intervention group. Studies were excluded if they did not have a control group, or if the MD was promoted to both the intervention and control groups.

Outcome(s)

We specifically focused on outcomes that are commonly assessed or reported in everyday clinical practice in order to enhance relevance and the translational potential of the findings to practitioners and clinicians. Studies were included if they reported at least one of the following, either as a primary or secondary outcome: 1) MetSyn incidence; 2) MetSyn components (waist circumference, systolic and/or diastolic blood pressure, and blood concentrations of HDL-cholesterol, triglycerides and fasting glucose), and additional risk factors, such as body weight, body mass index, body composition (total fat mass and % body fat), and blood concentrations of insulin, glycosylated haemoglobin, total- and LDL-cholesterol, markers of inflammation and endothelial function (e.g. C-reactive protein, interleukin (IL)-6, adiponectin, tumor necrosis factor α, or flow-mediated dilatation), markers of insulin resistance (e.g. homeostatic model assessment-insulin resistance and markers of oxidative stress (e.g. oxidised LDL-cholesterol or total antioxidant capacity); 3) Incidence and/or mortality from MetSyn comorbidities, including type 2 diabetes, NAFLD, CVD, such as coronary heart disease, stroke and heart failure, and cancer), and; 4) Outcomes related to medication/therapy received for these comorbidities (e.g. % of participants receiving medication post-intervention).

Study design

All original controlled trials (randomised and non-randomised), reporting pre- and post-intervention findings for the outcomes of interest and of any length of further follow-up were eligible. Studies with different methodological designs (e.g. cohort, case-control, cross-sectional etc), peer-reviewed study protocols that did not report preliminary findings, book chapters, editorials and conference abstracts were excluded.

Supplementary Table S4. Table of excluded papers (*N*=116)

Author	Title	Reason
No author listed	A novel model of clinical exercise delivery reduces blood pressure in hypertensive individuals	Ineligible study design
Abenavoli, *et al.*	Effect of Mediterranean Diet and Antioxidant Formulation in Non-Alcoholic Fatty Liver Disease: A Randomized Study	Ineligible type of intervention/ control
Abendroth, *et al.*	Changes of Intestinal Microflora in Patients with Rheumatoid Arthritis during Fasting or a Mediterranean Diet	Could not retrieve full text
Alonso-Dominguez, *et al.*	Effectiveness of a multifactorial intervention in increasing adherence to the mediterranean diet among patients with diabetes mellitus type 2: A controlled and randomized study (EMID study)	Ineligible type of intervention/ control
Andreoli, *et al.*	Effect of a moderately hypoenergetic Mediterranean diet and exercise program on body cell mass and cardiovascular risk factors in obese women	Ineligible study design
Avellone, *et al.*	Effects of Mediterranean diet on lipid, coagulative and fibrinolytic parameters in two randomly selected population samples in Western Sicily	Could not retrieve full text
Avellone, *et al.*	Cross-over study on effects of Mediterranean diet in two randomly selected population samples	Unusable data
Bekkouche, *et al.*	The mediterranean diet adoption improves metabolic, oxidative, and inflammatory abnormalities in Algerian metabolic syndrome patients	Ineligible outcomes
Blanco-Rojo, *et al.*	The insulin resistance phenotype (muscle or liver) interacts with the type of diet to determine changes in disposition index after 2 years of intervention: the CORDIOPREV-DIAB randomised clinical trial	Ineligible outcomes
Boidin, *et al.*	Effect of aquatic interval training with Mediterranean diet counseling in obese patients: Results of a preliminary study	Ineligible type of intervention/ control
Bonfanti, *et al.*	Effect of two hypocaloric diets and their combination with physical exercise on basal metabolic rate and body composition	Ineligible language
Brauer, *et al.*	Nutrient Intake and Dietary Quality Changes within a Personalized Lifestyle Intervention Program for Metabolic Syndrome in Primary Care	Ineligible outcomes
Bruno, *et al.*	Adherence to Mediterranean Diet and Metabolic Syndrome in BRCA Mutation Carriers	Ineligible type of intervention/ control
Bullo, *et al.*	Mediterranean Diet and High Dietary Acid Load Associated with Mixed Nuts: Effect on Bone Metabolism in Elderly Subjects	Refers to the PREDIMED study, less complete outcomes than the primary sources
Canfi, *et al.*	Effect of changes in the intake of weight of specific food groups on successful body weight loss during a multi-dietary strategy intervention trial	Ineligible outcomes
Casas, *et al.*	Anti-Inflammatory Effects of the Mediterranean Diet in the Early and Late Stages of Atheroma Plaque Development	Refers to the PREDIMED study, less complete outcomes than the primary sources
Casas, *et al.*	The Effects of the Mediterranean Diet on Biomarkers of Vascular Wall Inflammation and Plaque Vulnerability in Subjects with High Risk for Cardiovascular Disease. A Randomized Trial	Refers to the PREDIMED study, less complete outcomes than the primary sources
Clements, *et al.*	Age-Associated Decline in Dendritic Cell Function and the Impact of Mediterranean Diet Intervention in Elderly Subjects	Ineligible outcomes
Corella, *et al.*	Mediterranean diet reduces the adverse effect of the TCF7L2-rs7903146 polymorphism on cardiovascular risk factors and stroke incidence: A randomized controlled trial in a high-cardiovascular-risk population	Ineligible outcomes
Cueto-Galán, *et al.*	Changes in fatty liver index after consuming a Mediterranean diet: 6-Year follow-up of the PREDIMED-Malaga trial	Unusable data
Damasceno, *et al.*	Mediterranean diet supplemented with nuts reduces waist circumference and shifts lipoprotein subfractions to a less atherogenic pattern in subjects at high cardiovascular risk	Refers to the PREDIMED study, less complete outcomes than the primary sources
Davis, *et al.*	Older Australians Can Achieve High Adherence to the Mediterranean Diet during a 6 Month Randomised Intervention; Results from the Medley Study	Ineligible outcomes
de la Puebla, *et al.*	A reduction in dietary saturated fat decreases body fat content in overweight, hypercholesterolemic males	Unusable data
De Lorenzo, *et al.*	Mediterranean meal versus Western meal effects on postprandial ox-LDL, oxidative and inflammatory gene expression in healthy subjects: a randomized controlled trial for nutrigenomic approach in cardiometabolic risk	Ineligible type of intervention/ control
Delgado-Lista, *et al.*	CORonary Diet Intervention with Olive oil and cardiovascular PREVention study (the CORDIOPREV study): Rationale, methods, and baseline characteristics: A clinical trial comparing the efficacy of a Mediterranean diet rich in olive oil versus a low-fat diet on cardiovascular disease in coronary patients	Unusable data
Dewell, *et al.*	Antioxidants from diet or supplements do not alter inflammatory markers in adults with cardiovascular disease risk. A pilot randomized controlled trial	Ineligible type of intervention/ control
Di Renzo, *et al.*	Influence of FTO rs9939609 and Mediterranean diet on body composition and weight loss: a randomized clinical trial	Ineligible outcomes
Djuric, *et al.*	A Mediterranean dietary intervention in healthy American women changes plasma carotenoids and fatty acids in distinct clusters	Ineligible type of intervention/ control
Djuric, *et al.*	Effects of a Mediterranean Diet Intervention on Anti- and Pro-Inflammatory Eicosanoids, Epithelial Proliferation, and Nuclear Morphology in Biopsies of Normal Colon Tissue	Ineligible type of intervention/ control
Domenech, *et al.*	Mediterranean Diet Reduces 24-Hour Ambulatory Blood Pressure, Blood Glucose, and Lipids: One-Year Randomized, Clinical Trial	Refers to the PREDIMED study, less complete outcomes than the primary sources
Due, *et al.*	The effect of three different ad libitum diets for weight loss maintenance: a randomized 18-month trial	Ineligible type of intervention/ control

Ellsworth, *et al.*	Lifestyle modification interventions differing in intensity and dietary stringency improve insulin resistance through changes in lipoprotein profiles	Ineligible type of intervention/ control
Errazuriz, *et al.*	Randomized Controlled Trial of a MUFA or Fiber-Rich Diet on Hepatic Fat in Prediabetes	Ineligible type of intervention/ control
Esposito, *et al.*	Long-term effect of mediterranean-style diet and calorie restriction on biomarkers of longevity and oxidative stress in overweight men	Ineligible type of intervention/ control
Esposito, *et al.*	Synergistic Interplay between Curcumin and Polyphenol-Rich Foods in the Mediterranean Diet: Therapeutic Prospects for Neurofibromatosis 1 Patients	Ineligible type of paper
Fito, *et al.*	Effect of a Traditional Mediterranean Diet on Lipoprotein Oxidation: A Randomized Controlled Trial	Refers to the same study as Fito et al (2014), less complete outcomes than the primary source
Fuentes, *et al.*	Mediterranean and Low-Fat Diets Improve Endothelial Function in Hypercholesterolemic Men	Unusable data
Gadgil, *et al.*	The Effects of Carbohydrate, Unsaturated Fat, and Protein Intake on Measures of Insulin Sensitivity	Ineligible type of intervention/ control
Garaulet, *et al.*	CLOCK gene is implicated in weight reduction in obese patients participating in a dietary programme based on the Mediterranean diet	Ineligible outcomes
Garcia-Rios, *et al.*	Beneficial effect of CLOCK gene polymorphism rs1801260 in combination with low-fat diet on insulin metabolism in the patients with metabolic syndrome	Ineligible outcomes
Garcia-Silva, *et al.*	Efficacy of Cognitive Behavioral Therapy in Adherence to the Mediterranean Diet in Metabolic Syndrome Patients: A Randomized Controlled Trial	Ineligible type of intervention/ control
Gelli, *et al.*	Effect of a counseling-supported treatment with the Mediterranean diet and physical activity on the severity of the non-alcoholic fatty liver disease	Ineligible study design
Gepner, *et al.*	Intramyocellular triacylglycerol accumulation across weight loss strategies; Sub-study of the CENTRAL trial	Ineligible outcomes
Gepner, *et al.*	The beneficial effects of Mediterranean diet over low-fat diet may be mediated by decreasing hepatic fat content	Refers to the same study as Gepner et al (2018), less complete outcomes than the primary sources
Giallauria, *et al.*	Exercise training improves heart rate recovery in women with breast cancer	Ineligible type of intervention/ control
Giallauria, *et al.*	Exercise training improves cardiopulmonary and endothelial function in women with breast cancer: findings from the Diana-5 dietary intervention study	Ineligible type of intervention/ control
Gomez-Huelgas, *et al.*	Impact of Intensive Lifestyle Modification on Levels of Adipokines and Inflammatory Biomarkers in Metabolically Healthy Obese Women	Ineligible study design
Gomez-Marin *et al.*	Long-term consumption of a Mediterranean diet improves postprandial lipemia in patients with type 2 diabetes: the Cordioprev randomized trial	Unusable data
Granata, *et al.*	Dietary Enterolactone Affects Androgen and Estrogen Levels in Healthy Postmenopausal Women	Ineligible outcomes
Grimaldi, *et al.*	Intensive dietary intervention promoting the Mediterranean diet in people with high cardiometabolic risk: a non-randomized study	Ineligible type of intervention/ control
Hernaez, *et al.*	Mediterranean Diet Improves High-Density Lipoprotein Function in High-Cardiovascular-Risk Individuals: A Randomized Controlled Trial	Refers to the PREDIMED study, less complete outcomes than the primary sources
Hernaez, *et al.*	The Mediterranean Diet decreases LDL atherogenicity in high cardiovascular risk individuals: a randomized controlled trial	Refers to the PREDIMED study, less complete outcomes than the primary sources
Ijzelenberg, *et al.*	The effect of a comprehensive lifestyle intervention on cardiovascular risk factors in pharmacologically treated patients with stable cardiovascular disease compared to usual care: a randomised controlled trial	Ineligible type of intervention/ control
Itsiopoulos, *et al.*	Can the Mediterranean diet lower HbA1c in type 2 diabetes? Results from a randomized cross-over study	Unusable data
Jeejeebhoy, *et al.*	Family physician-led, team-based, lifestyle intervention in patients with metabolic syndrome: results of a multicentre feasibility project	Ineligible study design
Jennings, *et al.*	A Mediterranean-like dietary pattern with vitamin D3 (10 microg/d) supplements reduced the rate of bone loss in older Europeans with osteoporosis at baseline: results of a 1-y randomized controlled trial	Ineligible outcomes
Jennings, *et al.*	Effectiveness of a preventive cardiology programme for high CVD risk persistent smokers: the EUROACTION PLUS varenicline trial	Ineligible type of intervention/ control
Kiechle, *et al.*	Feasibility of structured endurance training and Mediterranean diet in BRCA1 and BRCA2 mutation carriers - an interventional randomized controlled multicenter trial (LIBRE-1)	Ineligible type of intervention/ control
Klein, *et al.*	The CHANGE program Exercise intervention in primary care	Ineligible type of intervention/ control
Klonizakis, *et al.*	Long-term effects of an exercise and Mediterranean diet intervention in the vascular function of an older, healthy population	Ineligible type of intervention/ control
Klonizakis, *et al.*	Mediterranean diet- and exercise-induced improvement in age-dependent vascular activity	Ineligible type of intervention/ control
Knight, *et al.*	A randomised controlled intervention trial evaluating the efficacy of a Mediterranean dietary pattern on cognitive function and psychological wellbeing in healthy older adults: the MedLey study	Ineligible type of paper
Korre, *et al.*	Survival Mediterranean Style: Lifestyle Changes to Improve the Health of the US Fire Service	Ineligible type of paper
Laake, *et al.*	Effects on Serum Fractalkine by Diet and Omega-3 Fatty Acid Intervention: Relation to Clinical Outcome	Ineligible type of intervention/ control
Leighton, *et al.*	Plasma polyphenols and antioxidants, oxidative DNA damage and endothelial function in a diet and wine intervention study in humans	Ineligible type of paper

Leighton, *et al.*	Health impact of Mediterranean diets in food at work	Ineligible study design
Lindman, *et al.*	The effects of long-term diet and omega-3 fatty acid supplementation on coagulation factor VII and serum phospholipids with special emphasis on the R353Q polymorphism of the FVII gene	Refers to the same study as Hjerkinn et al, Troseid et al, less complete outcomes than the primary sources
Lombardo, *et al.*	Morning Meal More Efficient for Fat Loss in a 3-Month Lifestyle Intervention	Ineligible type of intervention/ control
Lopez-Moreno, *et al.*	Mediterranean Diet Supplemented With Coenzyme Q10 Modulates the Postprandial Metabolism of Advanced Glycation End Products in Elderly Men and Women	Ineligible outcomes
Maciejewska, *et al.*	Seeking Optimal Nutrition for Healthy Body Mass Reduction among Former Athletes	Ineligible outcomes
Marcos-Forniol, *et al.*	Secondary prevention programme of ischaemic heart disease in the elderly: A randomised clinical trial	Ineligible type of intervention/ control
Marques-Rocha, *et al.*	Expression of inflammation-related miRNAs in white blood cells from subjects with metabolic syndrome after 8 wk of following a Mediterranean diet-based weight loss program	Ineligible study design
Mayneris-Perxachs, *et al.*	Effects of 1-Year Intervention with a Mediterranean Diet on Plasma Fatty Acid Composition and Metabolic Syndrome in a Population at High Cardiovascular Risk	Ineligible outcomes
Mayr, *et al.*	Improvement in dietary inflammatory index score after 6-month dietary intervention is associated with reduction in interleukin-6 in patients with coronary heart disease: The AUSMED heart trial	Ineligible study design
Mena, *et al.*	Inhibition of circulating immune cell activation: a molecular antiinflammatory effect of the Mediterranean diet	Refers to the PREDIMED study, less complete outcomes than the primary sources
Mezzano, *et al.*	Mediterranean diet, but not red wine, is associated with beneficial changes in primary haemostasis	Ineligible outcomes
Michalsen, *et al.*	Effects of lifestyle modification on the progression of coronary atherosclerosis, autonomic function, and angina - The role of GNB3 C825T polymorphism	Ineligible type of intervention/ control
Michalsen, *et al.*	Mediterranean diet has no effect on markers of inflammation and metabolic risk factors in patients with coronary artery disease	Ineligible type of intervention/ control
Mitjavila, *et al.*	The Mediterranean diet improves the systemic lipid and DNA oxidative damage in metabolic syndrome individuals. A randomized, controlled, trial	Ineligible outcomes
Mlakar, *et al.*	The effect of cardioprotective diet rich with natural antioxidants on chronic inflammation and oxidized LDL during cardiac rehabilitation in patients after acute myocardial infarction	Ineligible type of intervention/ control
Monlezun, *et al.*	Medical school-based teaching kitchen improves HbA1c, blood pressure, and cholesterol for patients with type 2 diabetes: Results from a novel randomized controlled trial	Unusable data
Murie-Fernandez, *et al*	Carotid intima-media thickness changes with Mediterranean diet: A randomized trial (PREDIMED-Navarra)	Refers to the PREDIMED study, less complete outcomes than the primary sources
Panunzio, *et al.*	Randomized, controlled nutrition education trial promotes a Mediterranean diet and improves anthropometric, dietary, and metabolic parameters in adults	Ineligible language
Paoli, *et al.*	Long Term Successful Weight Loss with a Combination Biphasic Ketogenic Mediterranean Diet and Mediterranean Diet Maintenance Protocol	Ineligible type of intervention/ control
Pasanisi, *et al.*	A dietary intervention to lower serum levels of IGF-I in BRCA mutation carriers	Ineligible type of intervention/ control
Perez-Jimenez, *et al.*	A Mediterranean and a high-carbohydrate diet improve glucose metabolism in healthy young persons	Unusable data
Perez-Martinez, *et al.*	Consumption of diets with different type of fat influences triacylglycerols-rich lipoproteins particle number and size during the postprandial state	Unusable data
Perona, *et al.*	Reduction in systemic and VLDL triacylglycerol concentration after a 3-month Mediterranean-style diet in high-cardiovascular-risk subjects	Ineligible outcomes
Porenta, *et al.*	Interaction of Fatty Acid Genotype and Diet on Changes in Colonic Fatty Acids in a Mediterranean Diet Intervention Study	Ineligible outcomes
Porenta, *et al.*	Correction: Interaction of fatty acid genotype and diet on changes in colonic fatty acids in a Mediterranean diet intervention study (Cancer Prevention Research (2013) 6, (1212-1221))	Ineligible outcomes
Razquin, *et al.*	A 3 years follow-up of a Mediterranean diet rich in virgin olive oil is associated with high plasma antioxidant capacity and reduced body weight gain	Refers to the same study as Storniolo et al (2017), less complete outcomes than the primary source
Renaud, *et al.*	Cretan Mediterranean diet for prevention of coronary heart disease	Ineligible outcomes
Richard, *et al.*	Effect of Mediterranean Diet With and Without Weight Loss on Apolipoprotein B100 Metabolism in Men With Metabolic Syndrome	Refers to the same study as Richard et al (2011, 2013), less complete outcomes than the primary sources
Roncero-Ramos, *et al.*	Prediabetes diagnosis criteria, type 2 diabetes risk and dietary modulation: The CORDIOPREV study	Unusable data
Salas-Salvado, *et al.*	Effect of a Mediterranean Diet Supplemented With Nuts on Metabolic Syndrome Status: *One-Year Results of the PREDIMED Randomized Trial*	Refers to the same study as Babio et al (2014), less complete outcomes than the primary source
Salas-Salvado, *et al.*	Reduction in the Incidence of Type 2 Diabetes With the Mediterranean Diet: Results of the PREDIMED-Reus nutrition intervention randomized trial	Refers to the same study as Salas-Salvado et al (2014), less complete outcomes than the primary source

Sen, *et al.*	Relationships between serum and colon concentrations of carotenoids and fatty acids in randomized dietary intervention trial	Ineligible outcomes
Sondergaard, *et al.*	Effect of dietary intervention and lipid-lowering treatment on brachial vasoreactivity in patients with ischemic heart disease and hypercholesterolemia	Ineligible type of paper
Soto Rodriguez, *et al.*	Benefits of an educational intervention on diet and anthropometric profile of women with one cardiovascular risk factor	Ineligible type of intervention/ control
Sureda, *et al.*	Mediterranean diets supplemented with virgin olive oil and nuts enhance plasmatic antioxidant capabilities and decrease xanthine oxidase activity in people with metabolic syndrome: The PREDIMED study	Ineligible outcomes
Tirosh, *et al.*	Renal function following three distinct weight loss dietary strategies during 2 years of a randomized controlled trial	Ineligible outcomes
Torres-Pena, *et al.*	Mediterranean diet improves endothelial function in patients with diabetes and prediabetes: A report from the CORDIOPREV study	Unusable data
Tripp, *et al.*	A Low-Glycemic, Mediterranean Diet and Lifestyle Modification Program with Targeted Nutraceuticals Reduces Body Weight, Improves Cardiometabolic Variables and Longevity Biomarkers in Overweight Subjects: A 13-Week Observational Trial	Ineligible type of intervention/ control
Trovato, *et al.*	Mediterranean diet and non-alcoholic fatty liver disease: The need of extended and comprehensive interventions	Ineligible study design
Tsaban, *et al.*	Dynamics of intrapericardial and extrapericardial fat tissues during long-term, dietary-induced, moderate weight loss	Ineligible outcomes
Umoh, *et al.*	Markers of systemic exposures to products of intestinal bacteria in a dietary intervention study	Ineligible outcomes
Urpi-Sarda, *et al.*	Virgin olive oil and nuts as key foods of the Mediterranean diet effects on inflammatory biomakers related to atherosclerosis	Ineligible type of paper
Urpi-Sarda, *et al.*	The Mediterranean Diet Pattern and Its Main Components Are Associated with Lower Plasma Concentrations of Tumor Necrosis Factor Receptor 60 in Patients at High Risk for Cardiovascular Disease	Refers to the PREDIMED study, less complete outcomes than the primary sources
Urquiaga, *et al.*	Effect of Mediterranean and Occidental diets, and red wine, on plasma fatty acids in humans. An intervention study	Ineligible outcomes
Urquiaga, *et al.*	Mediterranean diet and red wine protect against oxidative damage in young volunteers	Unusable data
Villarini, *et al.*	Preventing weight gain during adjuvant chemotherapy for breast cancer: a dietary intervention study	Ineligible type of intervention/ control
Wang, *et al.*	Plasma Ceramides, Mediterranean Diet, and Incident Cardiovascular Disease in the PREDIMED Trial (Prevención con Dieta Mediterránea)	Ineligible outcomes
Weber, *et al.*	Effects of Brazilian Cardioprotective Diet Program on risk factors in patients with coronary heart disease: a Brazilian Cardioprotective Diet randomized pilot trial	Ineligible type of intervention/ control
Yubero-Serrano, *et al.*	Postprandial antioxidant effect of the Mediterranean diet supplemented with coenzyme Q10 in elderly men and women	Unusable data
Zambon, *et al.*	Substituting walnuts for monounsaturated fat improves the serum lipid profile of hypercholesterolemic men and women - A randomized crossover trial	Ineligible type of intervention/ control
Zamora-Ros, *et al.*	Mediterranean diet and non enzymatic antioxidant capacity in the PREDIMED study: Evidence for a mechanism of antioxidant tuning	Refers to the same study as Storniolo et al (2017), less complete outcomes than the primary source
Zuniga, *et al.*	Dietary intervention among breast cancer survivors increased adherence to a Mediterranean-style, anti-inflammatory dietary pattern: the Rx for Better Breast Health Randomized Controlled Trial	Ineligible outcomes

Supplementary Table S5. Characteristics of included studies

								Control group			Intervention group			Treatment	
									Male/Female			Male/Female			
Study	Country	Design	Food provided	Population	Duration (weeks)	n	n (I/C)	Mean age (SD)	n	%	Mean age (SD)	n	%	Intervention group	Control group
Almanza et al. 2018 [27]	ES	Parallel RCT	No	Metabolically healthy obese women	12	115	67/48	44.4 (3.3)	0/27	0.0/ 100.0	45.7 (3.5)	0/30	0.0/ 100.0	Education on ↓ energy MD and ↑ PA (≥150 min/ wk of walking)	Advice on cardiometabolic healthy diet and PA
Álvarez-Pérez et al. 2016 [28]	ES	Parallel RCT	Yes	Community dwelling elderly adults with T2D or ≥3 CVD risk factors	52	351	117/117 117	NR	35/82	30.2/ 69.8	NR NR	42/75 42/75	35.9/ 64.1 35.9/ 64.1	1. Education on MD and EVOO 2. Education on MD and nuts	Education on LFD
Ambring et al. 2004 [29]	SE	Cross-over RCT	Yes	Healthy adults	4	22	22/22	43.0 (4.7)	12/10	54.5/ 45.5	43.0 (4.7)	12/10	54.5/ 45.5	Education and meals on MD, ≤2 exercise periods/wk	Education and meals on usual Swedish diet, ≤2 exercise periods/wk
Austel et al. 2015 [30]	DE	Parallel RCT	No	Overweight/ obese adults	12	225	100/ 112	52.6 (10.9)	17/95	15.1/ 84.8	52.4 (8.9)	21/79	21.0/ 79.0	Education on MD	No treatment (waiting list)
Babio et al. 2014 [31]	ES	Parallel RCT	Yes	Community dwelling elderly adults with T2D or ≥3 CVD risk factors	250	5801	1982/ 1885 1934	67.3 (6.3)	777/ 1157	40.2/ 59.8	67.1 (6.2) 66.7 (6.1)	811/ 1171 849/ 1036	40.9/ 59.1 45.0/ 55.0	1. Education on MD and EVOO 2. Education on MD and nuts	Education on LFD
Bajerska et al. 2018 [32]	PL	Parallel RCT	Yes	Centrally obese postmenopausal women	16	144	72/72	60.5 (NR)	0/72	0.0/ 100.0	60.5 (NR)	0/72	0.0/ 100.0	Education and meals on ↓ energy MD, usual PA	Education and meals on ↓ energy Central European diet, usual PA
Bemelmans et al. 2000 [33]	NL	Parallel RCT	Yes	Adults with high cholesterol and ≥2 other CVD risk factors	52	266	103/ 163	54.0 (9.0)	80/83	49.0/ 51.0	55.0 (10.0)	38/65	37.0/ 63.0	Education on MD	Usual care (advice on Dutch dietary guidelines)
Biolato et al. 2019 [35]	IT	Cross-over, non-RCT	No	Non-diabetic adults with NAFLD	16	20	20/14	NR	NR	NR	42.7 (NR)	18/2	90.0/ 10.0	Education on ↓ energy MD and usual PA	Education on ↓ energy LFD and usual PA
Braakhuis et al. 2017 [36]	NZ	Parallel RCT	Yes	Survivors of stage 1-3 breast cancer	24	50	17 16	55.2 (8.3)	0/16	0.0/ 100.0	54.7 (6.2)	0/17	0.0/ 100.0	Education on MD	Education on LFD
Buscemi et al. 2009 [37]	IT	Parallel RCT	No	Healthy females with overweight/obesity	8	20	10/10	38.0 (9.5)	0/10	0.0/ 100.0	39.0 (9.5)	0/10	0.0/ 100.0	Education on ↓ energy MD and usual PA	Education on ↓ energy, very low CHO diet and usual PA
Carruba 2006 [38]	IT	Parallel RCT	No	Healthy females	24	115	58/57	NR	0/57	0/100.0	NR	0/58	0.0/ 100.0	Education on MD	No treatment

Supplementary Table S5 (continued)

								Control group			Intervention group			Treatment	
									Male/Female			Male/Female			
Study	Country	Design	Food provided	Population	Duration (weeks)	n	n (I/C)	Mean age (SD)	n	%	Mean age (SD)	n	%	Intervention group	Control group
Casas et al 2016 [39]	ES	Parallel RCT	Yes	Community dwelling elderly adults with T2D or ≥3 CVD risk factors	260	165	55/55 55	66.3 (6.3)	20/33	39.0/ 61.0	66.7 (6.0) 65.8 (5.6)	23/32 31/23	43.0/ 57.0 57.0/ 43.0	1. Education on MD and EVOO 2. Education on MD and nuts	Education on LFD
Davis et al. 2017 [40]	AU	Parallel RCT	Yes	Adults ≥65 y	24	166	80/72	70.8 (4.7)	33/39	46.0/ 54.0	71.0 (4.9)	33/47	42.0/ 58.0	Education on MD and usual PA	No treatment and usual PA
Davis et al. 2017 [41]	AU	Parallel RCT	Yes	Adults ≥65 y	24	166	85/81	70.9 (4.9)	36/45	44.9/ 55.1	71.0 (4.9)	36/49	42.5/ 57.5	Education on MD and usual PA	No treatment and usual PA
de Lorgeril 1994 [42]	FR	Parallel RCT	Yes	Survivors of a myocardial infarction	260	605	302/ 303	53.5 (10.0)	279/ 24	92.1/ 7.9	53.5 (10.0)	270/32	89.4/ 10.6	Education on MD	No treatment
Duś-Zuchowska et al. 2018 [43]	PL	Parallel RCT	Yes	Women with central obesity and ≥1 MetSyn risk factors	16	144	72/72	60.8 (4.7)	0/72	0.0/ 100.0	60.3 (4.7)	0/72	0.0/ 100.0	Education and meals on ↓ energy MD	Education and meals on ↓ energy Central European diet
Elhayany et al. 2010 [44]	IL	Parallel RCT	No	Overweight patients with T2D	48	259	89/85	56.0 (6.1)	27/28	49.1/ 50.9	57.4 (6.1)	35/28	55.5/ 44.5	Education on MD and advice on ↑ PA (30-45 min ≥3 days/wk)	ADA dietary guidelines+ advice on ↑ PA (30-45 min ≥3 days/wk)
Entwistle et al. 2018 [45]	UK	Parallel pilot RCT	No	Heart and lung transplant recipients	48	41	21/20	59.0 (27.0-65.0)	14/6	70.0/ 30.0	58.0 (33.0-65.0)	15/6	71.0/ 29.0	Education on MD	Education on LFD
Esposito et al. 2003 [50]	IT	Parallel RCT	No	Premenopausal obese women	104	120	60/60	35.0 (5.1)	0/60	0.0/ 100.0	34.2 (4.8)	0/60	0.0/ 100.0	Education on ↓ energy MD and advice on ↑ PA	Advice on healthy eating and general PA advice
Esposito et al. 2004 [49]	IT	Parallel RCT	No	Adults with the MetSyn	104	180	90/90	43.5 (5.9)	50/40	56.0/ 44.0	44.3 (6.4)	49/51	54.0/ 46.0	Education on ↓ energy MD and advice on ↑ PA (≥30 min/ day)	Advice on prudent diet and ↑ PA (≥30 min/ day)
Esposito et al. 2007 [46]	IT	Parallel RCT	No	Women with the MetSyn	104	59	31/28	41.5 (3.9)	0/28	0.0/ 100.0	42.3 (4.5)	0/31	0.0/ 100.0	Education on ↓ energy (if needed) MD and ↑ PA (≥30 min/ day)	Advice on healthy eating and ↑ PA (≥30 min/ day)
Esposito et al. 2009 [47] ‡	IT	Parallel RCT	No	Overweight adults with newly diagnosed T2D	208	215	108/ 107	51.9 (10.7)	52/55	48.5/ 51.5	52.4 (11.2)	54/54	50.0/ 50.0	Education on ↓ energy, ↓CHO MD and advice on ↑ PA (≥30 min/ day)	Education on ↓ energy LFD and advice on ↑ PA (≥30 min/ day)

Supplementary Table S5 (continued)

								Control group			Intervention group			Treatment	
									Male/Female			Male/Female			
**Study**	**Country**	**Design**	**Food provided**	**Population**	**Duration (weeks)**	**n**	**n (I/C)**	**Mean age (SD)**	**n**	**%**	**Mean age (SD)**	**n**	**%**	**Intervention group**	**Control group**
Esposito et al. 2014 [48] ‡	IT	Parallel RCT	No	Overweight adults with newly diagnosed T2D	312	215	108/ 107	51.9 (10.7)	52/55	48.5/ 51.5	52.4 (11.2)	54/54	50.0/ 50.0	Education on ↓ energy, ↓CHO MD and advice on ↑ PA (≥30 min/ day)	Education on ↓ energy LFD and ↑ PA (≥30 min/ day)
Estruch et al. 2006 [52]	ES	Parallel RCT	Yes	Community dwelling elderly adults with T2D or ≥3 CVD risk factors	12	772	257/ 258 257	69.5 (6.1)	109/ 148	42.0/ 58.0	68.6 (6.9) 68.5 (6.2)	102/155 128/130	40.0/ 60.0 50.0/ 50.0	1. Education on MD and EVOO 2. Education on MD and nuts	Education on LFD
Estruch et al. 2018 [53]	ES	Parallel RCT	Yes	Community dwelling elderly adults with T2D or ≥3 CVD risk factors	250	7447	2543/ 2454 2450	67.3 (6.3)	987/ 1463	40.3/ 59.7	67.0 (6.2) 66.7 (6.1)	1050/ 1493 1128/ 1326	41.3/ 58.7 46.0/ 54.0	1. Education on MD and EVOO 2. Education on MD and nuts	Education on LFD
Estruch et al. 2019 [51]	ES	Parallel RCT	Yes	Community dwelling elderly adults with T2D or ≥3 CVD risk factors	250	7447	2543/ 2454 2450	67.3 (6.3)	987/ 1463	40.3/ 59.7	67.0 (6.2) 66.7 (6.1)	1050/ 1493 1128/ 1326	41.3/ 58.7 46.0/ 54.0	1. Education on MD and EVOO 2. Education on MD and nuts	Education on LFD
Fitó et al. 2014 [54]	ES	Parallel RCT	Yes	Community dwelling elderly adults with T2D or ≥3 CVD risk factors	52	930	310/ 310 310	67.6 (6.1)	125/ 186	40.2/ 59.8	66.4 (5.7) 66.2 (6.0)	140/170 143/167	45.3/ 54.7 46.2/ 53.8	1. Education on MD and EVOO 2. Education on MD and nuts	Education on LFD
Fortin et al. 2018 [55]	CA	Parallel RCT	No	Adults with T1D and MetSyn	24	28	14/14	49.8 (11.2)	9/5	64.0/ 36.0	52.1 (9.7)	7/7	50.0/ 50.0	Education on MD and usual PA	Education on LFD and usual PA
Fraser et al. 2008 [56]	IL	Parallel RCT	No	Obese adults with T2D	24	259	85 89	57.0 (5.9)	40/32	55.0/ 45.0	55.2 (6.8)	32/32	50.0/ 50.0	Education on MD and advice on ↑ PA (30 min ≥3 days/wk)	Education on LGI diet and advice on ↑ PA (30 min ≥3 days/wk)
Gepner 2018; Gepner 2019 [57, 58] §	IL	Parallel RCT	Yes	Sedentary adults with abdominal obesity or dyslipidemia	72	278	73/76	49.3 (9.3)	64/12	84.0/ 16.0	47.0 (8.9)	62/11	85.0/ 15.0	Education and meals on ↓ energy, ↓CHO MD	Education and meals on ↓ energy LFD
Hagfors 2003; Sköldstam 2003 [59]	SE	Parallel RCT	Yes	Adults with rheumatoid arthritis	12	51	26/25	59.0 (35.0-75.0)*	5/20	20.0/ 80.0	58.0 (33.0-73.0)*	5/21	19.2/ 80.8	Education and meals on MD	No treatment
Hjerkinn et al. 2006 [60]	NO	Parallel RCT	No	Elderly men with hyperlipidemia	36	563	71/68	NR	68/0	100.0/0. 0	NR	71/0	100.0/ 0.0	Education on ↓ energy (if needed) MD	No treatment

Supplementary Table S5 (continued)

								Control group			Intervention group			Treatment	
									Males/Females			Males/Females			
**Study**	**Country**	**Design**	**Food provided**	**Population**	**Duration (weeks)**	**n**	**n (I/C)**	**Mean age (SD)**	**n**	**%**	**Mean age (SD)**	**n**	**%**	**Intervention group**	**Control group**
Jaacks et al. 2018 [61]	USA	Parallel pilot RCT	Yes	Overweight adults	8	37	11/9	NR	NR	NR	NR	NR	NR	Education and meals on MD, usual PA	No treatment and usual PA
Jennings et al. 2019 [62]	EU (IT, UK, NL, PL, FR)	Parallel RCT	Yes	Elderly free-living adults	52	1294	561/ 567	71.0 (3.9)	260/ 307	45.9/54.1	70.7 (4.0)	243/ 318	43.3/ 56.7	Education on MD	General dietary guidelines
Katsagoni 2018 [63]	GR	Parallel RCT	No	Adults with NAFLD	24	42	21/21	47.0 (42.0-60.0)†	13/8	61.9/38.1	44.0 (41.0-60.0)†	13/8	61.9/ 38.1	Education on ↓ energy MD	Education on ↓ energy diet and general dietary guidelines
Lasa et al. 2014 [64]	ES	Parallel RCT	Yes	Community dwelling elderly adults with T2D or ≥3 CVD risk factors	52	191	74/50<br>67	67.2 (6.8)	32/35	47.8 /52.2	67.4 (6.3)<br>67.1 (4.8)	29/45<br>16/34	39.2/ 60.8<br>32.0/ 68.0	1. Education on MD and EVOO<br>2. Education on MD and nuts	Education on LFD
Lee et al. 2015 [65]	AU	Cross-over RCT	No	Healthy women	10 days	24	24/24	25.6 (5.1)	0/24	0.0/ 100.0	25.6 (5.1)	0/24	0.0/ 100.0	Education on MD	No treatment
Maijo et al. 2018 [66]	UK	Parallel RCT	Yes	Elderly free-living adults	52	122	61/61	70.6 (3.8)	23/38	37.0/ 63.0	70.0 (4.2)	26/35	41.8/ 58.2	Education on MD	General dietary guidelines
Maiorino et al. 2016 [67]	IT	Parallel RCT	No	Adults with diagnosed T2D	52	215	108/ 107	51.9 (10.7)	52/55	48.6/ 51.4	52.4 (11.2)	54/54	50.0/ 50.0	Education on MD and advice on ↑ PA	Education on LFD and advice on ↑ PA
Mayr et al. 2018 [69]	AU	Parallel pilot RCT	Yes	Adults with CHD	24	65	27/29	61.8 (9.9)	26/3	89.7/ 10.3	62.7 (7.7)	21/6	77.8/ 22.2	Education on MD	Education on LFD
Mayr et al. 2019 [68]	AU	Parallel pilot RCT	Yes	Adults with CHD	24	73	34/31	61.8 (9.5)	27/4	87.1/ 12.9	61.8 (9.2)	27/7	79.4/ 20.6	Education on MD	Education on LFD
McManus 2001 [70]	USA	Parallel pilot RCT	No	Overweight adults	72	101	50/51	44.0 (10.0)	4/47	8.0/ 92.0	44.0 (10.0)	6/44	12.0/ 88.0	Education on ↓ energy MD	Education on ↓ energy LFD
Meir et al. 2019 [71]	IL	Parallel RCT	Yes	Adults with abdominal obesity or dyslipidemia	24	294	98/98	51.1 (10.6)	86/12	88.0/ 12.0	51.7 (10.4)	86/12	88.0/ 12.0	Education on ↓ energy MD and advice on ↑ PA	No diet treatment and advice on ↑ PA
Mezzano et al. 2001 [72]	CL	Parallel RCT	Yes	Healthy university students	4	42	21/21	22.6 (4.5)	21/0	100.0/ 0.0	21.2 (1.7)	21/0	100.0/ 0.0	MD meals	Western, HFD meals
Michielsen et al. 2019 [73]	NL	Parallel RCT	Yes	Adults at risk of MetSyn	8	30	14/16	51.4 (7.8)	8/8	50.0/ 50.0	57.4 (5.1)	4/10	28.6/ 71.4	Education and meals on MD	Education and meals on high SFA diet

Supplementary Table S5 (continued)

								Control group			Intervention group			Treatment	
									Male/Female			Male/Female			
Study	Country	Design	Food provided	Population	Duration (weeks)	n	n (I/C)	Mean age (SD)	n	%	Mean age (SD)	n	%	Intervention group	Control group
Misciagna 2017 [74]	IT	Parallel RCT	No	Adults with NAFLD	24	98	50/48	NR	38/10	79.2/ 20.8	NR	34/16	68.0/ 32.0	Education on LGI MD	Healthy eating guidelines
Ortner-Hadžiabdić et al. 2016 [75]	HR	Parallel RCT	Yes	Obese adults	52	84	40/44	49.0 (12.1)	13/48	21.3/ 78.7	46.2 (12.·7)	19/44	30.2/ 69.8	Education and supervision on ↓ energy MD and advice on ↑ PA (≥30 min/ day)	Education and supervision on ↓ energy LFD and advice on ↑ PA (≥30 min/ day)
Osella et al. 2018 [76]	IT	Parallel RCT	No	Adults with MetSyn	24	163	51/55	57.5 (10.7)	29/26	52.7/ 47.3	59.4 (10.4)	33/18	64.7/ 35.3	Education on MD and usual PA	Education on LGI diet and usual PA
Paniagua et al. 2007 [77]	ES	Cross-over RCT	Yes	Insulin resistant offspring of adults with T2D	4	11	11 11	62.0 (9.4)	4/7	36.4/ 63.6	62.0 (9.4)	4/7	36.4/ 63.6	HFD, enriched in MUFA diet (MD) and usual PA	LF, high CHO diet and usual PA
Papadaki & Scott 2005 [80]‡	UK	Non-RCT	No	Healthy females	24	72	53/19	40.9 (6.9)	0/19	0.0/ 100.0	40.3 (7.2)	0/53	0.0/ 100.0	Education and tailored feedback on the MD	General healthy eating information and minimally tailored feedback
Papadaki & Scott 2008 [81] ‡	UK	Non-RCT	No	Healthy females	36	72	53/19	40.9 (6.9)	0/19	0.0/ 100.0	40.3 (7.2)	0/53	0.0/ 100.0	Education and tailored feedback on the MD	General healthy eating information and minimally tailored feedback
Papadaki et al. 2017; 2019 [78, 79]	ES	Parallel RCT	Yes	Community dwelling elderly adults with T2D or ≥3 CVD risk factors	353	7403	2527/ 2444 2432	67.3 (6.3)	977/ 1455	40.2/ 59.8	67.0 (6.2) 66.7 (6.1)	1043/ 1484 1125/ 1319	41.3/ 58.7 46.0/ 54.0	1. Education on MD and EVOO 2. Education on MD and nuts	Education on LFD
Papandreou et al. 2012 [82]	GR	Parallel RCT	No	Obese adults with obstructive sleep apnoea	24	40	20/20	45.8 (14.2)	17/3	85.0/ 15.0	52.5 (10.5)	17/3	85.0/ 15.0	Education on ↓ energy MD and advice on ↑ PA (≥30 min/ day)	Education on ↓ energy prudent diet and advice on ↑ PA (≥30 min/ day)
Parcina et al. 2015 [83]	DE	Parallel RCT	Yes	Healthy males	2	39	14/13	29.1 (5.8)	13/0	100.0/ 0.0	31.9 (6.3)	14/0	100.0/ 0.0	MD regime and usual PA	German cooking style regime and usual PA
Properzi et al. 2018 [84]	AU	Parallel RCT	Yes	Adults with NAFLD	12	51	26/25	53.0 (9.1)	11/14	44.0/56.0	51.0 (13.4)	15/11	57.7/ 42.3	Education on MD and usual PA	Education on LFD and usual PA

Supplementary Table S5 (continued)

								Control group			Intervention group			Treatment	
									Male/Female			Male/Female			
**Study**	**Country**	**Design**	**Food provided**	**Population**	**Duration (weeks)**	**n**	**n (I/C)**	**Mean age (SD)**	**n**	**%**	**Mean age (SD)**	**n**	**%**	**Intervention group**	**Control group**
Richard 2011; Richard et 2013 [85, 86]	CA	Non- RCT	Yes	Men with MetSyn	5	26	26/26	49.4 (11.6)	26/0	100.0/0.0	49.4 (11.6)	26/0	100.0/ 0.0	MD meals and usual PA	North American diet meals and usual PA
Rogerson 2018 [87]	UK	Non-RCT	Yes	Sedentary adults	4	24	12/12	26.0 (4.3)	4/8	33.3/66.7	25.0 (2.6)	2/10	16.7/ 83.3	Education on MD	Education on a vegan diet
Ryan et al. 2013 [88]	AU	Cross-over RCT	Yes	Adults with NAFLD but without T2D	6	12	12/12	55.0 (14.0)	6/6	50.0/50.0	55.0 (14.0)	6/6	50.0/ 50.0	Education and meals on MD, usual PA	Education and meals on LF, high CHO diet, usual PA
Salas-Salvado et al. 2014 [89]	ES	Parallel RCT	Yes	Community dwelling elderly adults with T2D or ≥3 CVD risk factors	213	3541	1154/ 1240 1147	67.2 (6.1)	401/ 746	35.0/65.0	66.5 (6.0) 66.2 (6.0)	439/715 506/734	38.0/ 62.0 40.8/ 59.2	1. Education on MD and EVOO 2. Education on MD and nuts	Education on LFD
Sala-Vila et al. 2014 [90]	ES	Parallel RCT	Yes	Community dwelling elderly adults with T2D or ≥3 CVD risk factors	125	164	57/46 61	66.0 (9.6)	23/38	38.0/62.0	67.0 (6.5) 66.0 (10.1)	28/29 25/21	49.0/ 51.0 54.0/ 46.0	1. Education on MD and EVOO 2. Education on MD and nuts	Education on LFD
Shai et al. 2008; Ben-Avraham 2009 [34, 91]	IL	Parallel RCT	No	Moderately obese adults	104	322	109 104	51.0 (7.0)	89/15	86.0/14.0	53.0 (6.0)	89/20	82.0/ 18.0	Education on ↓ energy MD	Education on ↓ energy LFD
Shai et al. 2010 [92]	IL	Parallel RCT	No	Moderately obese adults	104	140	55 49	NR	NR	NR	NR	NR	NR	Education on ↓ energy MD	Education on ↓ energy LFD
Singh et al. 2002 [93]	IN	Parallel RCT	No	Patients with angina pectoris, myocardial infarction, or risk factors for CAD	104	1000	499/ 501	48.0 (9.0)	NR	NR	49.0 (10.0)	NR	NR	Education on Indo-MD and advice on ↑ PA (brisk walking for ≥3-4 km or jogging ≥10-15 min/ day)	Education on the NCEP-1 diet and advice on ↑ PA (brisk walking for ≥3-4 km or jogging ≥10-15 min/ day)
Singh et al. 2017 [94]	IN	Parallel RCT	No	Patients with acute coronary syndrome	104	406	204/ 202	52.0 (8.3)	185/ 17	92.0/ 8.0	50.5 (9.3)	180/24	88.0/ 12.0	Education on Indo-MD and advice on regular PA	Education on the NCEP-1 diet and advice on regular PA
Skouroliakou et al. 2018 [96]	GR	Parallel RCT	No	Breast cancer survivors	24	70	26/24	NR	0/24	0.0/ 100.0	NR	0/26	0.0/ 100.0	Personalised education on MD and PA cancer guidelines	Updated cancer prevention guidelines and PA cancer guidelines

Supplementary Table S5 (continued)

Study	Country	Design	Food provided	Population	Duration (weeks)	n	n (I/C)	Control group Male/Female Mean age (SD)	n	%	Intervention group Male/Female Mean age (SD)	n	%	Treatment Intervention group	Control group
Sofi et al. 2018 [97]	IT	Cross-over RCT	No	Clinically healthy omnivorous adults	12	118	58/60	49.5 (24.0-70.0)*	11/49	18.3/ 81.7	52.0 (21.0-75.0)*	15/43	25.9/ 74.1	Education on ↓ energy MD and usual PA	Education on ↓ energy vegetarian diet and usual PA
Sola et al. 2011 [98]	ES	Parallel RCT	Yes	Community dwelling elderly adults with T2D or ≥3 CVD risk factors	12	551	181/ 193 177	69.7 (6.3)	72/ 105	40.7/ 59.3	69.3 (6.2) 68.4 (5.9)	74/107 97/96	40.9/ 59.1 50.3/ 49.7	1. Education on MD and EVOO 2. Education on MD and nuts	Education on LFD
Stachowska et al. 2006 [99]	PL	Parallel RCT	No	Kidney transplant patients	24	37	21/16	46.0 (9.5)	10/6	62.5/ 37.5	41.0 (12.5)	15/6	71.4/ 28.6	Education on isocaloric MD	Education on isocaloric LFD
Storniolo et al. 2017 [100]	ES	Parallel RCT	Yes	Community dwelling hypertensive women with T2D or ≥3 CVD risk factors	52	90	30/30 30	68.1 (5.2)	0/30	0.0/10 0.0	69.1 (5.5) 68.7 (5.2)	0/30 0/30	0.0/ 100.0 0.0/ 100.0	1. Education on MD and EVOO 2. Education on MD and nuts	Education on LFD
Thomazella et al. 2011 [101]	BR	Non-RCT	Yes	Males with stable CAD	12	40	21/19	54.6 (5.0)	19/0	100.0/ 0.0	55.0 (4.6)	21/0	100.0/ 0.0	Personalised education on MD and usual PA	Personalised education on the NCEP diet and usual PA
Timar et al. 2013 [102]	RO	Non-RCT	No	Overweight adults with T2D	52	223	68 88	NR	45/43	51.1/4 8.9	NR	32/36	47.1/ 52.9	Education on energy-controlled MD and advice on ↑ PA (150 min/ wk over ≥3 days)	Education on energy-controll ed standard T2D diet and advice on ↑ PA (150 min/ wk over ≥3 days)
Toledo et al. 2013 [103]	ES	Parallel RCT	Yes	Community dwelling elderly adults with T2D or ≥3 CVD risk factors	208	7158	2441/ 2367 2350	67.3 (6.3)	948/ 1402	40.3/5 9.7	66.9 (6.2) 66.6 (6.1)	1017/ 1424 1092/ 1275	41.7/ 58.3 46.1/ 53.9	1. Education on MD and EVOO 2. Education on MD and nuts	Education on LFD
Toledo et al. 2015 [104]	ES	Parallel RCT	Yes	Community dwelling women with T2D or ≥3 CVD risk factors and no history of breast cancer	250	4152	1476/ 1285 1391	68.1 (6.0)	0/ 1391	0.0/ 100.0	67.6 (5.8) 67.4 (5.6)	0/1476 0/1285	0.0/ 100.0 0.0/ 100.0	1. Education on MD and EVOO 2. Education on MD and nuts	Education on LFD
Troseid et al. 2009 [105]	NO	Parallel RCT	No	Elderly men with hyperlipidemia	36	563	281/ 282	NR	282/0	100.0/ 0.0	NR	281/0	100.0/ 0.0	Education on ↓ energy (if needed) MD	No treatment

Supplementary Table S5 (continued)

Study	Country	Design	Food provided	Population	Duration (weeks)	n	n (I/C)	Control group Male/Female Mean age (SD)	n	%	Intervention group Male/Female Mean age (SD)	n	%	Treatment Intervention group	Control group
Tutino et al. 2018 [106]	IT	Parallel RCT	No	Adults with NAFLD	12	142	21/20	52.1 (9.5)	NR	NR	55.5 (10.4)	NR	NR	Education on LGI MD	Healthy eating guidelines
Vincent-Baudry et al. 2005; Vincent 2004 [107, 111]§	FR	Parallel RCT	Yes	Adults at moderate CVD risk	12	212	88/81	51.6 (10.3)	NR	NR	50.8 (10.8)	NR	NR	Education on MD and usual PA	Education on ↓ energy (if needed) LFD and usual PA
Wade et al. 2018 [108]	AU	Cross-over RCT	Yes	Adults at CVD risk	8	41	20/21	59.6 (7.6)	7/14	33.3/ 66.7	60.8 (6.3)	6/14	30.0/ 70.0	Education on MD supplemented with dairy products and usual PA	Education on LFD and usual PA
Wade et al. 2019 [109]	AU	Cross-over RCT	Yes	Adults at CVD risk	8	33	33/33	61.6 (5.7)	4/14	22.2/ 77.8	60.2 (8.7)	6/9	40.0/ 60.0	Education on MD supplemented with lean pork and usual PA	Education on LFD and usual PA
Wardle et al. 2000 [110]	UK	Parallel RCT	Yes	Adults with elevated serum cholesterol levels	12	176	61/ 59	52.0 (11.0)	34/25	58.0/ 42.0	54.0 (11.0)	27/34	44.0/ 56.0	Education on MD	Education on LFD

ADA, American Diabetes Association; AU, Australia; BMI, body mass index; BR, Brazil;C, control; CA, Canada; CAD, coronary artery disease; CHD, coronary heart disease; CHO, carbohydrates; CL, Chile; CVD, cardiovascular disease; DE, Germany; ES, Spain; EVOO, extra-virgin olive oil; FR, France; FU, follow-up; GR, Greece; HF, heart failure; HFD, high-fat diet; HR, Croatia; I, intervention; IL, Israel; IN, India; IT, Italy; LFD, low-fat diet; LGI, low glycemic index; MD, Mediterranean Diet; MetSyn, metabolic syndrome; MUFA, monounsaturated fatty acids; NAFLD, non-alcoholic fatty liver disease; NCEP-1, National Cholesterol Education Programme stage 1; NL, Netherlands; NO, Norway; NR, not reported; NZ, New Zealand; PA, Physical Activity; PL, Poland; RCT, randomised controlled trial; RO, Romania; SD, standard deviation; SE, Sweden; SFA, saturated fatty acids; T1D, type 1 diabetes; T2D, type 2 diabetes; UK, United Kingdom; USA, United States of America.

*Mean (range) reported. †Median (interquartile range) reported. ‡Four papers reported separately on the post-intervention [47, 80], and extended follow-up [48, 81] of the same trials. All these papers were included independently in the qualitative synthesis but only the papers reporting on the post-intervention results [47, 80] were included in the pooled analysis. §Two trials reported the study characteristics in two separate publications [57, 58, 107, 111], which were merged for the purposes of this review.

Supplementary Table S6. Foods provided to the intervention group, for papers reporting on studies that supplemented the dietary advice intervention with foods

Study	Food provision
Álvarez-Pérez et al. 2016 [28]	Intervention group 1: Free provision of EVOO (1 L/wk)<br>Intervention group 2: Free provision of nuts [sachets of walnuts (15 g/d), hazelnuts (7.5 g/d), and almonds (7.5 g/d)<br><br>Aim of food provision was to improve adherence to the intervention; no energy restriction was suggested and participants were advised on the desired frequency of intake of specific foods.
Ambring et al. 2004 [29]	Participants were provided with 60% of their daily caloric needs, including one cooked meal/day, and sterol esters (2 g/day) as an ingredient in a margarine.<br><br>Low-fat products were chosen by the subjects themselves for the remaining daily energy intake (40%).
Babio et al. 2014 [31]	Intervention group 1: Free provision of EVOO (1 L/wk)<br>Intervention group 2: Free provision of nuts [sachets of walnuts (15 g/d), hazelnuts (7.5 g/d), and almonds (7.5 g/d)<br><br>Aim of food provision was to improve adherence to the intervention; no energy restriction was suggested and participants were advised on the desired frequency of intake of specific foods.
Bajerska et al. 2018 [32]	Participants were provided with packaged main meals (covering ~35% of their daily energy needs).<br><br>Aim of food provision was to optimise control for energy and macronutrient intake. Participants were advised on how to prepare remaining meals at home.
Bemelmans et al. 2000 [33]	Participants were provided with a polyunsaturated fatty acid-rich margarine.<br><br>No energy restriction was suggested and participants were advised on the desired frequency of intake of specific foods.
Braakhuis et al. 2017 [36]	Participants were provided with olive leaf extract.<br><br>No energy restriction was suggested.
Casas et al 2016 [39]	Intervention group 1: Free provision of EVOO (1 L/wk)<br>Intervention group 2: Free provision of nuts [sachets of walnuts (15 g/d), hazelnuts (7.5 g/d), and almonds (7.5 g/d)<br><br>Aim of food provision was to improve adherence to the intervention; no energy restriction was suggested and participants were advised on the desired frequency of intake of specific foods.
Davis et al. 2017 [40]	Participants were provided with all of the recommended EVOO, nuts (50% walnuts, 25% almonds and hazelnuts), Greek yogurt, and canned legumes. Canned tuna was given to provide 30% of the fish requirements.<br><br>Aim of food provision was to improve adherence; participants were advised on the desired frequency of intake of specific foods.
Davis et al. 2017 [41]	Participants were provided with all of the recommended EVOO, nuts (50% walnuts, 25% almonds and hazelnuts), Greek yogurt, and canned legumes. Canned tuna was given to provide 30% of the fish requirements.<br><br>Aim of food provision was to improve adherence; participants were advised on the desired frequency of intake of specific foods.
de Lorgeril et al. 1994 [42]	Participants were provided with canola oil-based margarine, high in n-3 fatty acids, to replace butter and cream.<br><br>Aim of food provision was to improve the MD's acceptability (as participants might not accept olive oil as the only source of fat in the diet). Participants were advised on the desired frequency of intake of specific foods.
Duś-Żuchowska et al. 2018 [43]	Participants were provided with pre-portioned main meals (covering ~35% of their daily caloric needs) for the whole period of the study.<br><br>Aim of food provision was to According to reduce the overall number of food items at home and decrease high-fat food choices (by using a home-delivery service). Energy restriction was suggested. Participants were advised on how to prepare remaining meals at home.
Estruch et al. 2006 [52]	Intervention group 1: Free provision of EVOO (1 L/wk)<br>Intervention group 2: Free provision of nuts [sachets of walnuts (15 g/d), hazelnuts (7.5 g/d), and almonds (7.5 g/d)<br><br>Aim of food provision was to improve adherence to the intervention; no energy restriction was suggested and participants were advised on the desired frequency of intake of specific foods.

Estruch et al. 2018 [53]	Intervention group 1: Free provision of EVOO (1 L/wk)<br>Intervention group 2: Free provision of nuts [sachets of walnuts (15 g/d), hazelnuts (7.5 g/d), and almonds (7.5 g/d)<br><br>Aim of food provision was to improve adherence to the intervention; no energy restriction was suggested and participants were advised on the desired frequency of intake of specific foods.
Estruch et al. 2019 [51]	Intervention group 1: Free provision of EVOO (1 L/wk)<br>Intervention group 2: Free provision of nuts [sachets of walnuts (15 g/d), hazelnuts (7.5 g/d), and almonds (7.5 g/d)<br><br>Aim of food provision was to improve adherence to the intervention; no energy restriction was suggested and participants were advised on the desired frequency of intake of specific foods.
Fitó et al. 2014 [54]	Intervention group 1: Free provision of EVOO (1 L/wk)<br>Intervention group 2: Free provision of nuts [sachets of walnuts (15 g/d), hazelnuts (7.5 g/d), and almonds (7.5 g/d)<br><br>Aim of food provision was to improve adherence to the intervention; no energy restriction was suggested and participants were advised on the desired frequency of intake of specific foods.
Gepner et al. 2018; Gepner et al. 2019 [57, 58]	Participants were provided with walnuts (28 g/d, starting from the third month). Lunch was provided on site.
Hagfors et al. 2003;<br>Sköldstam et al. 2003 [59, 95]	Participants were provided with lunch and dinner (for the first three weeks) and with frozen vegetables, tea, olive oil, canola oil and liquid and half-fat margarines based on canola oil (for the remaining of the study).<br><br>Aim of food provision was to promote compliance; participants were advised on how to prepare meals at home.
Jaacks et al. 2018 [61]	Participants were provided with three meals with beverages and two snacks per day (for half the study's duration).<br><br>Aim of food provision was to provide daily energy intake for weight maintenance. Participants were provided with information on the MD's composition
Jennings et al. 2019 [62]	Participants were provided with commercially available foods, including whole-grain pasta, olive oil, high-MUFA and high-PUFA margarine, and low-fat, low-salt cheese in all centers and frozen vegetable soup (in Italy only).<br><br>Aim of food provision was to facilitate dietary compliance and help meet dietary guidelines. No energy restriction was suggested and participants were advised on the desired frequency of intake of specific foods.
Lasa et al. 2014 [64]	Intervention group 1: Free provision of EVOO (1 L/wk)<br>Intervention group 2: Free provision of nuts [sachets of walnuts (15 g/d), hazelnuts (7.5 g/d), and almonds (7.5 g/d)<br><br>Aim of food provision was to improve adherence to the intervention; no energy restriction was suggested and participants were advised on the desired frequency of intake of specific foods.
Maijo et al. 2018 [66]	Participants were provided with commercially available foods, including whole-grain pasta, olive oil, high-MUFA and high-PUFA margarine, and low-fat, low-salt cheese in all centers and frozen vegetable soup (in Italy only).<br><br>Aim of food provision was to facilitate dietary compliance and help meet dietary guidelines. No energy restriction was suggested and participants were advised on the desired frequency of intake of specific foods.
Mayr et al. 2018 [69]	Participants were provided with EVOO (60-80 mL/d), nuts (almonds, walnuts and hazelnuts, 30 g/d) and samples of tinned tuna and salmon, canned legumes and Greek yoghurt.<br><br>Aim of food provision was to facilitate dietary compliance and encourage intake of staple Mediterranean foods that participants might not have been familiar with. No energy restriction was suggested and participants were advised on the desired frequency of intake of specific foods.
Mayr et al. 2019 [68]	Participants were provided with EVOO (60-80 mL/d), nuts (almonds, walnuts and hazelnuts, 30 g/d) and samples of tinned tuna and salmon, canned legumes and Greek yoghurt.<br><br>Aim of food provision was to facilitate dietary compliance and encourage intake of staple Mediterranean foods that participants might not have been familiar with. No energy restriction was suggested and participants were advised on the desired frequency of intake of specific foods.
Meir et al. 2019 [71]	Participants were provided with walnuts (28 g/d).<br><br>Energy restriction was suggested and participants were advised on the desired frequency of intake of specific foods.
Mezzano et al. 2001 [72]	Participants were provided with personalised boxes of lunch and dinner (specific indications for breakfast and snacks were provided).<br><br>Aim of food provision was to optimise control for energy and macronutrient intake.

Michielsen et al. 2019 [73]	Participants were provided with 90% of their energy needs (no details provided).
Ortner- Hadžiabdić et al. 2016 [75]	Participants were provided with EVOO. Breakfast and lunch were consumed each day on site, which served as an educational measure for the amount and type of food participants should consume at home.<br><br>Energy restriction was suggested and participants were advised on the desired frequency of intake of specific foods.
Paniagua et al. 2007 [77]	Participants were provided with breakfast comprised of 200 ml skim milk, 50 g bread and 27 g olive oil.
Papadaki et al. 2017; 2019 [78, 79]	Intervention group 1: Free provision of EVOO (1 L/wk)<br>Intervention group 2: Free provision of nuts [sachets of walnuts (15 g/d), hazelnuts (7.5 g/d), and almonds (7.5 g/d)<br><br>Aim of food provision was to improve adherence to the intervention; no energy restriction was suggested and participants were advised on the desired frequency of intake of specific foods.
Parcina et al. 2015 [83]	Participants were provided, on each day, with three freshly prepared meals from high quality foods.<br><br>Aim of food provision was to assure compliance (only calorie-free drinks were allowed outside these meals).
Properzi et al. 2018 [84]	Participants were provided, at each 4-weekly visit, with 750 g of nuts (almonds or walnuts) and 750 mL of olive oil.<br><br>Aim of the food provision was to minimise financial disadvantage to participants consuming core foods in the MD. Participants were advised on the desired frequency of intake of specific foods.
Richard et al. 2011; Richard et al. 2013 [85, 86]	Participants were provided with all meals, foods and beverages (including alcohol).<br><br>Aim of food provision was to optimise control for energy and macronutrient intake.
Rogerson et al. 2018 [87]	Participants were provided with food items to assist adherence (no details provided).<br><br>Participants were advised on the desired frequency of intake of specific foods. No energy restriction was suggested.
Ryan et al. 2013 [88]	Participants were provided with the majority of foods (70%) on the intervention diet for free: olives, dried fruit, nuts, Greek yoghurt, fish, and EVOO.<br><br>To facilitate compliance, participants were provided with precooked meals. No energy restriction was suggested and participants were advised to records their intake and discard leftovers.
Salas-Salvado et al. 2014 [89]	Intervention group 1: Free provision of EVOO (1 L/wk)<br>Intervention group 2: Free provision of nuts [sachets of walnuts (15 g/d), hazelnuts (7.5 g/d), and almonds (7.5 g/d)<br><br>Aim of food provision was to improve adherence to the intervention; no energy restriction was suggested and participants were advised on the desired frequency of intake of specific foods.
Sala-Vila et al. 2014 [90]	Intervention group 1: Free provision of EVOO (1 L/wk)<br>Intervention group 2: Free provision of nuts [sachets of walnuts (15 g/d), hazelnuts (7.5 g/d), and almonds (7.5 g/d)<br><br>Aim of food provision was to improve adherence to the intervention; no energy restriction was suggested and participants were advised on the desired frequency of intake of specific foods.
Sola et al. 2011 [98]	Intervention group 1: Free provision of EVOO (1 L/wk)<br>Intervention group 2: Free provision of nuts [sachets of walnuts (15 g/d), hazelnuts (7.5 g/d), and almonds (7.5 g/d)<br><br>Aim of food provision was to improve adherence to the intervention; no energy restriction was suggested and participants were advised on the desired frequency of intake of specific foods.
Storniolo et al. 2017 [100]	Intervention group 1: Free provision of EVOO (1 L/wk)<br>Intervention group 2: Free provision of nuts [sachets of walnuts (15 g/d), hazelnuts (7.5 g/d), and almonds (7.5 g/d)<br><br>Aim of food provision was to improve adherence to the intervention; no energy restriction was suggested and participants were advised on the desired frequency of intake of specific foods.
Thomazella et al. 2011 [101]	Participants were provided with mixed plain nuts (Brazil nuts, almonds, and walnuts, 10 g/day), cabernet sauvignon wine (250 ml/day), and EVOO (15 ml, amber flasks).<br><br>Aim of food provision was to improve adherence. Participants were advised on the desired frequency of intake of specific foods.
Toledo et al. 2013 [103]	Intervention group 1: Free provision of EVOO (1 L/wk)<br>Intervention group 2: Free provision of nuts [sachets of walnuts (15 g/d), hazelnuts (7.5 g/d), and almonds (7.5 g/d)<br><br>Aim of food provision was to improve adherence to the intervention; no energy restriction was suggested and participants were advised on the desired frequency of intake of specific

	foods.
Toledo et al. 2015 [104]	Intervention group 1: Free provision of EVOO (1 L/wk)<br>Intervention group 2: Free provision of nuts [sachets of walnuts (15 g/d), hazelnuts (7.5 g/d), and almonds (7.5 g/d)<br><br>Aim of food provision was to improve adherence to the intervention; no energy restriction was suggested and participants were advised on the desired frequency of intake of specific foods.
Vincent-Baudry et al. 2005; Vincent et al. 2004 [107, 111]	Participants were provided with tomato paste, olive oil and soluble fibre-enriched pasta.<br><br>Participants were advised on the desired frequency of intake of specific foods.
Wade et al. 2018 [108]	Participants were provided with Greek yogurt, almonds, walnuts and hazelnuts, EVOO, regular-fat and reduced-fat cheese slices, chickpeas, cannellini beans, red kidney beans, 4-bean mix, and lentils, and canned tuna and salmon.<br><br>Aim of food provision was to assist with dietary adherence. Participants were advised on the desired frequency of intake of specific foods.
Wade et al. 2019 [109]	Participants were provided each week with 375mL EVOO, 250g of fresh, lean pork, 150g raw, unsalted almonds, walnuts and hazelnuts; 225g (net weight) of canned chickpeas, red kidney beans, 4-bean mix and lentils; 95g of canned tuna and 95g of canned salmon.<br><br>Aim of food provision was to improve adherence to the intervention; no energy restriction was suggested and participants were advised on the desired frequency of intake of specific foods.
Wardle et al. 2000 [110]	Participants were provided with free-spreading fats and oils that were high in monounsaturated fat.<br><br>Aim of food provision was to encourage compliance; participants were advised to substitute predominantly monounsaturated fats for saturated fats.

EVOO, extra-virgin olive oil; MD, Mediterranean Diet; MUFA, monounsaturated fatty acids; PUFA, polyunsaturated fatty acids.

Supplementary Table S7. Summary of the findings on anthropometric and blood pressure markers (between-group differences) from the papers not included in the pooled analysis

	Body weight	Body mass index	Waist circumference	Systolic blood pressure	Diastolic blood pressure
Carruba et al. 2006 [38]	-1.3 vs. -0.6 kg (level of strength of evidence not reported)	-	-	-	-
Esposito et al. 2014 [48]	+0.4 kg [CI -0.1 to 0.7]	-	-	-1.8 mm Hg [95% CI -4.5, 1.0]	-1.5 mm Hg [95% CI -4 to 1.9]
Katsagoni et al. 2018 [63]	-2.7 kg [CI -6.1 to 0.68]	-0.95 kg/m^2 [CI -0.92 to -0.99]	-	-	-
Toledo et al. 2013 [103]	-	-	-	MD (EVOO) vs. CG: 0.39 mm Hg [CI -0.48 to 1.26]; P=0.380<br>MD (nuts) vs. CG: -0.72 mm Hg [CI -1.58 to 0.13]; P=0.100	MD (EVOO) vs. CG: -1.53 mm Hg [CI -2.01 to -1.04]; P<0.001<br>MD (nuts) vs. CG: -0.65 mm Hg [CI -1.15 to -0.15]; P=0.010
Troseid et al. 2009 [105]	-	-0.3 vs. 0.1 kg/m^2, P=0.021	+1 vs. +3 cm, P=0.061	-	-
Vincent-Baudry et al. 2005 [107]	-1.5 vs. -1.2 kg (level of strength of evidence not reported)	-	-	-	-

CG, control group; CI, confidence intervals; EVOO, extra virgin olive oil; MD, Mediterranean diet.

Summary of findings: The MD, as compared to a control condition, showed a protective effect for body weight in 0/4 papers, for body mass index in 2/2 papers, for waist circumference in 0/1 paper, for systolic blood pressure in 0/3 comparisons and for diastolic blood pressure in 2/3 comparisons.

Supplementary Table S8. Summary of the findings on biochemical and markers of insulin resistance (between-group differences) from the papers (and/or outcomes) not included in the pooled analysis

	Glucose	Insulin	HOMA-IR	HbA1c	TC	LDL	HDL
Austel et al. 2015 [30]	0.17 vs. -0.90 mg/dL, P>0.005	-	-	-	-12.6 vs. 0.8 mg/dL, P<0.001	-7.18 vs. 1.14 mg/dL, P<0.001	-1.85 vs. 0.02 mg/dL, P>0.050
Casas et al. 2016 [39]	-	-	-	MD (EVOO) vs. CG: -0.4 mg/dL [CI -0.95 to 0.15]; P=0.159 MD (nuts) vs. CG: -0.30 mg/dL [CI -0.85 to 0.25]; P=0.290	-	-	-
Esposito et al. 2014 [48]	-10 mg/dL [CI -25 to 5]	-	-	-	-4 mg/dL [CI -10 to 2]	-	+4.7 mg/dL [CI 0.2 to 9.1]
Gepner et al. 2018 [58]	-	-	-	-0.04% [CI -0.17 to 0.09]; P=0.532	-	-	-
Katsagoni et al. 2018 [63]	-	-1 pmol/L [CI -0.7 to 1.4]	-1.1 [CI -0.74 to 1.5]	-	-	-	1.1 mmol/L [CI -0.9 to 1.2]
Papadaki & Scott 2008 [81]	-	-	-	-	0.17 mmol/L [CI 0.02 to 0.32]; P=0.010	-0.05 mmol/L [CI -0.23 to 0.13]; P=0.450	0.20 mmol/L [CI 0.13 to 0.27]; P<0.001
Parcina et al. 2015 [83]	-	-	-	-0.02% [CI -0.08 to 0.04]; P=0.548	-	-	-
Properzi et al. 2018 [84]	-	-	-	0.00 [CI -0.14 to 0.14]; P=1.000	-	-	-
Sofi et al. 2018 [97]	-	-	0.0 [CI -0.06 to 0.06]; P=1.000	-	-	-	-

	TG	non-HDL	TC:HDL	ApoB	ALT	GGT
Austel et al. 2015 [30]	-14.76 vs. 12.54mg/dL, P<0.010	-	-	-	-	-
de Lorgeril et al. 1994 [42]	-	-	-	-1 g/L [CI -1.57 to -0.43]; P=0.001	-	-
Entwistle et al. 2018 [45]	-9% [CI -20 to 4] vs. -21% [CI -33 to -7]	-	-	-	-	-
Esposito et al. 2014 [48]	-12 mg/dL [CI -30 to 6]	-	-	-	-	-
Fortin et al. 2018 [55]	-	-	-	-0.14 g/L [CI -0.27 to -0.01]; P=0.04	-	-
Katsagoni et al. 2018 [63]	-	-1.5 mmol/L [CI -2.2 to -1.0]	-	-	-0.79 UI/L [CI -0.57 to 1.1]	-0.79 UI/L [CI -0.54 to 1.15]
Michielsen et al. 2019 [73]	-	-	-	-0.14 g/L [CI -0.22 to -0.06]; P=0.002	-	-
Papadaki & Scott. 2005 [80]	-	-	-0.33 [CI -0.53 to -0.14]; P<0.001	-	-	-
Papadaki & Scott. 2008 [81]	0.08 mmol/L [CI -0.01 to 0.17]; P=0.027	-	-0.33 [CI -0.53 to -0.13]; P<0.001	-	-	-
Shai et al. 2010 [92]	-	-	-	MD vs. LCHO: 0.03 g/L [CI 0.01 to 0.05]; P=0.02 MD vs. LFD: -0.01 g/L [CI -0.03 to 0.01]; P=0.260	-	-
Sola et al. 2011 [98]	-	-	-	MD (EVOO) vs. CG: -0.03 g/L [CI -0.06 to -0.002]; P=0.039 MD (nuts) vs. CG: -0.02 g/L [CI -0.05 to 0.01]; P=0.208	-	-
Thomazella et al. 2011 [101]	-	-	-	0.10 g/L[CI 0.06 to 0.14]; P<0.001	-	-
Troseid et al. 2009 [105]	-0.4 vs. -0.2 mmol/L; P<0.001	-	-	-	-	-
Vincent-Baudry et al. 2005 [107]	-	-	-	-0.01 g/L [CI -0.02 to 0.00]; P=0.106	-	-
Wade et al. 2019 [109]	-	-	-0.05 [CI -0.16 to 0.06]; P=0.380	-	-	-

ALT, alanine aminotransferase; ApoB, apolipoprotein B; CG, control group; CI, confidence intervals; EVOO, extra virgin olive oil; GGT, gamma glutamyl transferase; HbA1c, glycosylated heamoglobin; HDL, high density lipoprotein; HOMA-IR, homeostatic model assessment of insulin resistance; LCHO, low carbohydrate diet; LFD, low-fat diet; MD, Mediterranean diet; LDL, low density lipoprotein; TC, total cholesterol; TG, triglycerides.
Triglyceride concentrations were transformed from mmol/L to mg/dL by multiplying with 88.57; Total, HDL- and LDL- cholesterol concentrations were transformed from mmol/L to mg/dL by multiplying with 38.67; Insulin concentrations were transformed from pmol/L to μU/mL by multiplying with 0.144; Glucose concentrations were transformed from mmol/L to mg/dL by multiplying with 18 (https://www.ncbi.nlm.nih.gov/books/NBK83505/; http://www.endmemo.com/medical/unitconvert/Insulin.php; https://www.diabetes.co.uk/blood-sugar-converter.html).

Summary of findings: The MD, as compared to a control condition, showed a protective effect for glucose in 0/2 papers, for insulin in 0/1 paper, for HOMA-IR index in 0/2 papers, for HbA1c in 0/5 comparisons, for total cholesterol in 1/3 papers, for LDL-cholesterol in 1/2 papers, for HDL-cholesterol in 2/4 papers, for triglycerides in 2/5 papers, for non-HDL-cholesterol in 1/1 paper, for total:HDL-cholesterol ratio in 2/3 papers, for apolipoprotein B in 4/9 comparisons, for alanine aminotransferase in 0/1 paper and for gamma glutamyl transferase in 0/1 paper.

Supplementary Table S9. Summary of the findings on oxidative stress, inflammatory and endothelial function markers (between-group differences) from the papers (and/or outcomes) not included in the pooled analysis

	TAC	CRP	IL-6	Adiponectin	TNF-a	IMT
Esposito et al. 2003 [50]	-	-0.8 mg/L [CI -2.0 to -0.04]; P=0.008	-1.1 pg/mL [CI -1.7 to -0.6]; P=0.009	-	-	-
Esposito et al. 2004 [49]	-	-1 mg/L [CI -1.7 to -0.3]; P=0.010	-0.6 pg/mL [CI -1.1 to -0.1]; P=0.040	-	-	-
Hjerkinn et al. 2006 [60]	-	-	-	-	-	-0.03 mm [CI -0.05 to -0.005]; P=0.017
Maiorino et al. 2016 [67]	-	-0.8mg/L [CI -1.3 to -0.3]; P=0.010	-	1.9 µg/mL [CI 0.8 to 3.0]; P=0.001	-	-
Mayr et al. 2019 [68]	-	-	-	0.0019 µg/mL [CI 0.0014 to 0.0024]; P<0.001	-	-
Ortner Hadžiabdić et al. 2016 [75]	0.15 mmol Trolox [CI 0.11 to 0.19]; P<0.001	-	-	-	-	-
Richard et al. 2013 [86]	-	-26.1%, P=0.019	-4.8%, P=0.318	-	-4.1%, P=0.290	-
Sala-Vila et al. 2014 [90]	-	-	-	-	-	MD (EVOO) vs. CG: -0.03 mm [CI -0.07 to 0.01]; P=0.199<br>MD (nuts) vs. CG: -0.02 mm [CI -0.06 to 0.02]; P=0.396
Shai et al. 2010 [92]	-	-	-	-	-	MD vs. LCHO: 0.03 mm [CI 0.01 to 0.06]; P=0.011<br>MD vs. LFD: 0.02 mm [CI 0.003 to 0.04]; P=0.026
Sofi et al. 2018 [97]	0.23 µmol/mL [CI 0.05 to 0.41]; P=0.014	-	-0.16 pg/mL [CI -0.19 to -0.12]; P<0.001	-	-	-
Storniolo et al. 2017 [100]	MD (EVOO) vs. CG: 0.30 mM Trolox [CI 0.25 to 0.36]; P<0.001<br>MD (nuts) vs. CG: 0.06 mM Trolox [CI 0.02 to 0.10]; P=0.010	-	-	-	-	-
Troseid et al. 2009 [105]	-	-0.34 vs. -0.32 mg/L; P=0.523	-0.2 vs. -0.21 pg/mL; P=0.871	-0.72 vs. -0.11µg/mL; P=0.722	-0.14 vs. -0.1pg/mL; P=0.963	-

CG, contol group; CI, confidence intervals; CRP, C-reactive protein; EVOO, extra virgin olive oil; IL-6, interleukin 6; IMT, intima-media thickness; LCHO, low carbohydrate diet; LFD, low-fat diet; MD, Mediterranean diet; TAC, total antioxidant capacity; TNF-a, tumour necrosis factor a.

Summary of findings: The MD, as compared to a control condition, showed a protective effect for total antioxidant capacity in 4/4 comparisons, for C-reactive protein in 4/5 papers, for interleukin-6 in 3/5 papers, for adiponectin in 2/3 papers, for tumour necrosis factor-a in 0/2 papers and for intima-media thickness in 1/5 comparisons.

Supplementary Figures S1–S28. Forest plots of controlled trials evaluating the effect of the Mediterranean diet on anthropometric, blood pressure, biochemical, insulin resistance, oxidative stress, inflammatory and endothelial function markers related to the metabolic syndrome

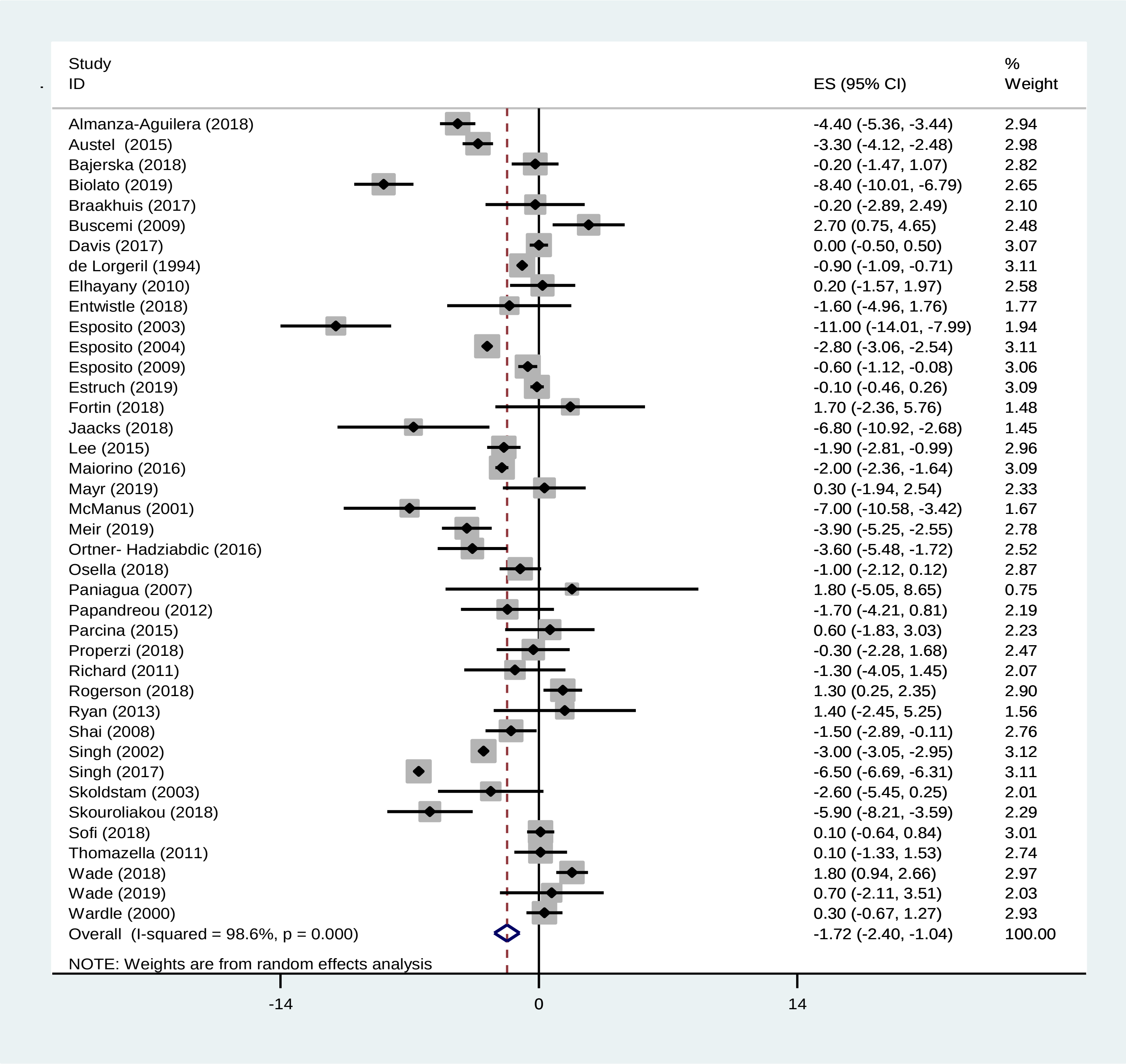

Mean differences were pooled using random effects meta-analysis. Squares indicate effect size for each study (mean difference between intervention and control group), with extended lines representing 95% confidence intervals (95% CIs). Diamonds indicate the overall weighted mean effect size. I^2 indicates between-study heterogeneity.
Detailed study characteristics can be found in Table 1 (main manuscript).

***Figure S1.* Forest plot for overall body weight estimate**

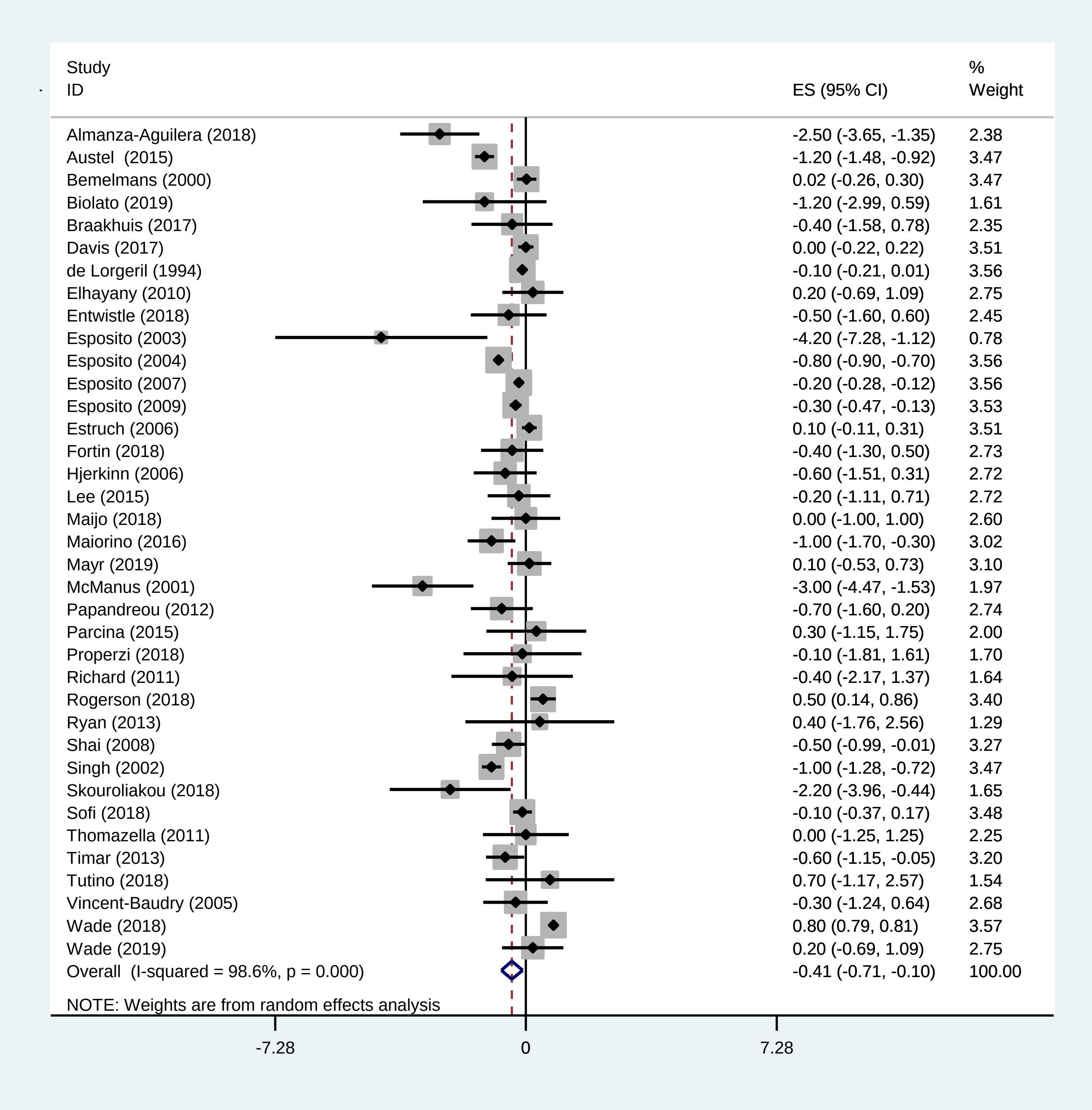

Mean differences were pooled using random effects meta-analysis. Squares indicate effect size for each study (mean difference between intervention and control group), with extended lines representing 95% confidence intervals (95% CIs). Diamonds indicate the overall weighted mean effect size. I^2 indicates between-study heterogeneity.
Detailed study characteristics can be found in Table 1 (main manuscript).

***Figure S2.* Forest plot for overall body mass index estimate**

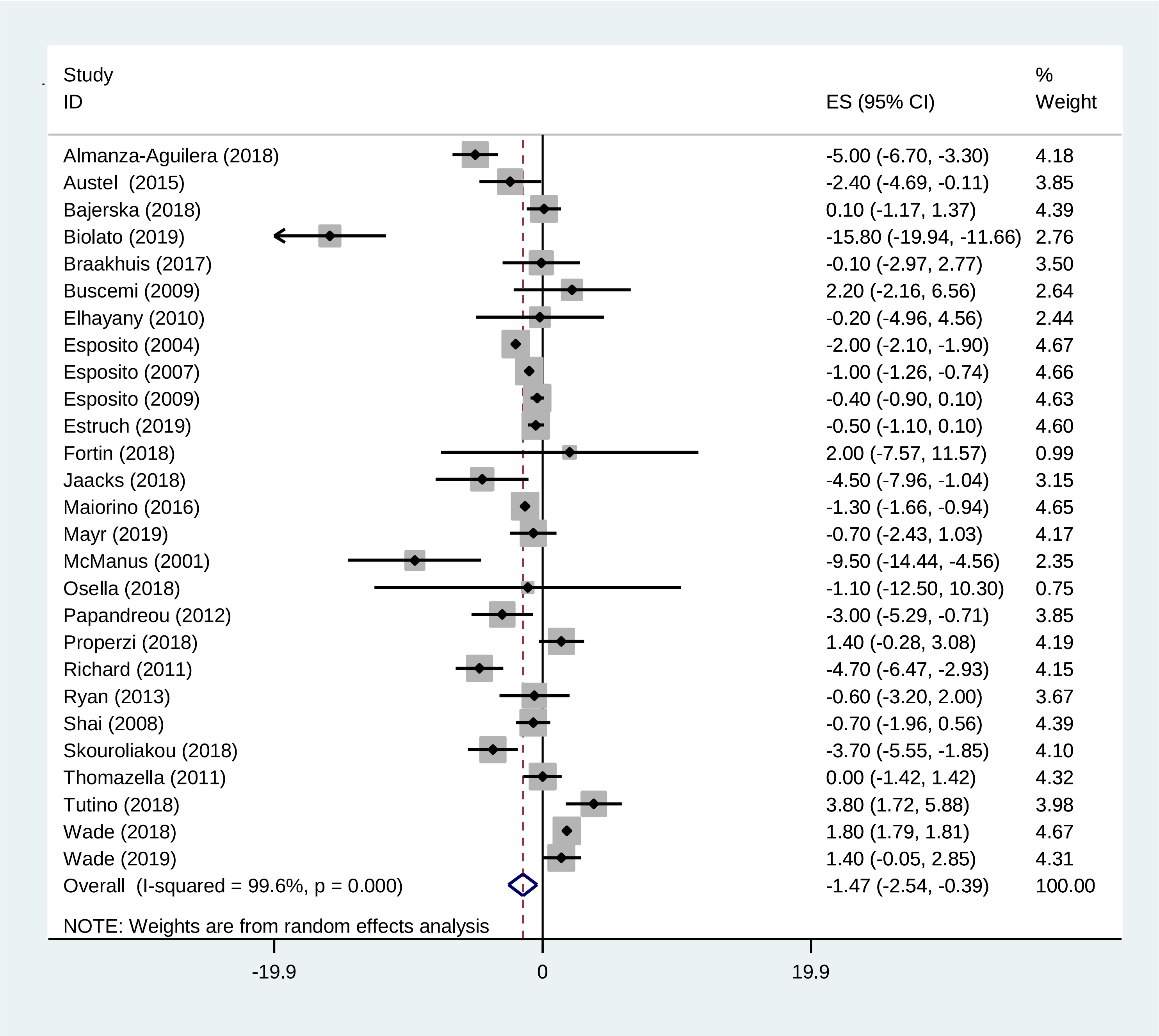

Mean differences were pooled using random effects meta-analysis. Squares indicate effect size for each study (mean difference between intervention and control group), with extended lines representing 95% confidence intervals (95% CIs). Diamonds indicate the overall weighted mean effect size. I^2 indicates between-study heterogeneity.
Detailed study characteristics can be found in Table 1 (main manuscript).

Figure S3: **Forest plot for overall waist circumference**

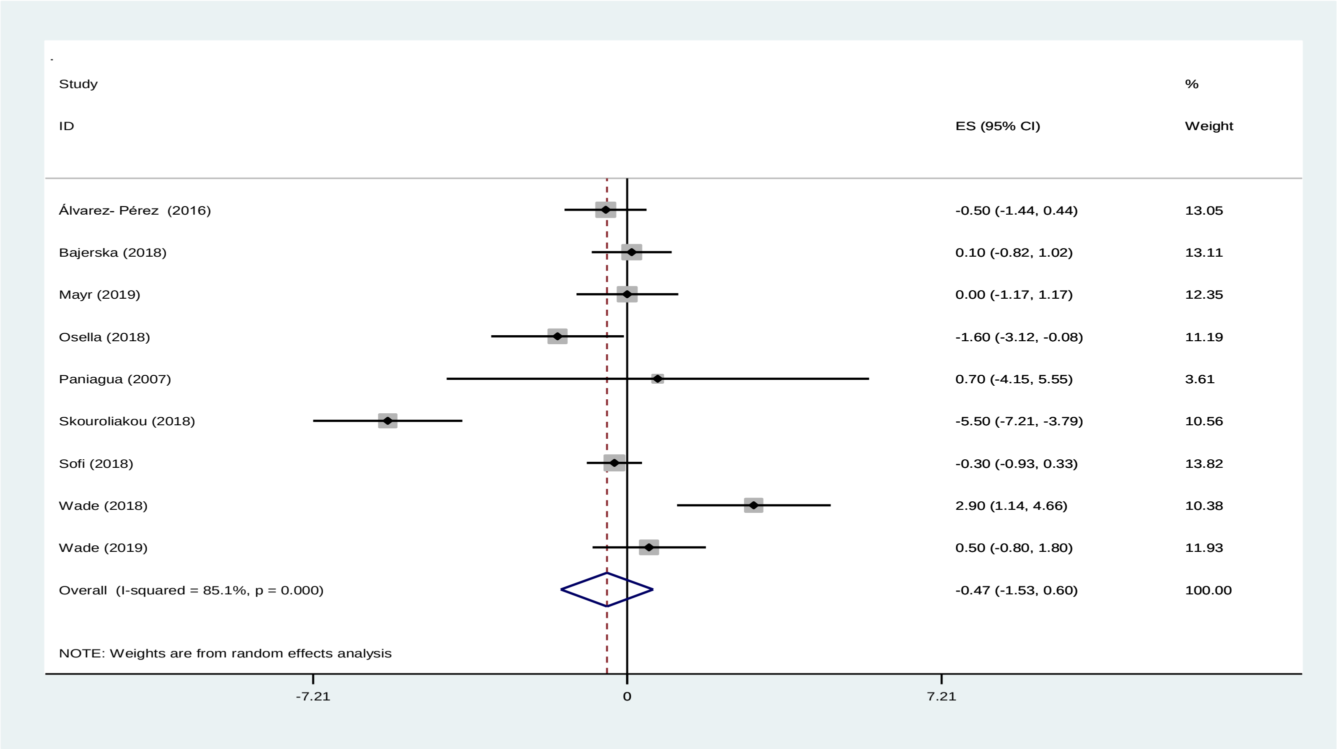

Mean differences were pooled using random effects meta-analysis. Squares indicate effect size for each study (mean difference between intervention and control group), with extended lines representing 95% confidence intervals (95% CIs). Diamonds indicate the overall weighted mean effect size. I^2 indicates between-study heterogeneity.
Detailed study characteristics can be found in Table 1 (main manuscript).

Figure S4: **Forest plot for overall total fat mass (kg)**

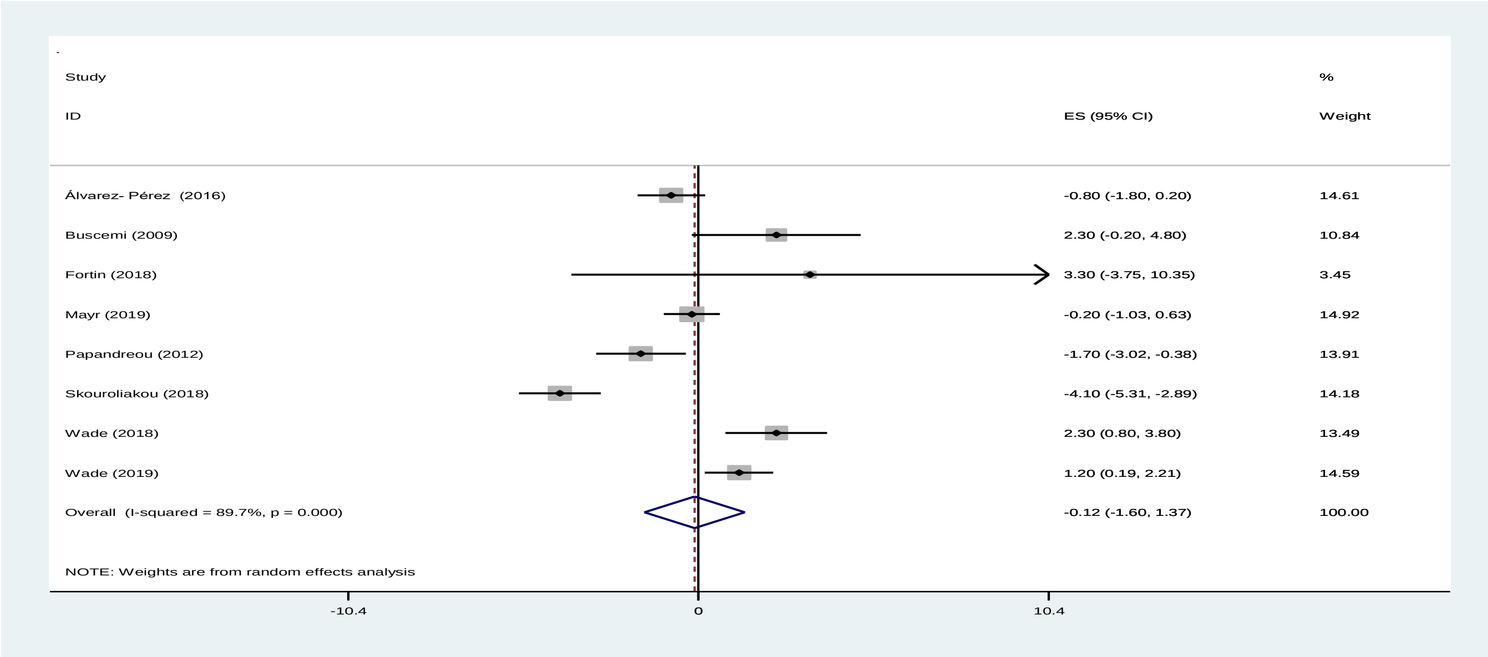

Mean differences were pooled using random effects meta-analysis. Squares indicate effect size for each study (mean difference between intervention and control group), with extended lines representing 95% confidence intervals (95% CIs). Diamonds indicate the overall weighted mean effect size. I^2 indicates between-study heterogeneity.
Detailed study characteristics can be found in Table 1 (main manuscript).

Figure S5: **Forest plot for overall total body fat (%)**

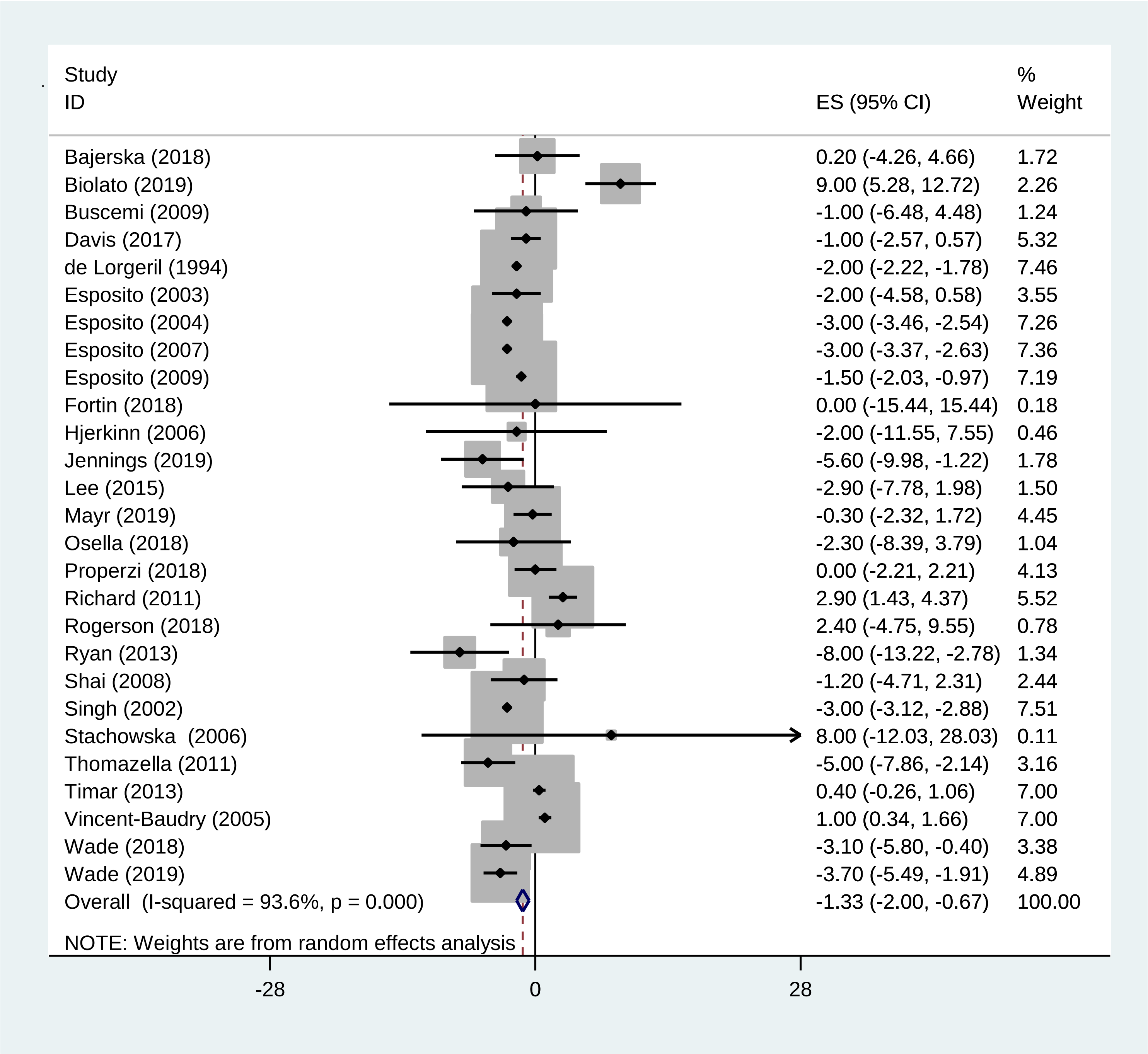

Mean differences were pooled using random effects meta-analysis. Squares indicate effect size for each study (mean difference between intervention and control group), with extended lines representing 95% confidence intervals (95% CIs). Diamonds indicate the overall weighted mean effect size. I^2 indicates between-study heterogeneity.
Detailed study characteristics can be found in Table 1 (main manuscript).

Figure S6: **Forest plot for overall systolic blood pressure**

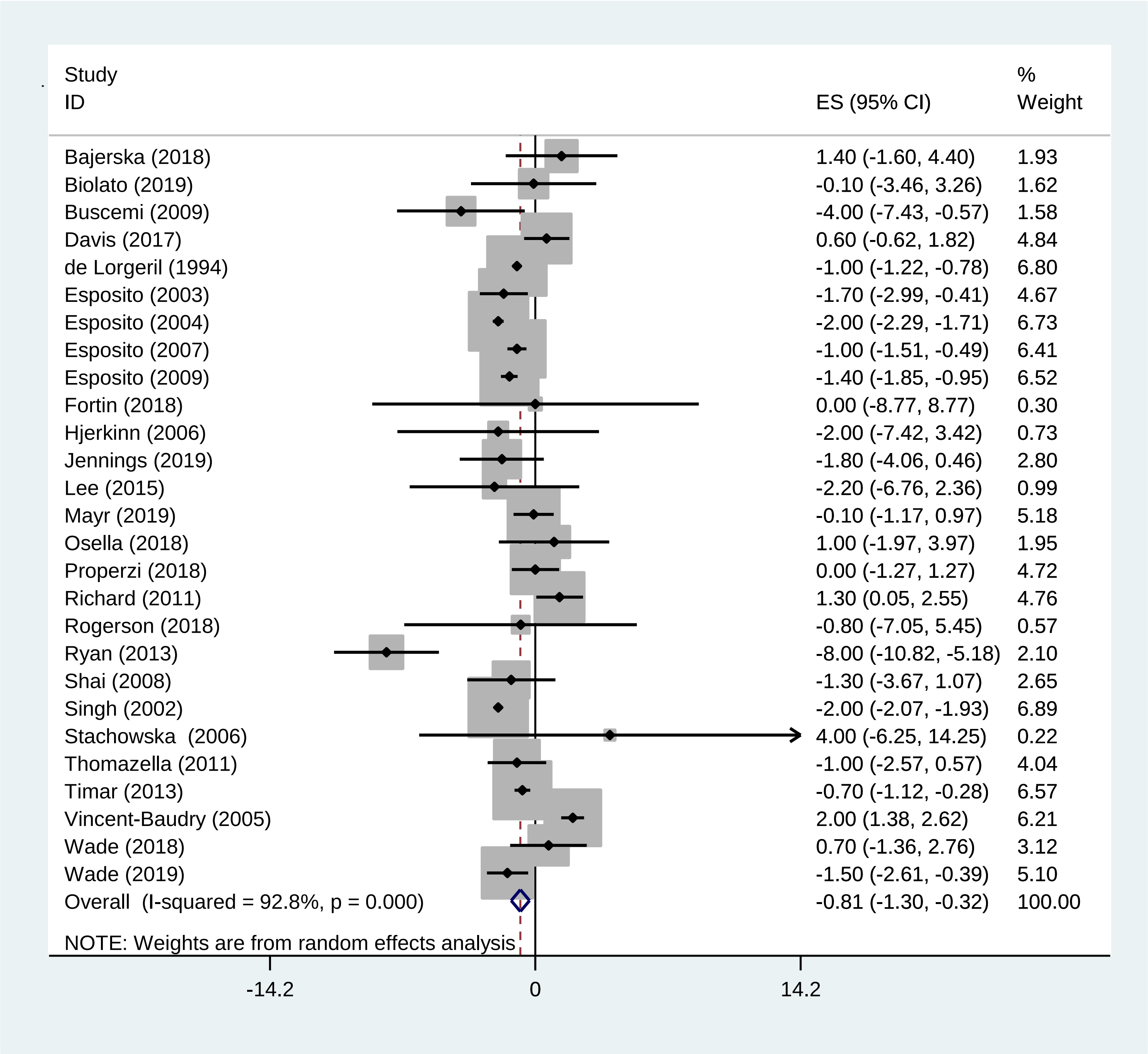

Mean differences were pooled using random effects meta-analysis. Squares indicate effect size for each study (mean difference between intervention and control group), with extended lines representing 95% confidence intervals (95% CIs). Diamonds indicate the overall weighted mean effect size. I^2 indicates between-study heterogeneity.

Detailed study characteristics can be found in Table 1 (main manuscript).

Figure S7: **Forest plot for overall diastolic blood pressure**

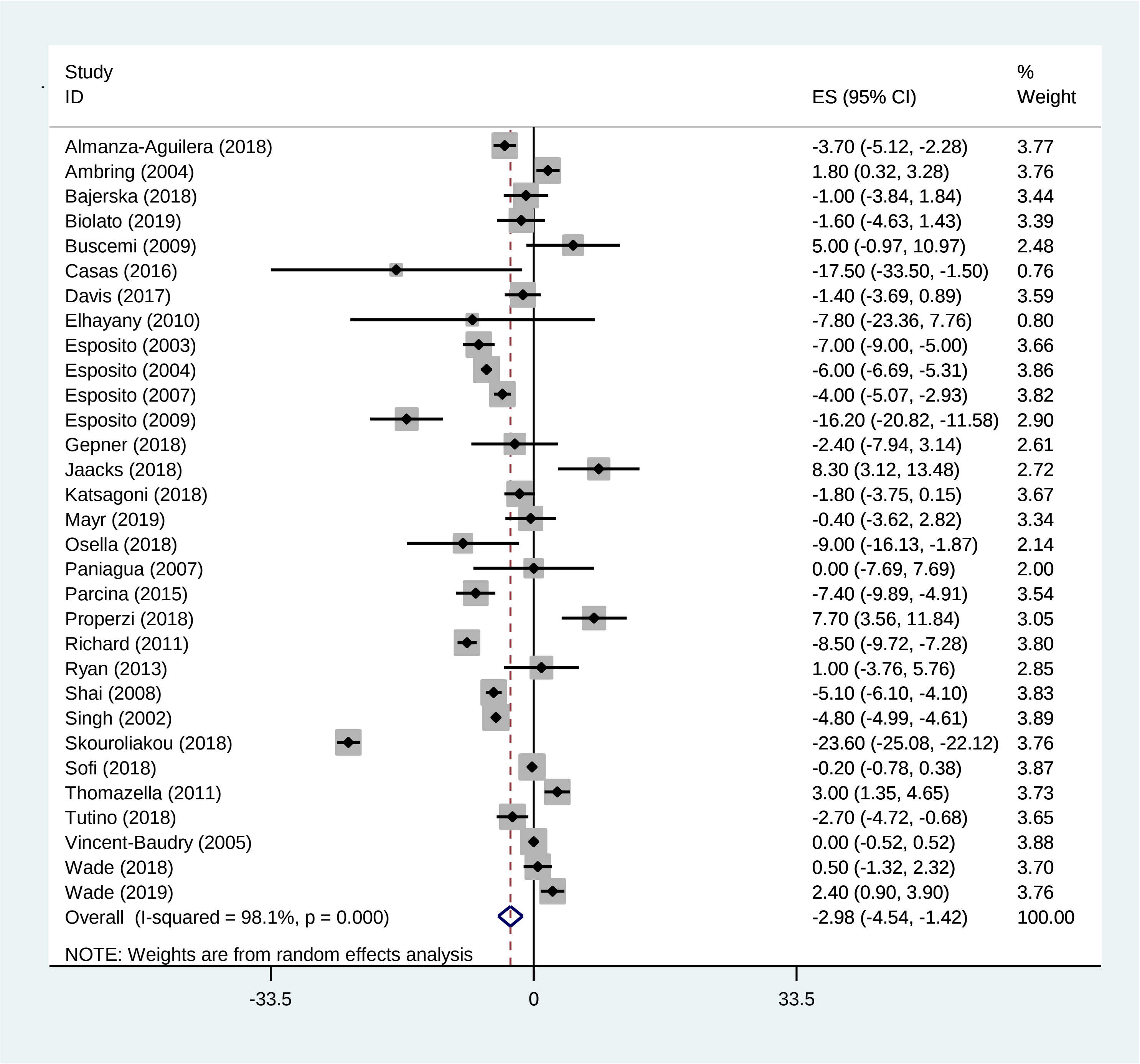

Mean differences were pooled using random effects meta-analysis. Squares indicate effect size for each study (mean difference between intervention and control group), with extended lines representing 95% confidence intervals (95% CIs). Diamonds indicate the overall weighted mean effect size. I^2 indicates between-study heterogeneity.
Detailed study characteristics can be found in Table 1 (main manuscript).

Figure S8: **Forest plot for overall blood glucose**

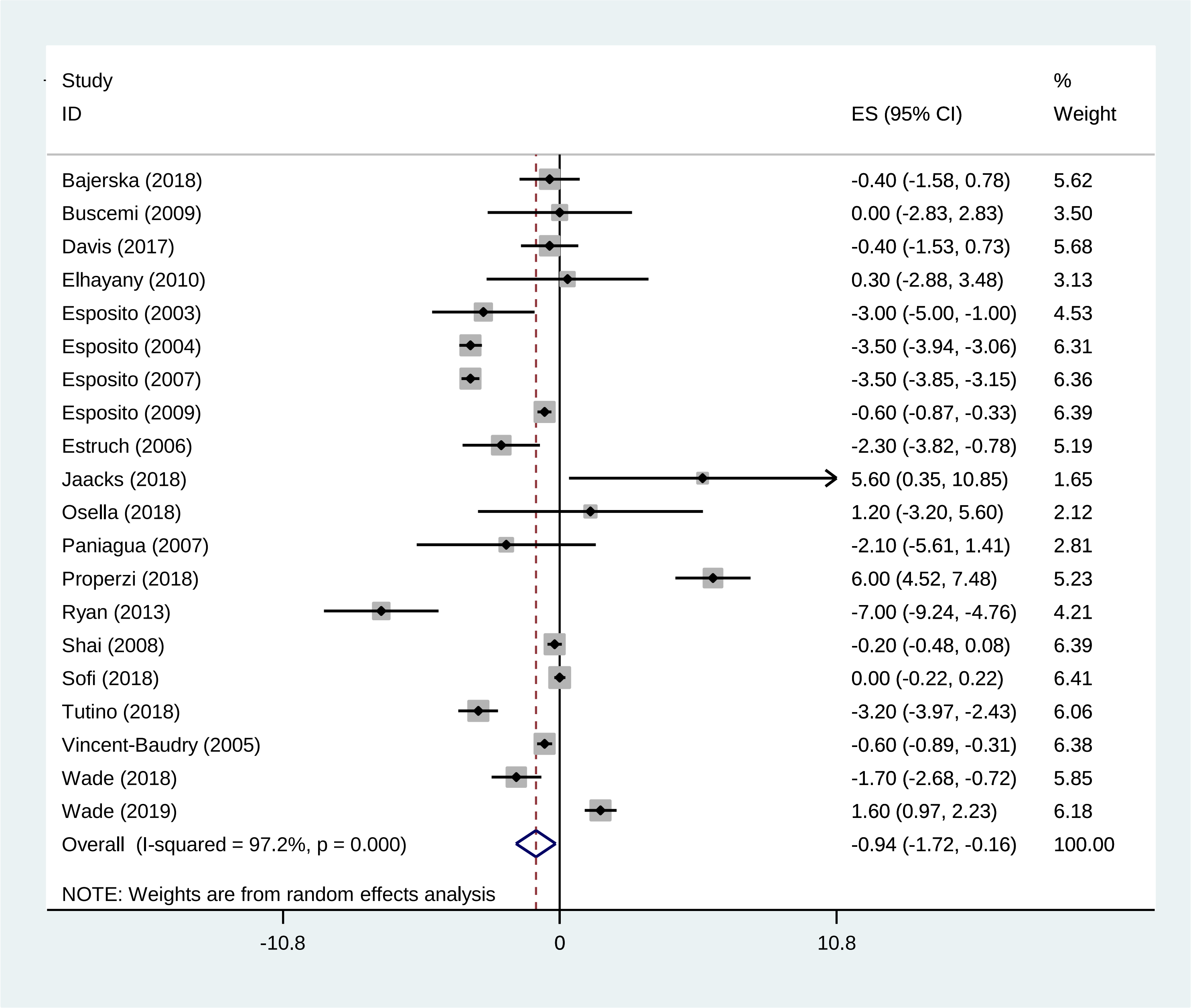

Mean differences were pooled using random effects meta-analysis. Squares indicate effect size for each study (mean difference between intervention and control group), with extended lines representing 95% confidence intervals (95% CIs). Diamonds indicate the overall weighted mean effect size. I^2 indicates between-study heterogeneity.
Detailed study characteristics can be found in Table 1 (main manuscript).

Figure S9: **Forest plot for overall blood insulin**

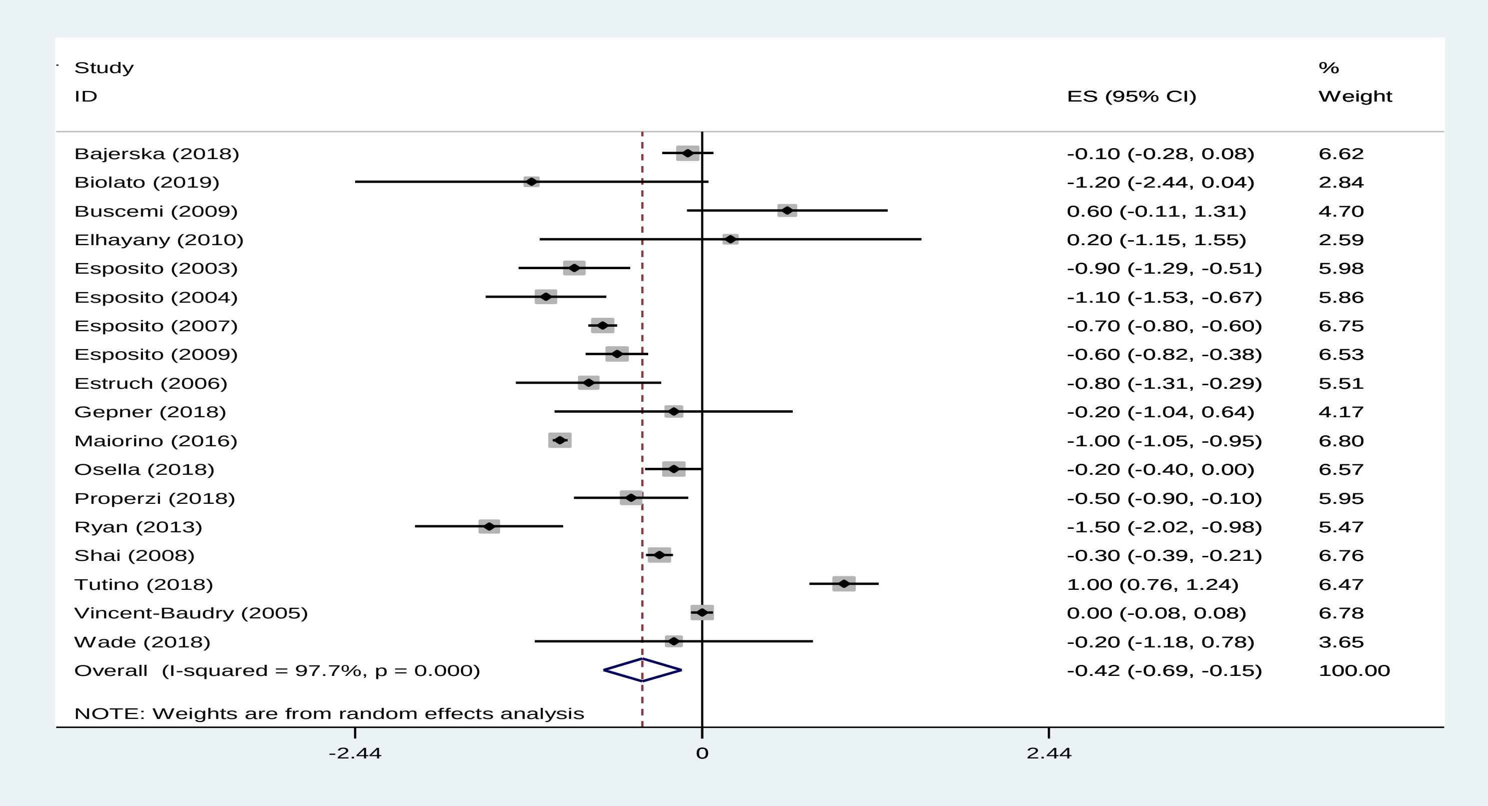

Mean differences were pooled using random effects meta-analysis. Squares indicate effect size for each study (mean difference between intervention and control group), with extended lines representing 95% confidence intervals (95% CIs). Diamonds indicate the overall weighted mean effect size. I^2 indicates between-study heterogeneity.
Detailed study characteristics can be found in Table 1 (main manuscript).

Figure S10: **Forest plot for overall HOMA-IR index**

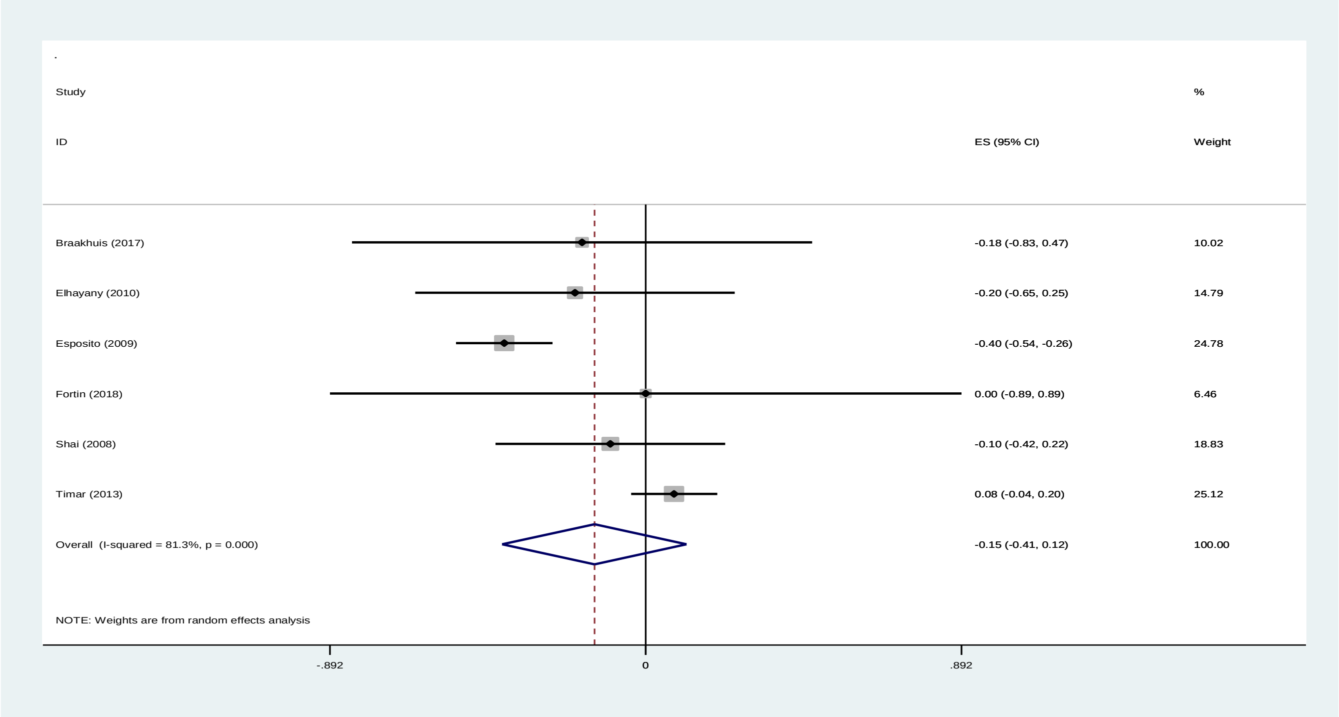

Mean differences were pooled using random effects meta-analysis. Squares indicate effect size for each study (mean difference between intervention and control group), with extended lines representing 95% confidence intervals (95% CIs). Diamonds indicate the overall weighted mean effect size. I^2 indicates between-study heterogeneity.
Detailed study characteristics can be found in Table 1 (main manuscript).

Figure S11: **Forest plot for overall glycosylated hemoglobin (HbA1c)**

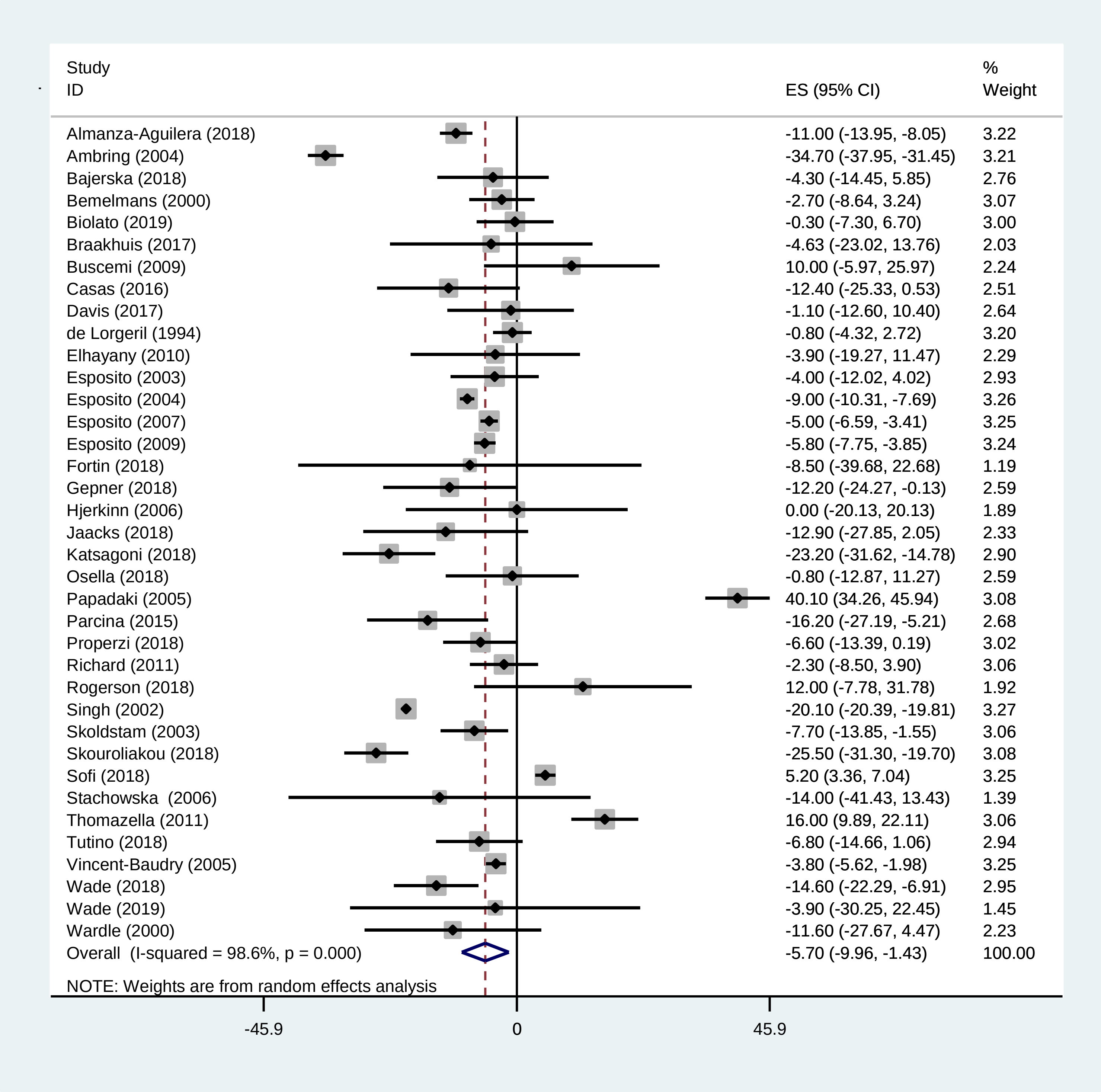

Mean differences were pooled using random effects meta-analysis. Squares indicate effect size for each study (mean difference between intervention and control group), with extended lines representing 95% confidence intervals (95% CIs). Diamonds indicate the overall weighted mean effect size. I^2 indicates between-study heterogeneity.
Detailed study characteristics can be found in Table 1 (main manuscript).

Figure S12: **Forest plot for overall blood total cholesterol**

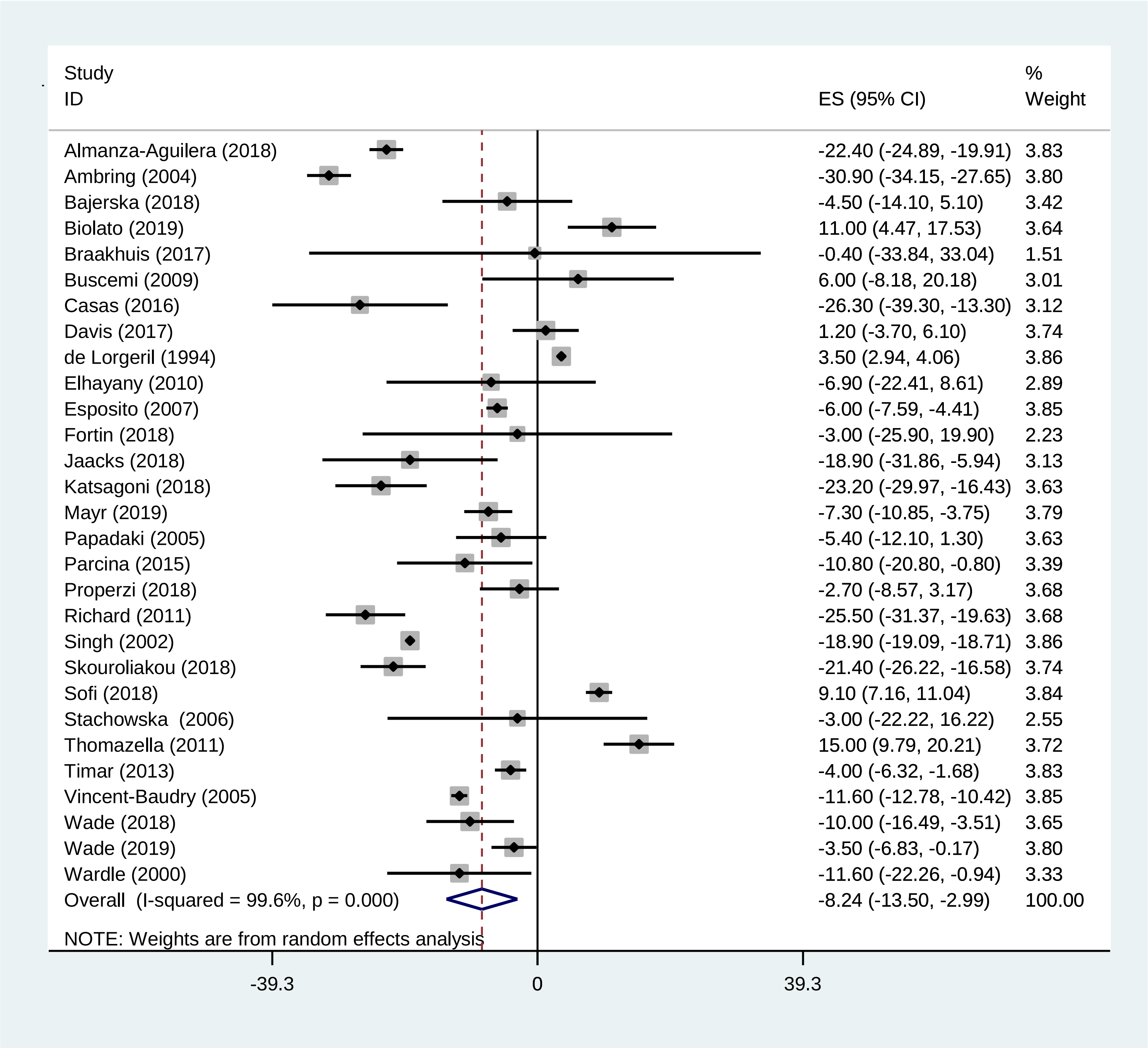

Mean differences were pooled using random effects meta-analysis. Squares indicate effect size for each study (mean difference between intervention and control group), with extended lines representing 95% confidence intervals (95% CIs). Diamonds indicate the overall weighted mean effect size. I^2 indicates between-study heterogeneity.
Detailed study characteristics can be found in Table 1 (main manuscript).

***Figure S13:* Forest plot for overall blood LDL-cholesterol**

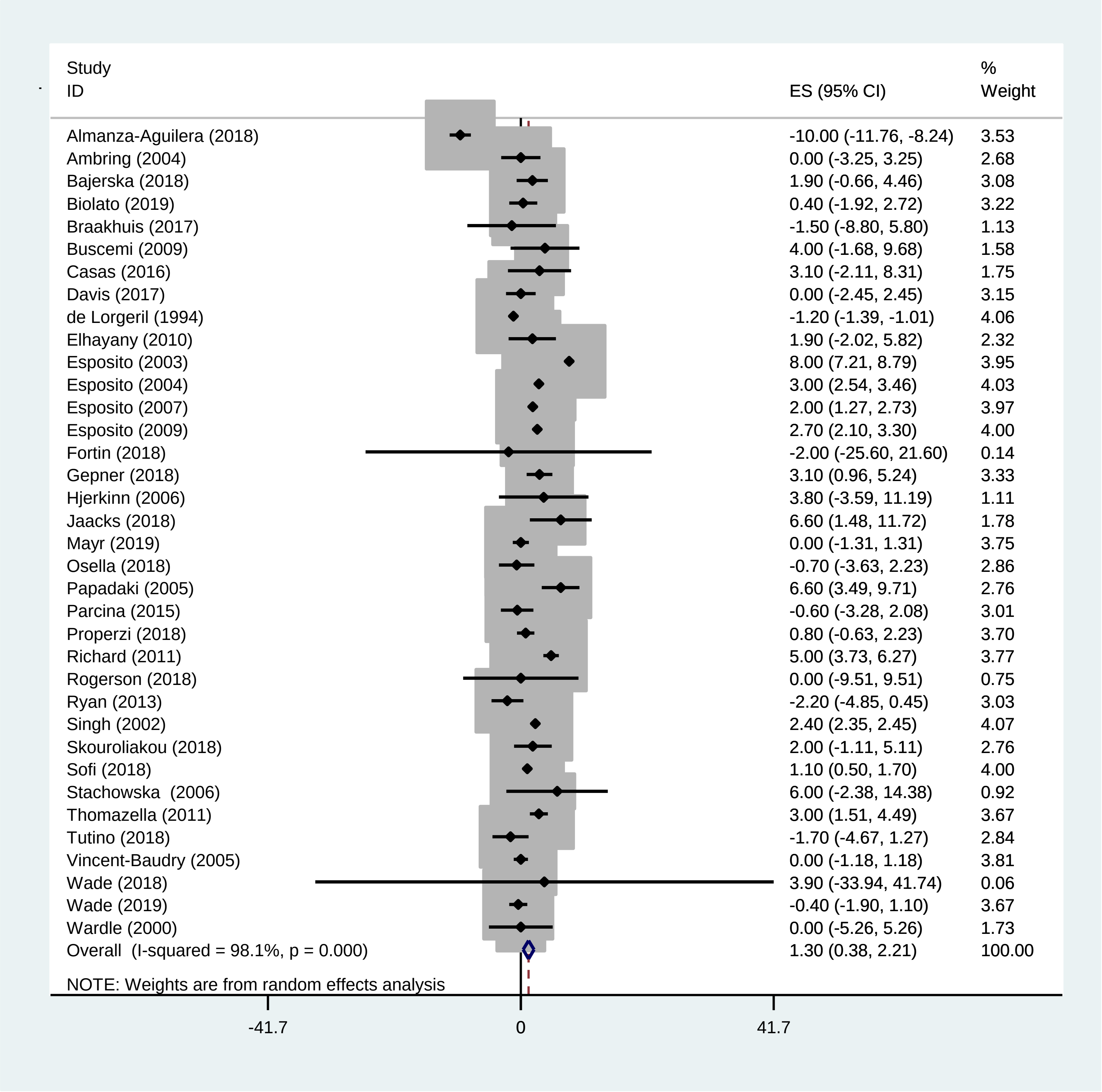

Mean differences were pooled using random effects meta-analysis. Squares indicate effect size for each study (mean difference between intervention and control group), with extended lines representing 95% confidence intervals (95% CIs). Diamonds indicate the overall weighted mean effect size. I^2 indicates between-study heterogeneity.

Detailed study characteristics can be found in Table 1 (main manuscript).

Figure S14: **Forest plot for overall blood HDL-cholesterol**

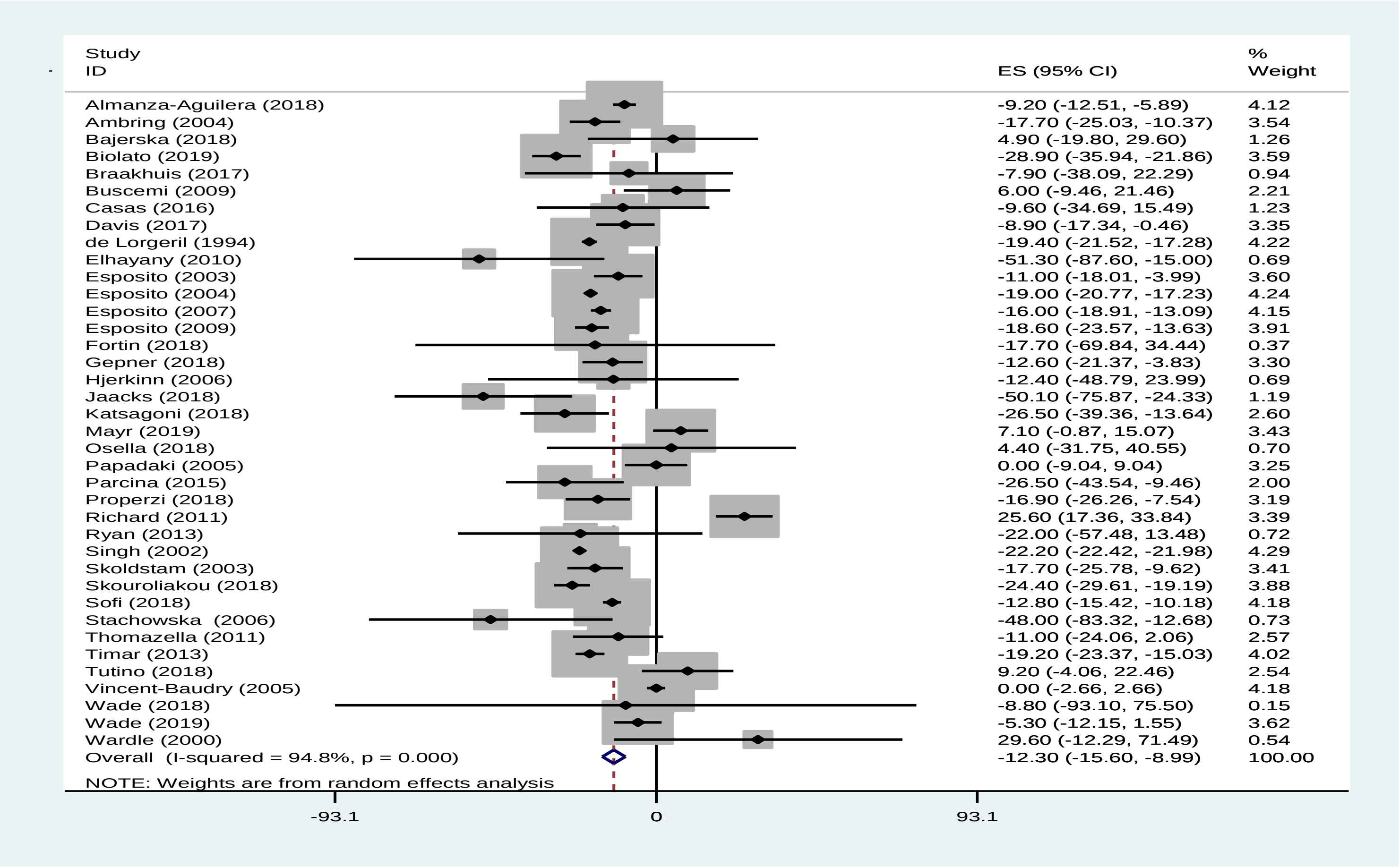

Mean differences were pooled using random effects meta-analysis. Squares indicate effect size for each study (mean difference between intervention and control group), with extended lines representing 95% confidence intervals (95% CIs). Diamonds indicate the overall weighted mean effect size. I^2 indicates between-study heterogeneity.
Detailed study characteristics can be found in Table 1 (main manuscript).

Figure S15: **Forest plot for overall blood triglycerides**

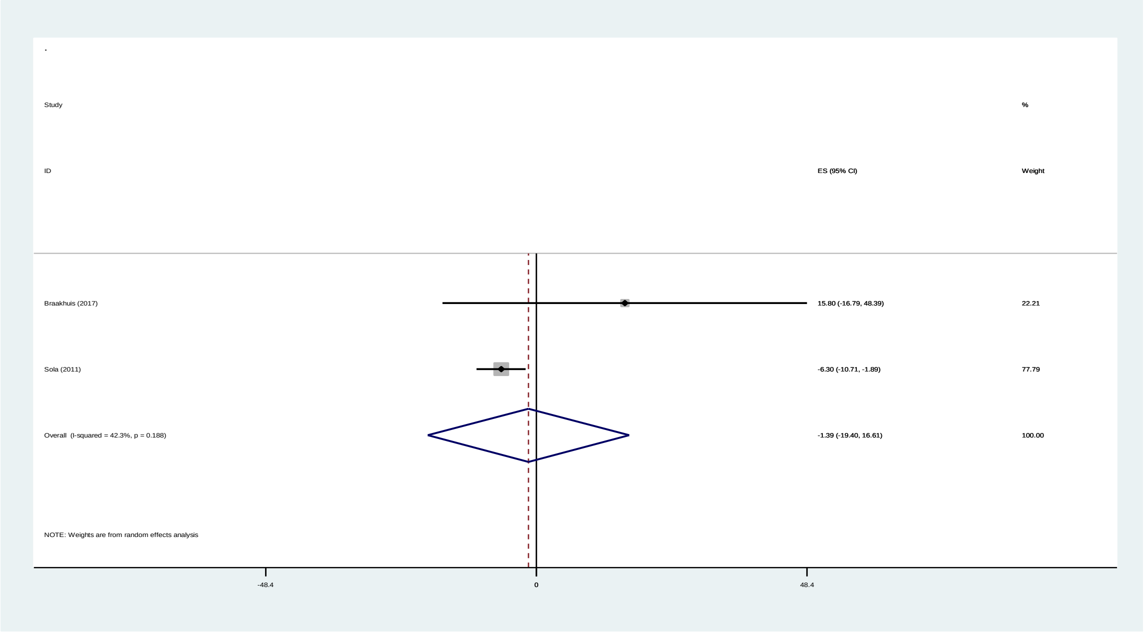

Mean differences were pooled using random effects meta-analysis. Squares indicate effect size for each study (mean difference between intervention and control group), with extended lines representing 95% confidence intervals (95% CIs). Diamonds indicate the overall weighted mean effect size. I^2 indicates between-study heterogeneity.
Detailed study characteristics can be found in Table 1 (main manuscript).

Figure S16: **Forest plot for overall blood non-HDL cholesterol**

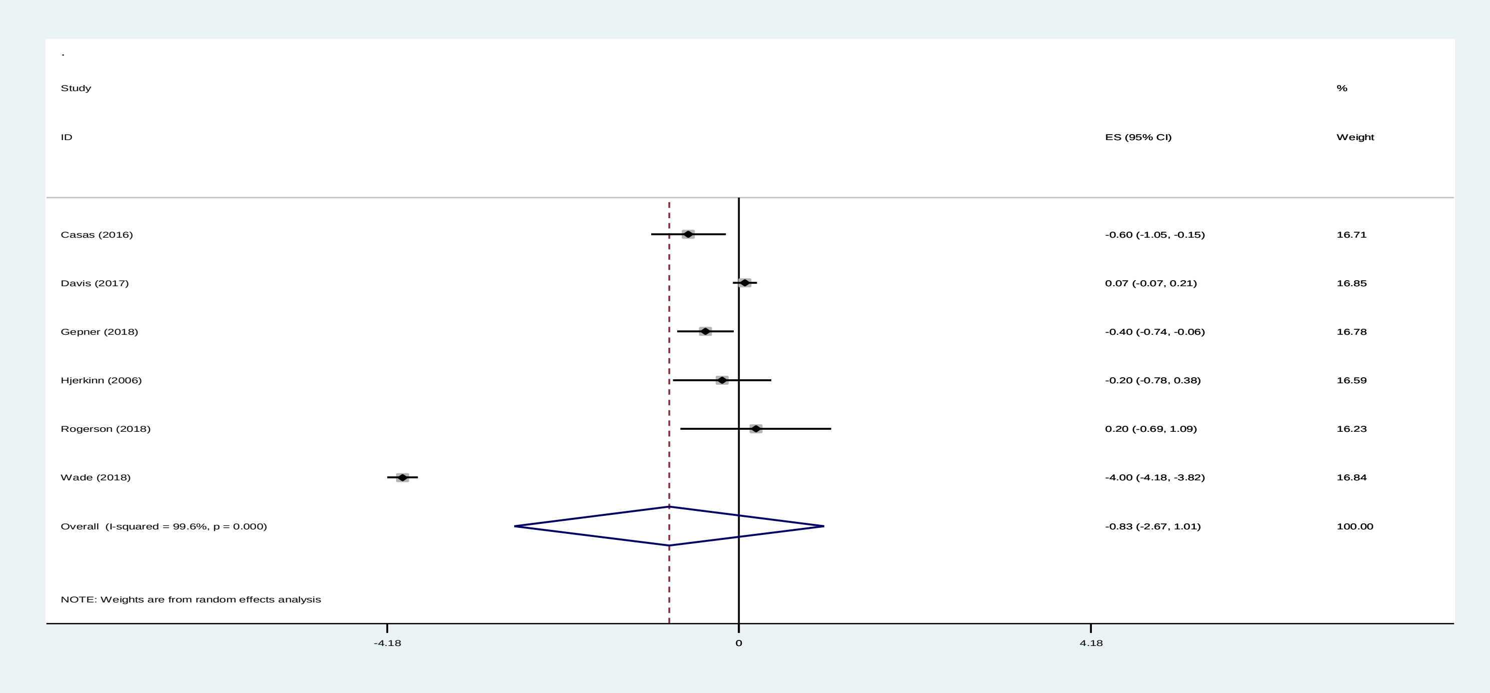

Mean differences were pooled using random effects meta-analysis. Squares indicate effect size for each study (mean difference between intervention and control group), with extended lines representing 95% confidence intervals (95% CIs). Diamonds indicate the overall weighted mean effect size. I^2 indicates between-study heterogeneity.
Detailed study characteristics can be found in Table 1 (main manuscript).

Figure S17: **Forest plot for overall blood total:HDL cholesterol ratio**

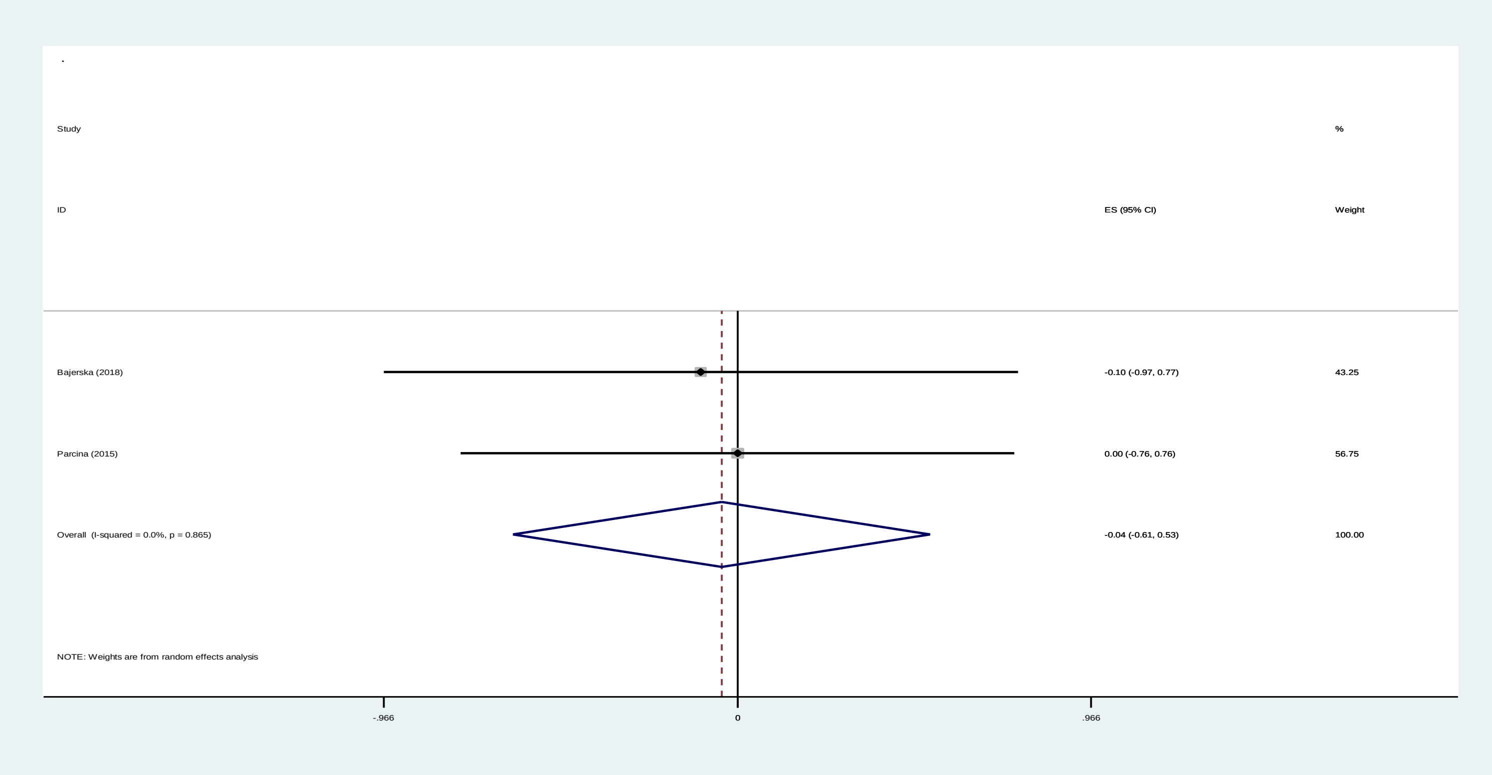

Mean differences were pooled using random effects meta-analysis. Squares indicate effect size for each study (mean difference between intervention and control group), with extended lines representing 95% confidence intervals (95% CIs). Diamonds indicate the overall weighted mean effect size. I^2 indicates between-study heterogeneity.
Detailed study characteristics can be found in Table 1 (main manuscript).

Figure S18: **Forest plot for overall blood homocysteine**

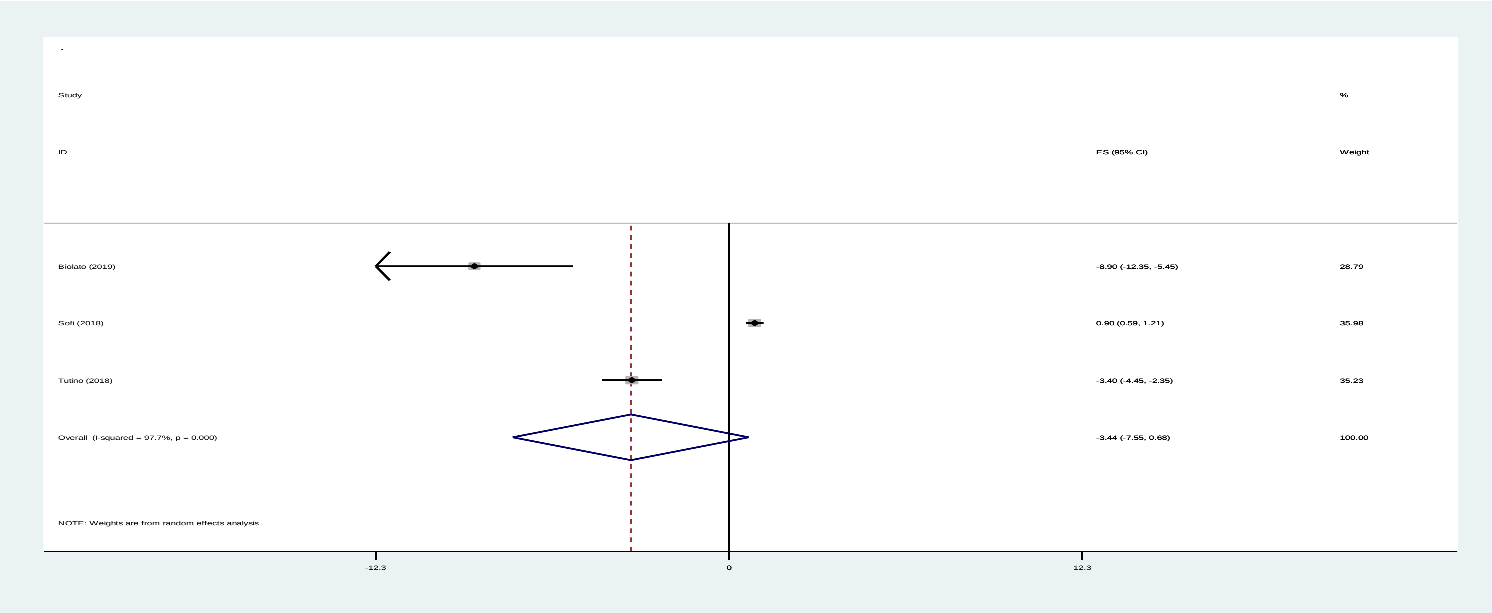

Mean differences were pooled using random effects meta-analysis. Squares indicate effect size for each study (mean difference between intervention and control group), with extended lines representing 95% confidence intervals (95% CIs). Diamonds indicate the overall weighted mean effect size. I^2 indicates between-study heterogeneity.
Detailed study characteristics can be found in Table 1 (main manuscript).

Figure S19: **Forest plot for overall urine aspartame aminotransferase (AST)**

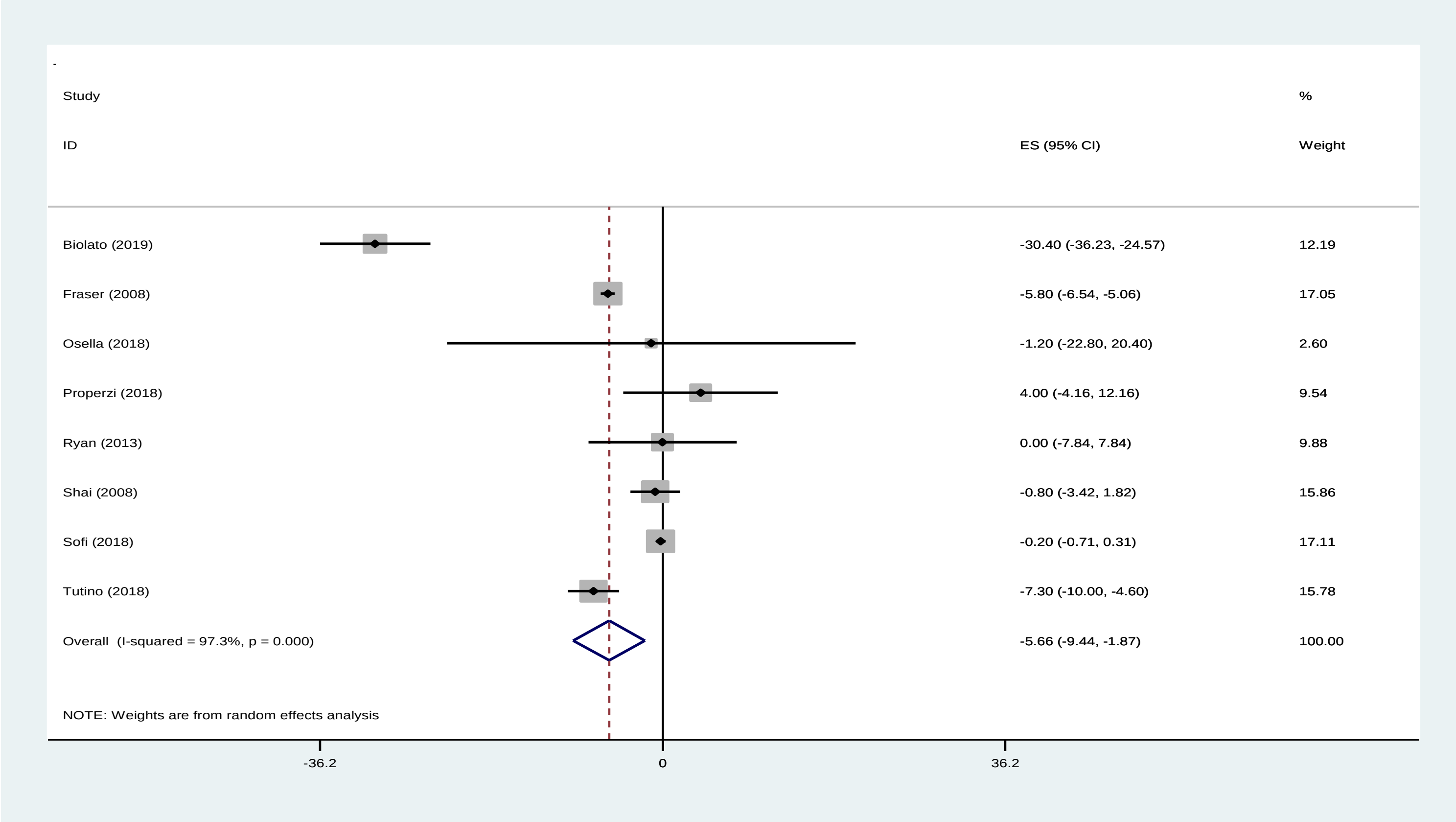

Mean differences were pooled using random effects meta-analysis. Squares indicate effect size for each study (mean difference between intervention and control group), with extended lines representing 95% confidence intervals (95% CIs). Diamonds indicate the overall weighted mean effect size. I^2 indicates between-study heterogeneity.
Detailed study characteristics can be found in Table 1 (main manuscript).

Figure S20: **Forest plot for overall urine alanine aminotransferase (ALT)**

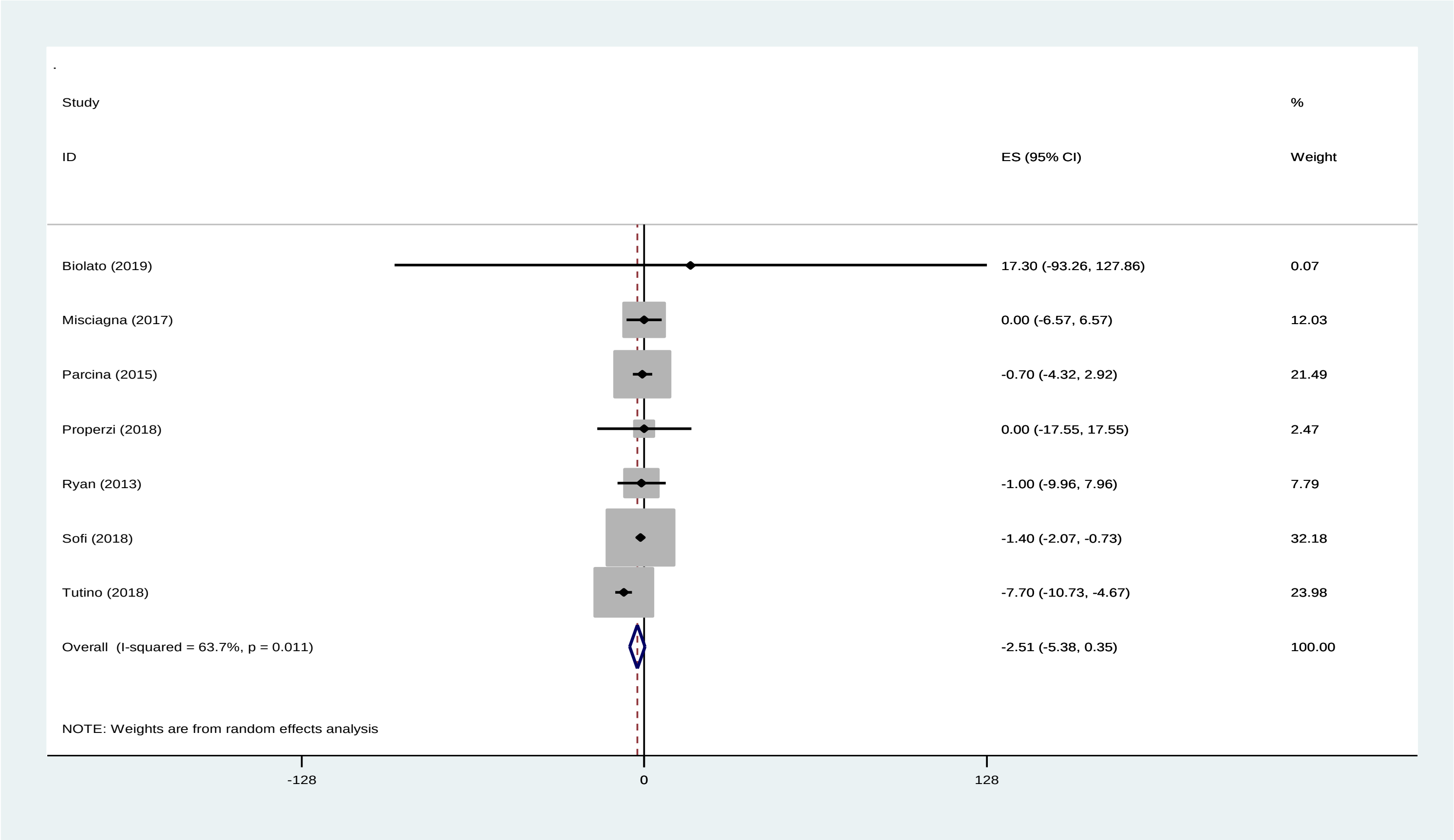

Mean differences were pooled using random effects meta-analysis. Squares indicate effect size for each study (mean difference between intervention and control group), with extended lines representing 95% confidence intervals (95% CIs). Diamonds indicate the overall weighted mean effect size. I^2 indicates between-study heterogeneity.
Detailed study characteristics can be found in Table 1 (main manuscript).

Figure S21: **Forest plot for overall urine gamma glutamyl transferase (GGT)**

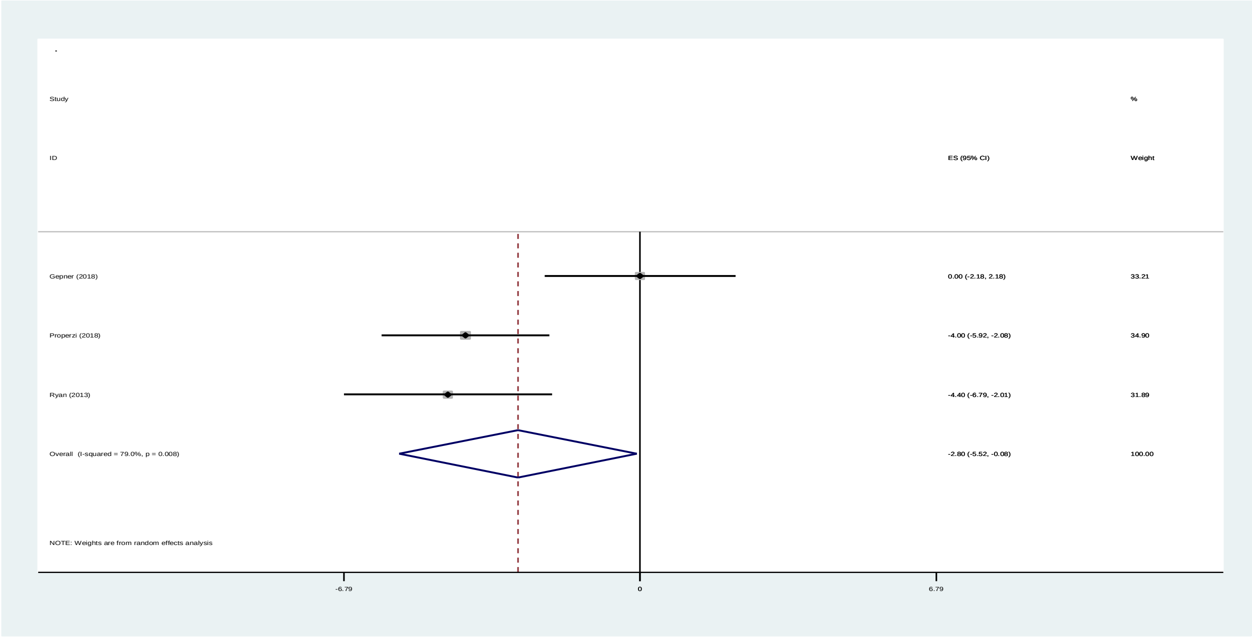

Mean differences were pooled using random effects meta-analysis. Squares indicate effect size for each study (mean difference between intervention and control group), with extended lines representing 95% confidence intervals (95% CIs). Diamonds indicate the overall weighted mean effect size. I^2 indicates between-study heterogeneity.
Detailed study characteristics can be found in Table 1 (main manuscript).

Figure S22: **Forest plot for overall hepatic fat mass**

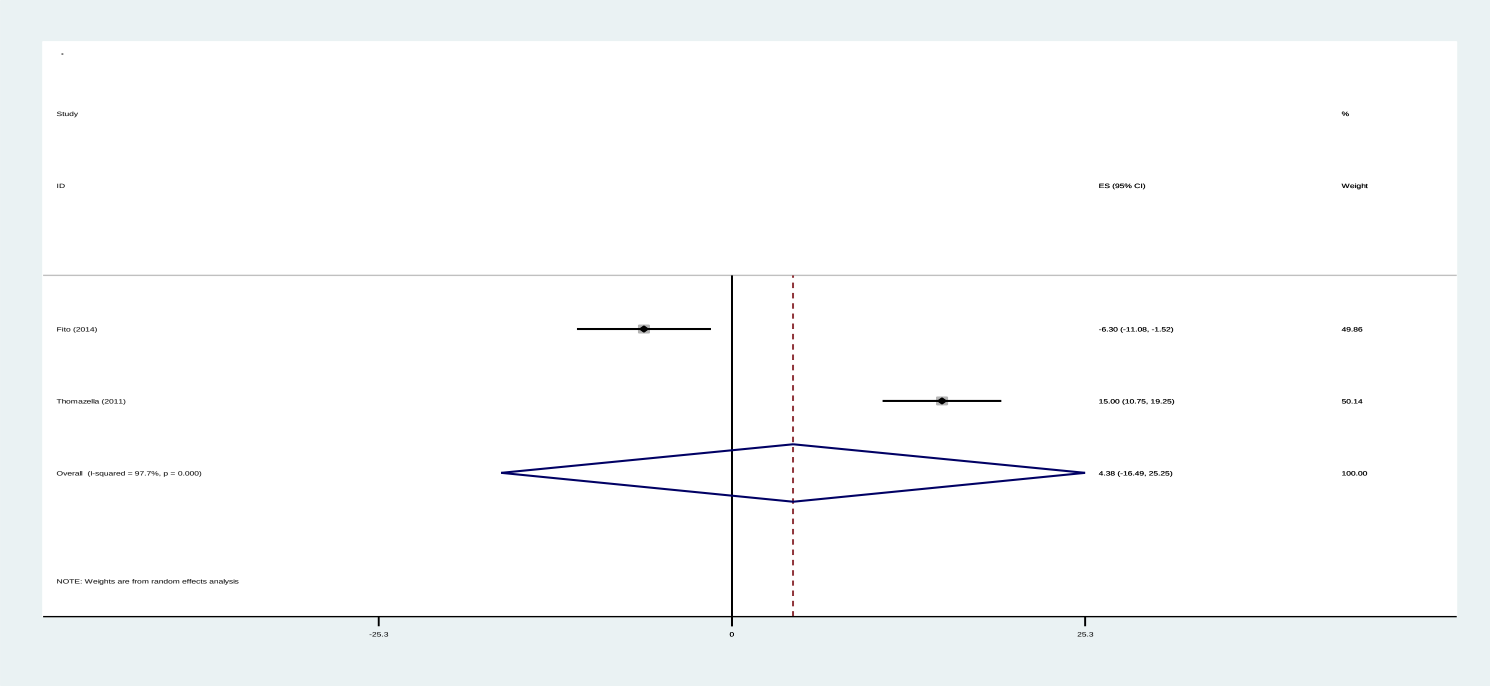

Mean differences were pooled using random effects meta-analysis. Squares indicate effect size for each study (mean difference between intervention and control group), with extended lines representing 95% confidence intervals (95% CIs). Diamonds indicate the overall weighted mean effect size. I^2 indicates between-study heterogeneity.
Detailed study characteristics can be found in Table 1 (main manuscript).

Figure S23: **Forest plot for overall blood oxidised LDL-cholesterol**

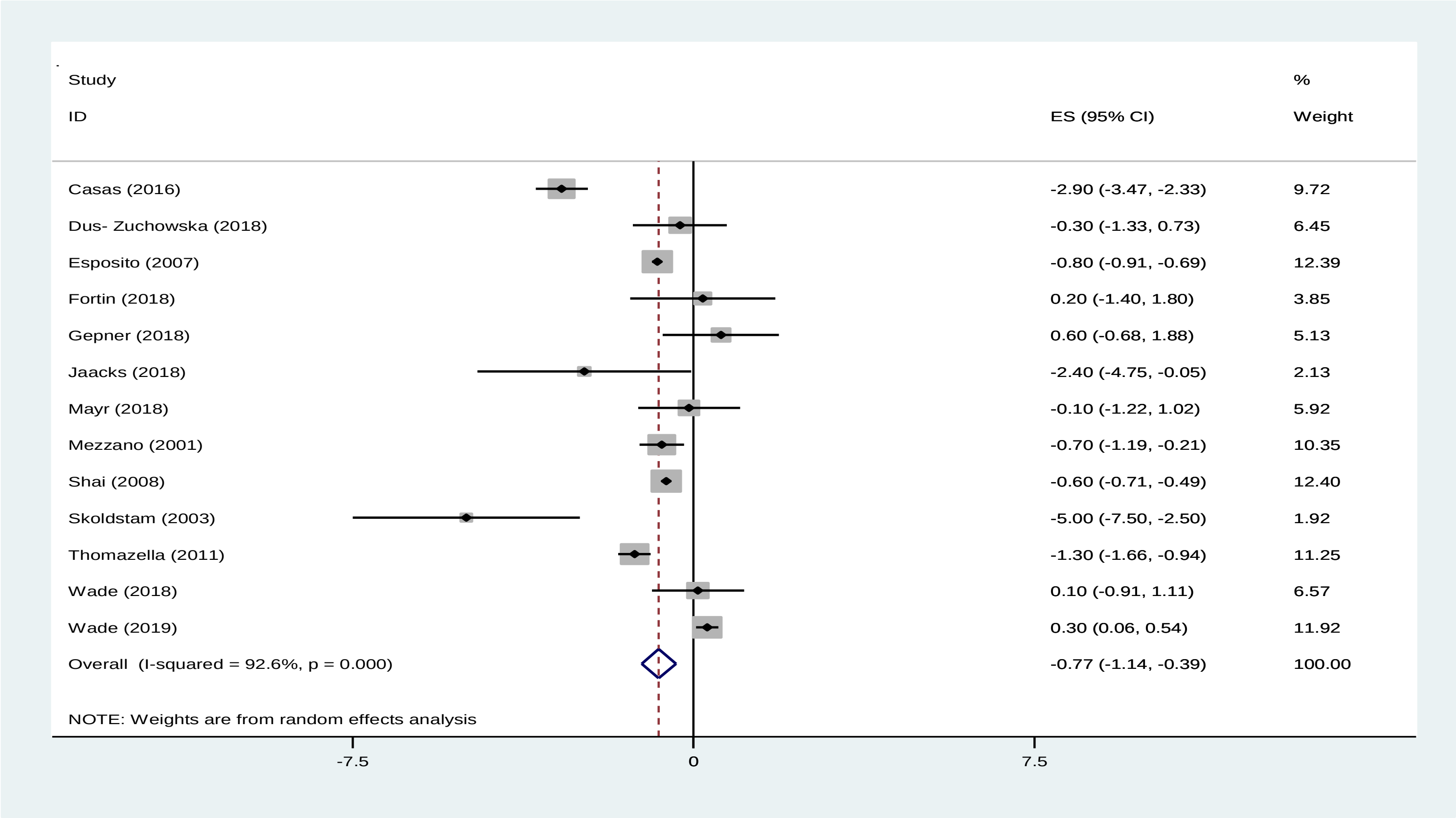

Mean differences were pooled using random effects meta-analysis. Squares indicate effect size for each study (mean difference between intervention and control group), with extended lines representing 95% confidence intervals (95% CIs). Diamonds indicate the overall weighted mean effect size. I^2 indicates between-study heterogeneity.
Detailed study characteristics can be found in Table 1 (main manuscript).

Figure S24: **Forest plot for overall blood C-reactive protein**

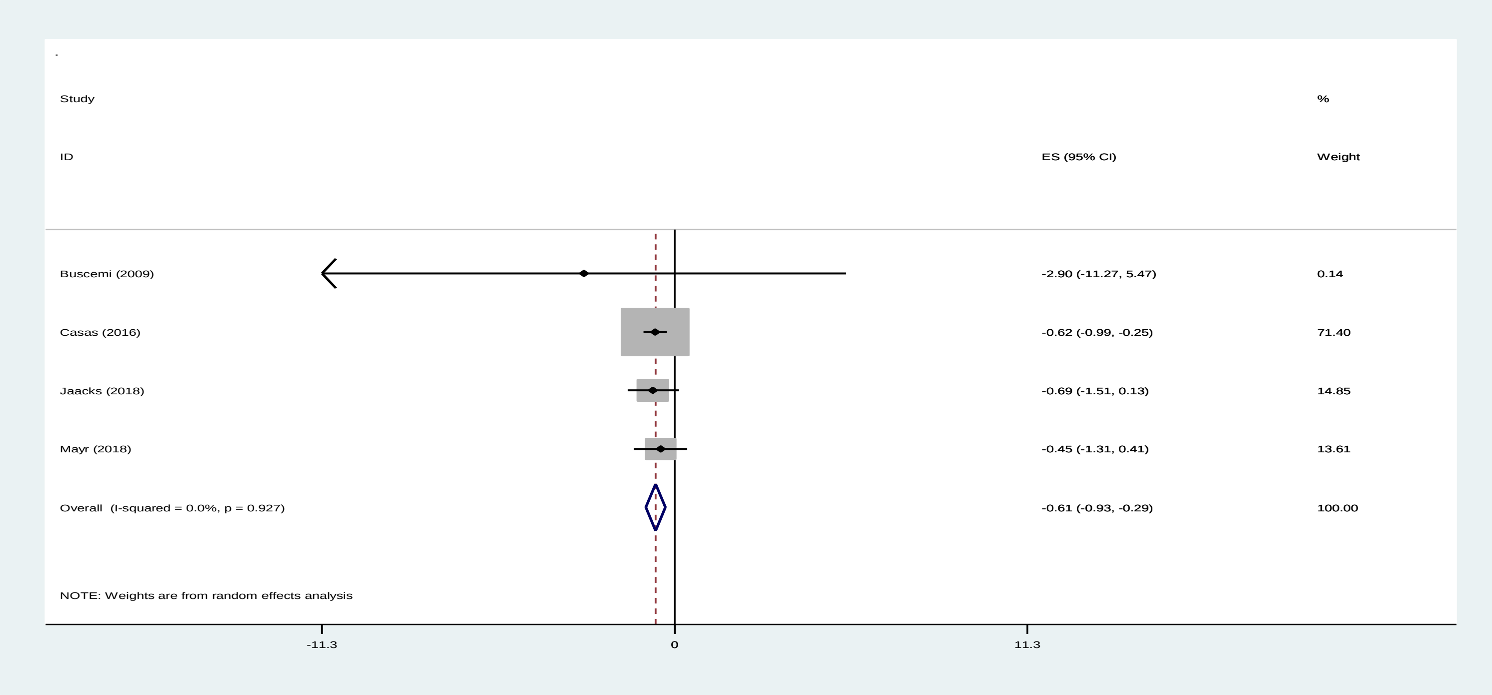

Mean differences were pooled using random effects meta-analysis. Squares indicate effect size for each study (mean difference between intervention and control group), with extended lines representing 95% confidence intervals (95% CIs). Diamonds indicate the overall weighted mean effect size. I^2 indicates between-study heterogeneity.
Detailed study characteristics can be found in Table 1 (main manuscript).

Figure S25: **Forest plot for overall blood interleukin-6**

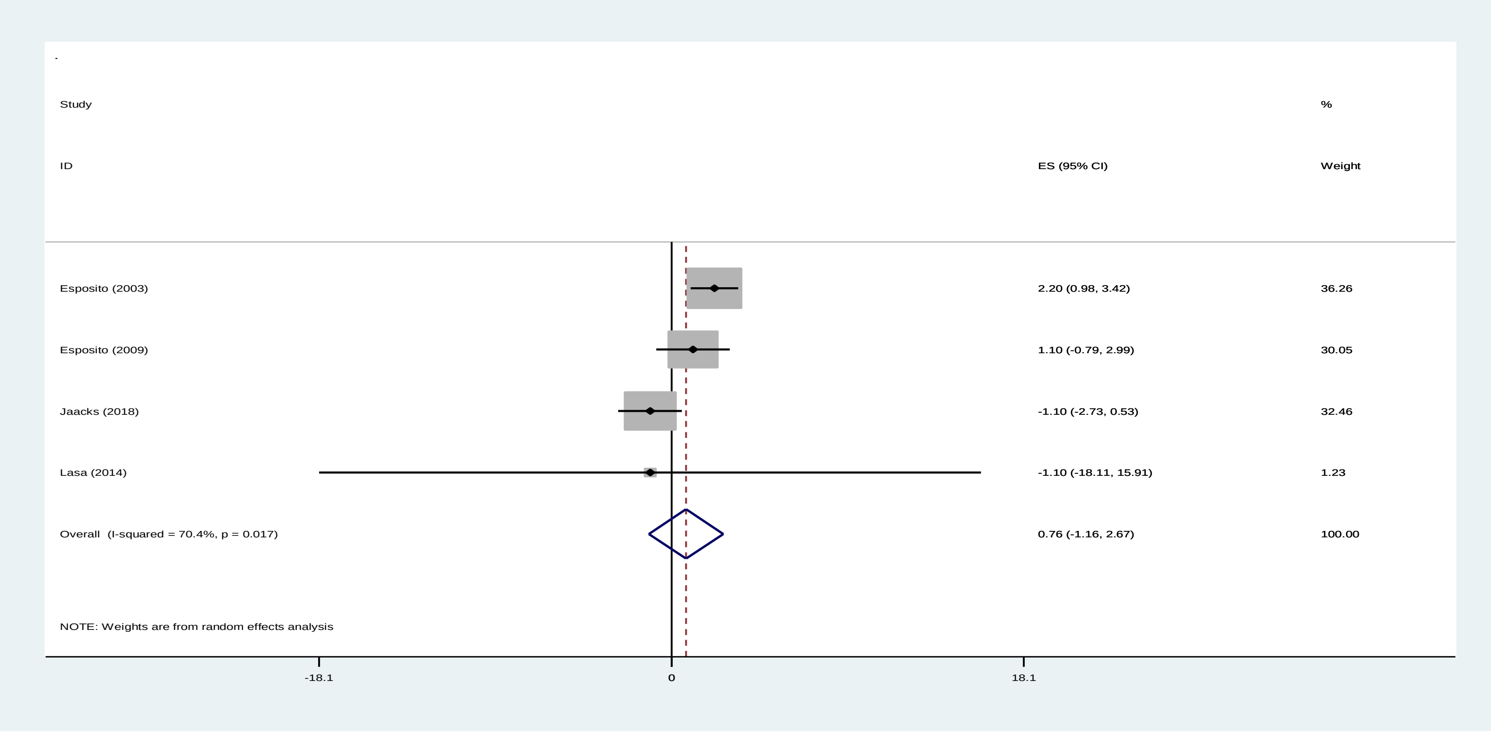

Mean differences were pooled using random effects meta-analysis. Squares indicate effect size for each study (mean difference between intervention and control group), with extended lines representing 95% confidence intervals (95% CIs). Diamonds indicate the overall weighted mean effect size. I^2 indicates between-study heterogeneity.
Detailed study characteristics can be found in Table 1 (main manuscript).

Figure S26: **Forest plot for overall blood adiponectin**

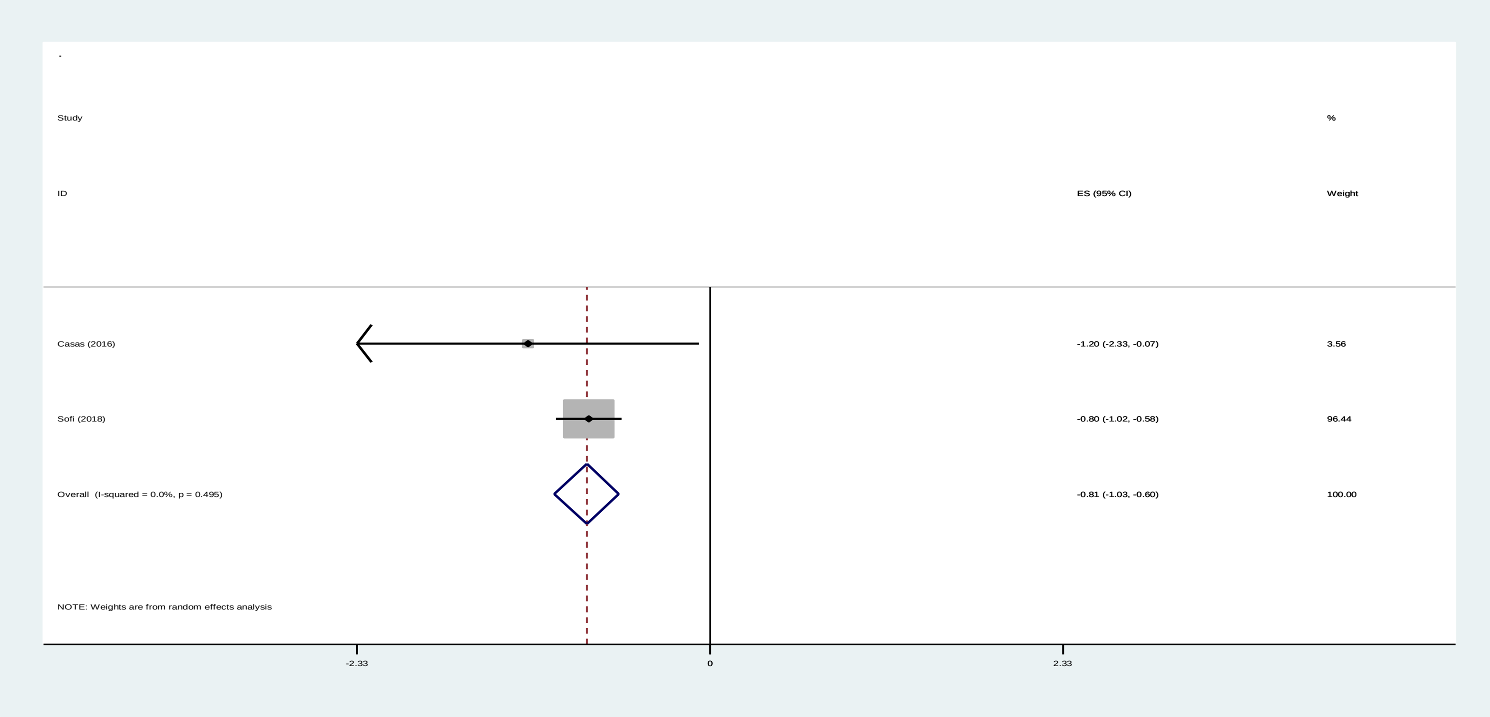

Mean differences were pooled using random effects meta-analysis. Squares indicate effect size for each study (mean difference between intervention and control group), with extended lines representing 95% confidence intervals (95% CIs). Diamonds indicate the overall weighted mean effect size. I^2 indicates between-study heterogeneity.
Detailed study characteristics can be found in Table 1 (main manuscript).

Figure S27: **Forest plot for overall blood tumour necrosis factor-a**

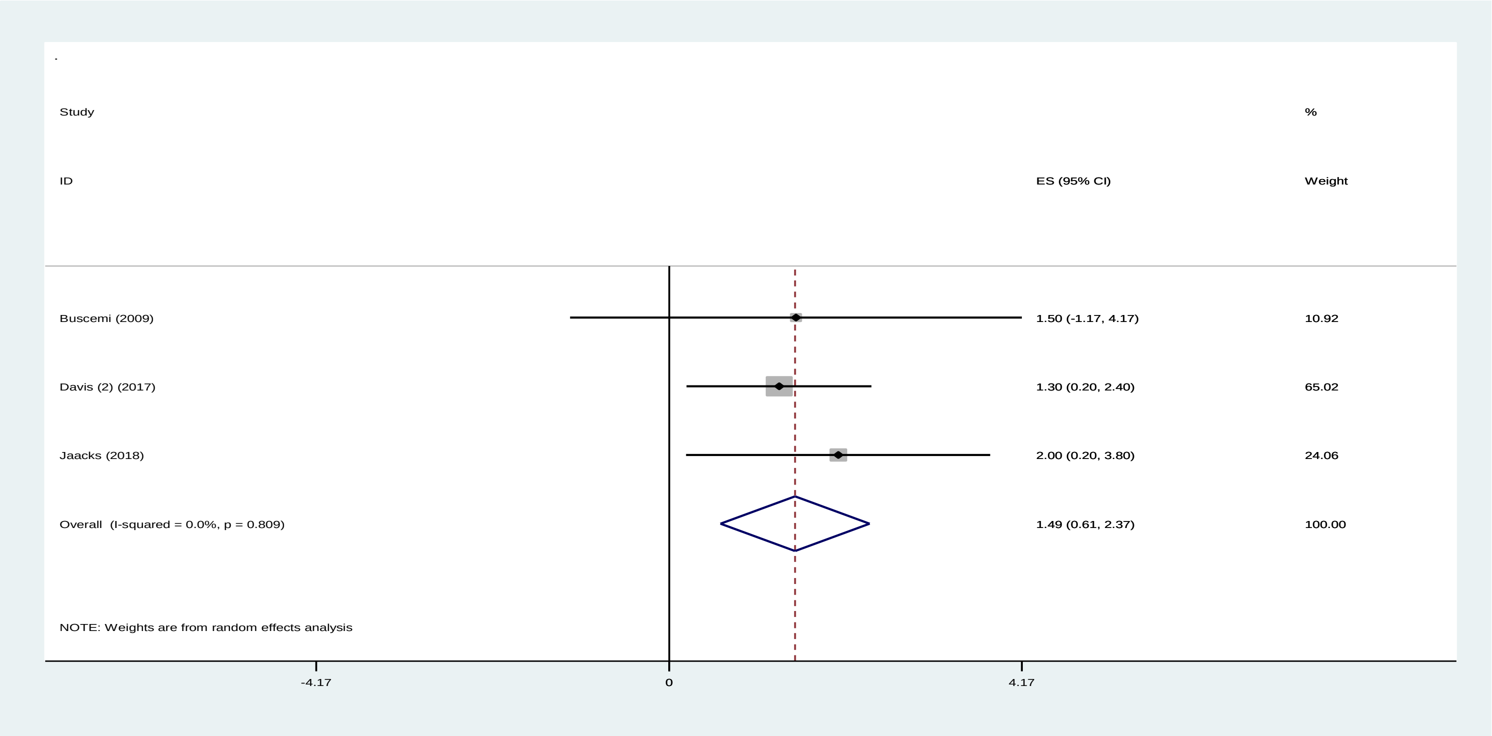

Mean differences were pooled using random effects meta-analysis. Squares indicate effect size for each study (mean difference between intervention and control group), with extended lines representing 95% confidence intervals (95% CIs). Diamonds indicate the overall weighted mean effect size. I^2 indicates between-study heterogeneity.
Detailed study characteristics can be found in Table 1 (main manuscript).

Figure S28: **Forest plot for overall flow mediated dilatation**

Supplementary Figures S29–S36. Forest plots of controlled trials evaluating the effect of the Mediterranean diet on metabolic syndrome-related comorbidities

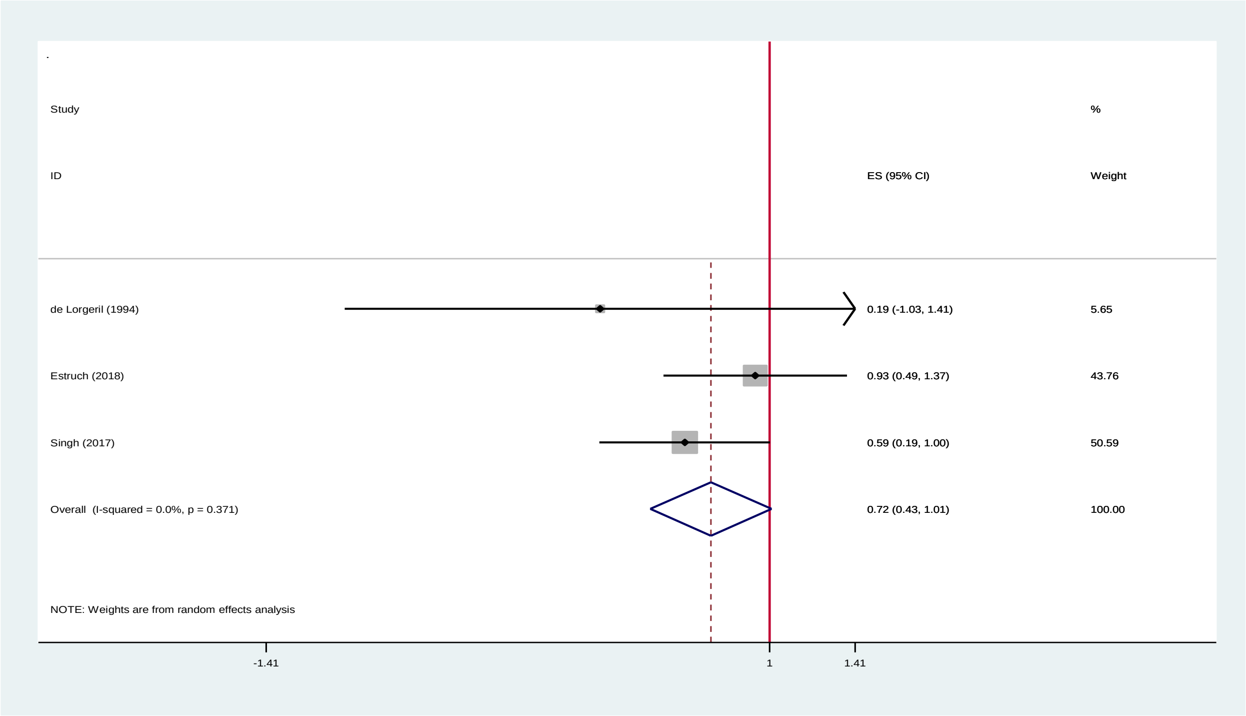

Risk ratios were calculated and pooled using random effects meta-analysis. Squares indicate relative risk of the Mediterranean diet compared to control treatment, with extended lines representing 95% confidence intervals (95% CIs). Diamonds indicate the overall weighted mean relative risk. I^2 indicates between-study heterogeneity.
Detailed study characteristics can be found in Table 1 (main manuscript).

Figure S29: **Forest plot for cardiovascular disease mortality risk**

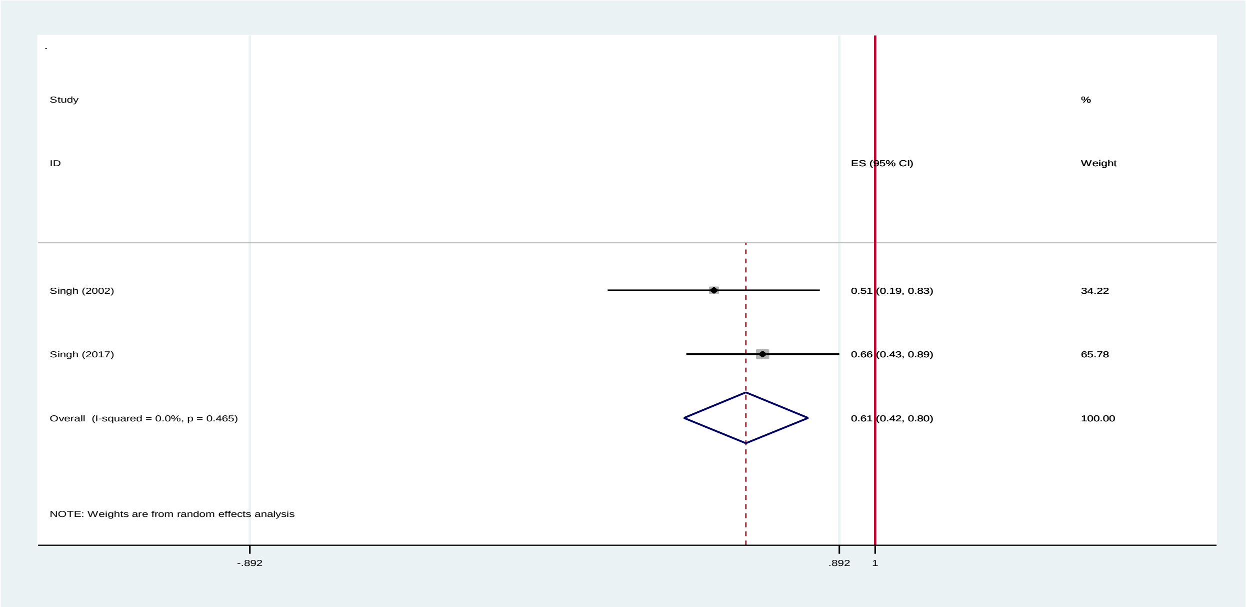

Risk ratios were calculated and pooled using random effects meta-analysis. Squares indicate relative risk of the Mediterranean diet compared to control treatment, with extended lines representing 95% confidence intervals (95% CIs). Diamonds indicate the overall weighted mean relative risk. I^2 indicates between-study heterogeneity.
Detailed study characteristics can be found in Table 1 (main manuscript).

Figure S30: **Forest plot for cardiovascular disease incidence risk**

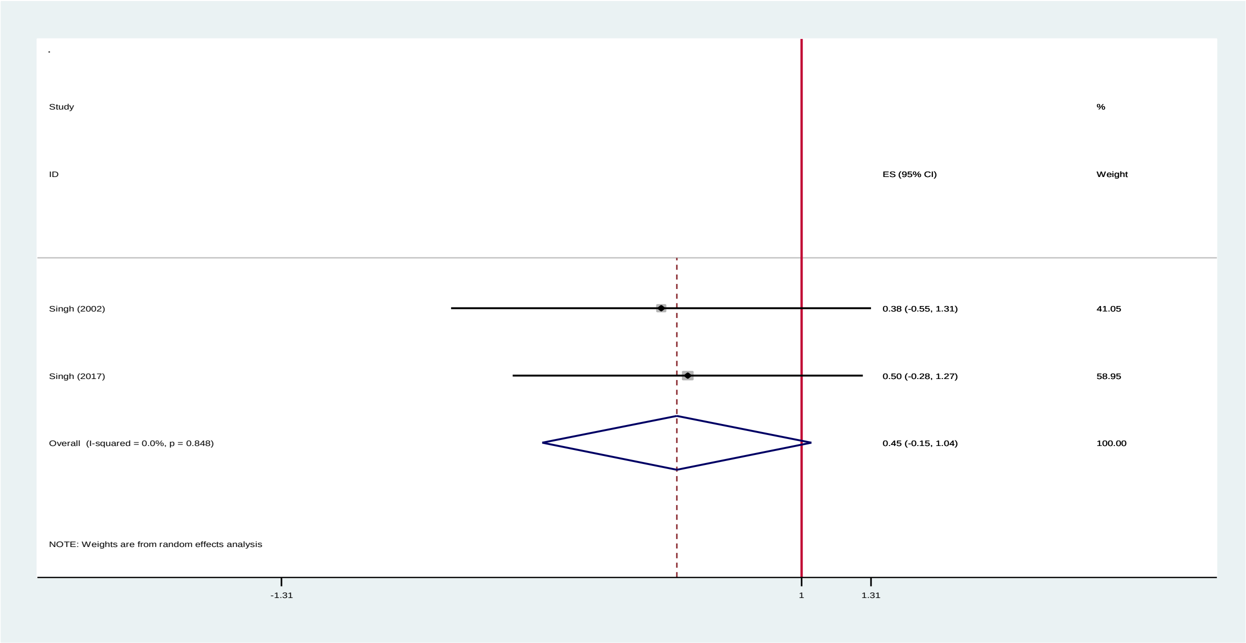

Risk ratios were calculated and pooled using random effects meta-analysis. Squares indicate relative risk of the Mediterranean diet compared to control treatment, with extended lines representing 95% confidence intervals (95% CIs). Diamonds indicate the overall weighted mean relative risk. I^2 indicates between-study heterogeneity.
Detailed study characteristics can be found in Table 1 (main manuscript).

Figure S31: **Forest plot for sudden cardiac death risk**

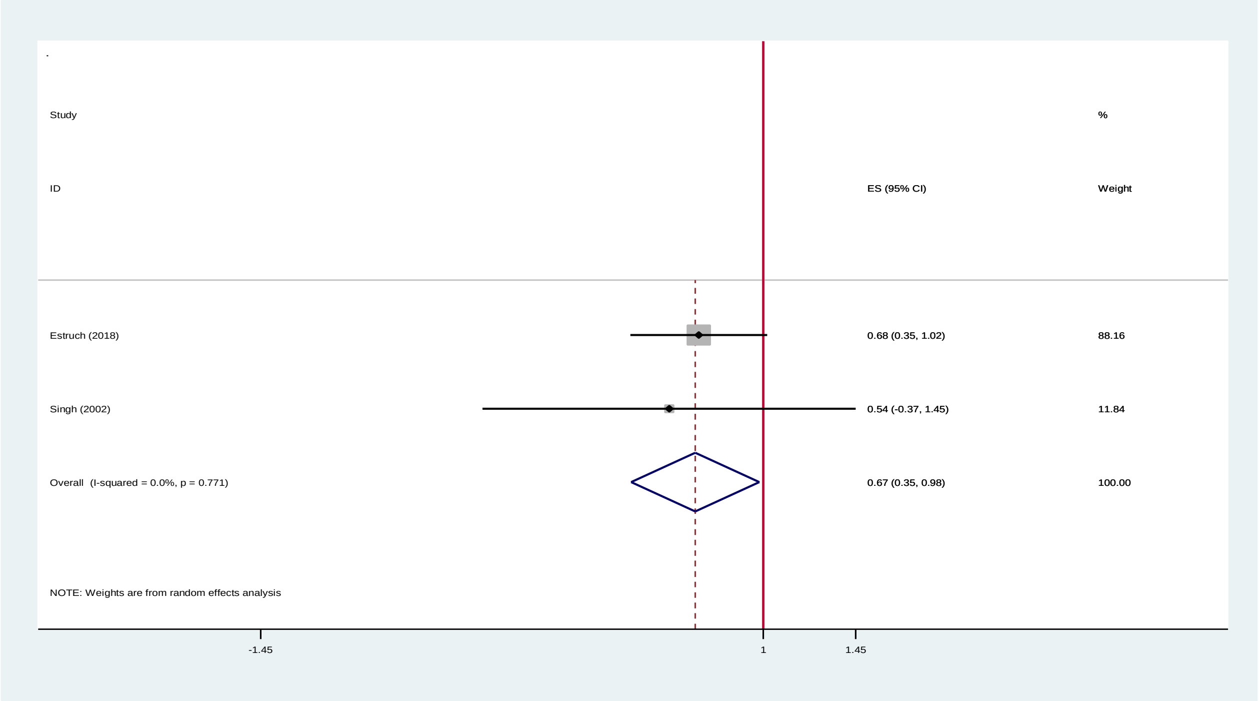

Risk ratios were calculated and pooled using random effects meta-analysis. Squares indicate relative risk of the Mediterranean diet compared to control treatment, with extended lines representing 95% confidence intervals (95% CIs). Diamonds indicate the overall weighted mean relative risk. I^2 indicates between-study heterogeneity.
Detailed study characteristics can be found in Table 1 (main manuscript).

Figure S32: **Forest plot for stroke incidence risk**

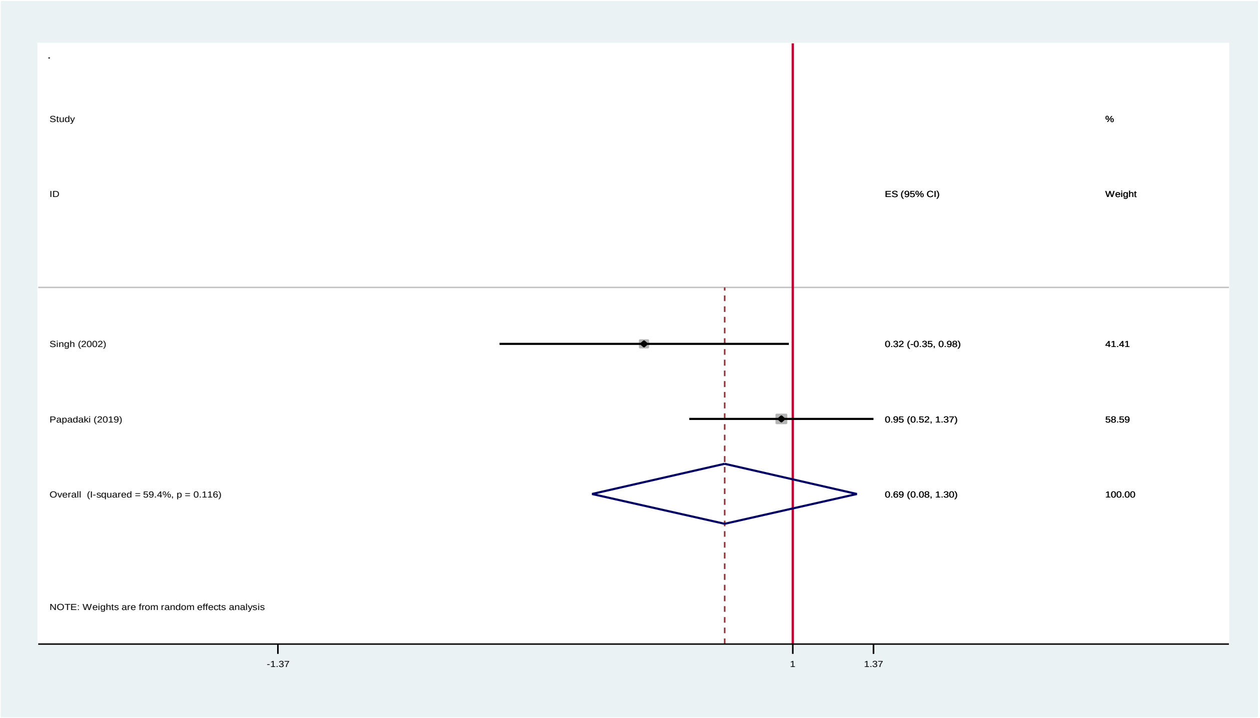

Risk ratios were calculated and pooled using random effects meta-analysis. Squares indicate relative risk of the Mediterranean diet compared to control treatment, with extended lines representing 95% confidence intervals (95% CIs). Diamonds indicate the overall weighted mean relative risk. I^2 indicates between-study heterogeneity.
Detailed study characteristics can be found in Table 1 (main manuscript).

Figure S33: **Forest plot for heart failure incidence risk**

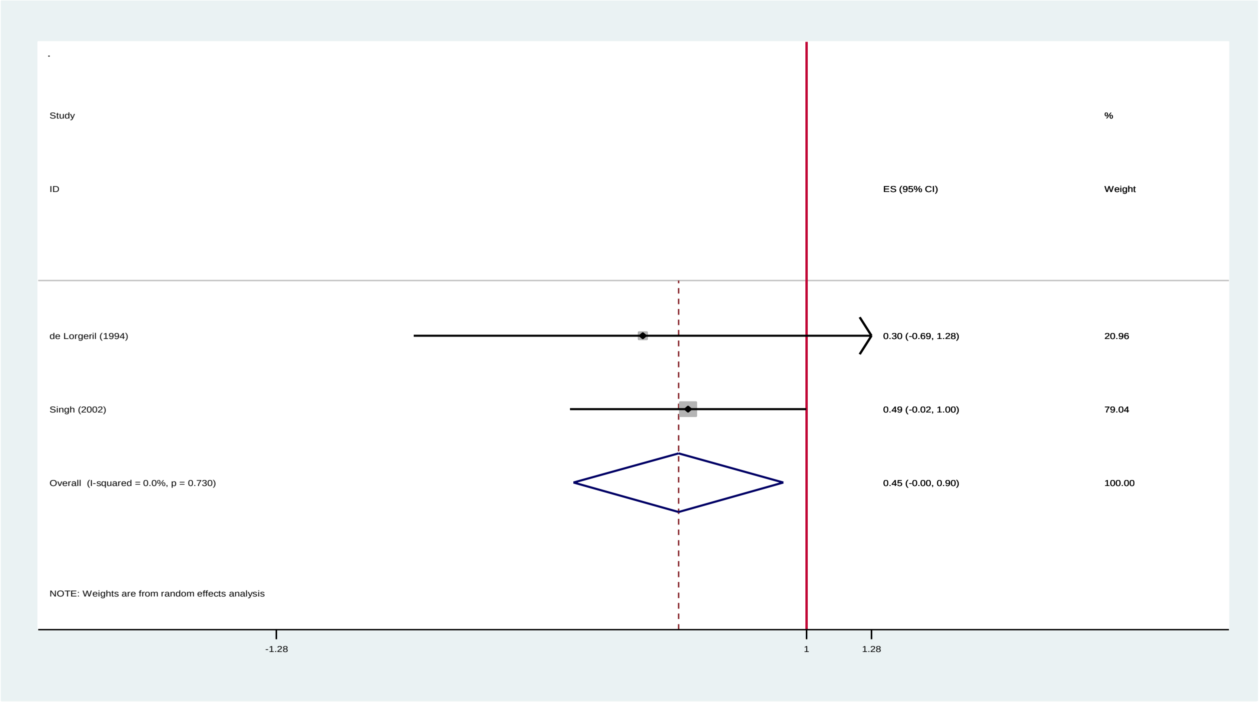

Risk ratios were calculated and pooled using random effects meta-analysis. Squares indicate relative risk of the Mediterranean diet compared to control treatment, with extended lines representing 95% confidence intervals (95% CIs). Diamonds indicate the overall weighted mean relative risk. I^2 indicates between-study heterogeneity.
Detailed study characteristics can be found in Table 1 (main manuscript).

Figure S34: **Forest plot for non-fatal myocardial infarction risk**

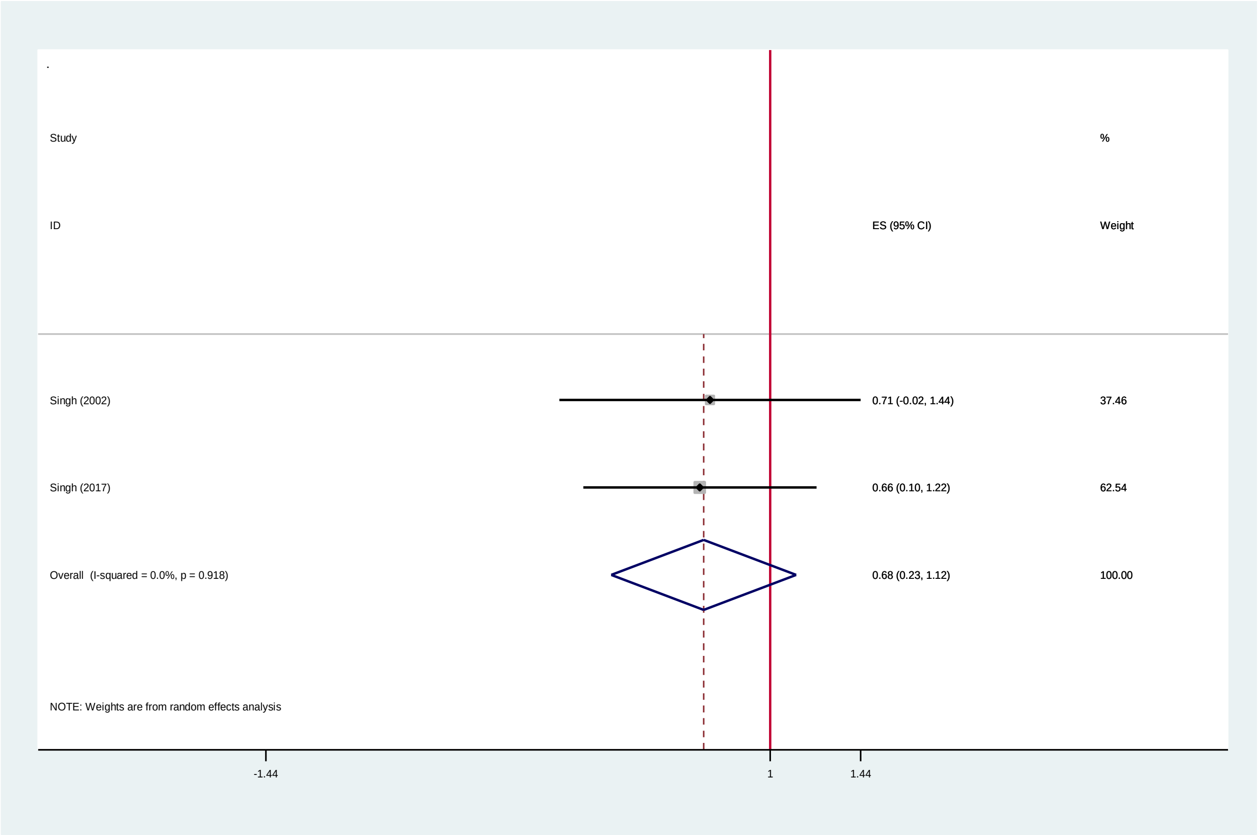

Risk ratios were calculated and pooled using random effects meta-analysis. Squares indicate relative risk of the Mediterranean diet compared to control treatment, with extended lines representing 95% confidence intervals (95% CIs). Diamonds indicate the overall weighted mean relative risk. I^2 indicates between-study heterogeneity.
Detailed study characteristics can be found in Table 1 (main manuscript).

Figure S35: **Forest plot for fatal myocardial infarction risk**

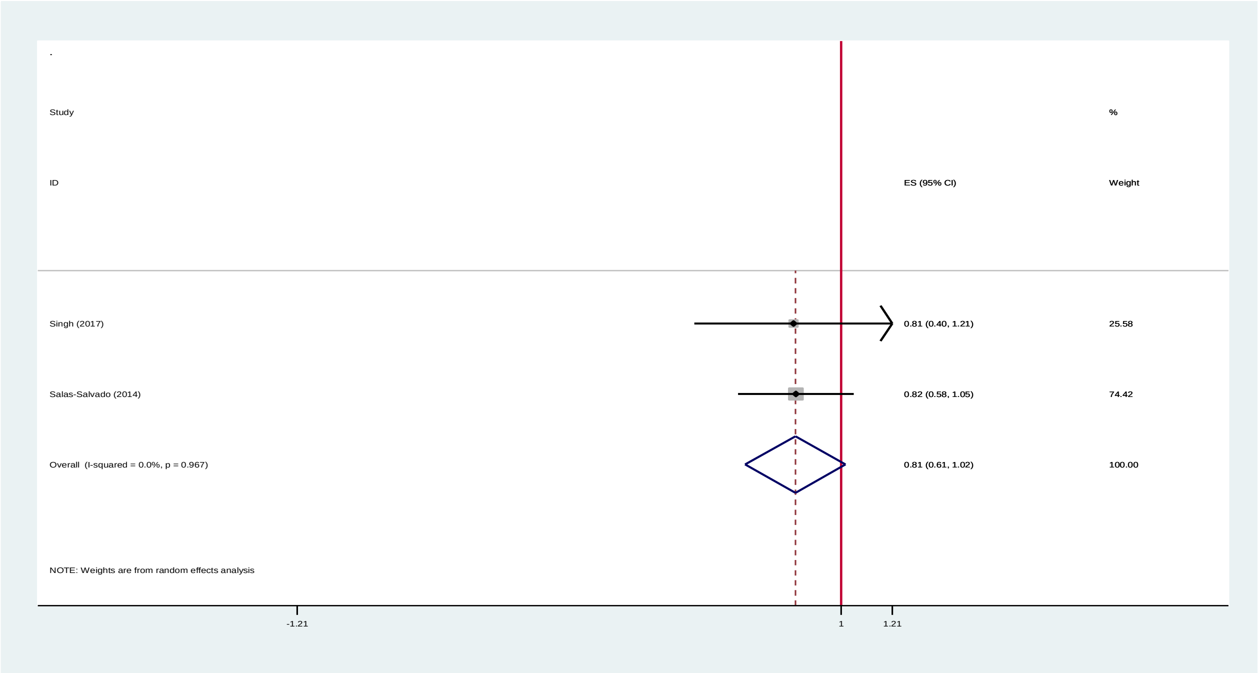

Risk ratios were calculated and pooled using random effects meta-analysis. Squares indicate relative risk of the Mediterranean diet compared to control treatment, with extended lines representing 95% confidence intervals (95% CIs). Diamonds indicate the overall weighted mean relative risk. I^2 indicates between-study heterogeneity.
Detailed study characteristics can be found in Table 1 (main manuscript).

Figure S36: **Forest plot for type 2 diabetes incidence**

Supplementary Table S10. Summary of the findings on metabolic syndrome-related comorbidities (between-group differences) from the papers (and/or outcomes) not included in the pooled analysis

	Stroke	Angina Pectoris	MI	Pre-diabetes incidence
Estruch et al. 2018 [53]	-	-	Incidence:<br>MD (EVOO) vs. CG: HR, 0.82 [CI 0.52 to 1.30]<br>MD (nuts) vs. CG: HR, 0.76 [CI 0.47 to 1.25]<br>MD (combined) vs. CG: HR, 0.80 [CI 0.53 to 1.21]	-
Singh et al. 2002 [93][*]	Mortality: 0.4 vs. 0.6%, P=0.650	Incidence: 7 vs. 11%, P=0.0133	-	-
Singh et al. 2017 [94][*]	-	-	-	-21.5 vs. 8.5%, P<0.001

	Cancer	Breast cancer incidence
Singh et al. 2002 [93][*]	Incidence: 0.4 vs. 0.4%, P=1.000<br>Mortality: 0.3 vs. 0.2%, P=1.000	-
Toledo et al. 2015 [104]	-	MD (EVOO) vs. CG: HR, 0.38 [CI 0.16 to 0.87]; P=0.020<br>MD (nuts) vs. CG: HR, 0.62 [CI 0.29 to 1.36]; P=0.240<br>MD (combined) vs. CG: HR, 0.49 [CI 0.25 to 0.94]

CG, control group; CI, confidence intervals; EVOO, extra virgin olive oil; HR, hazard ratio; MD, Mediterranean diet; MI, myocardial infarction; T2D, type 2 diabetes.
[*] Percentage comparison data refer to between-group differences in proportion of participants at post-intervention.

Supplementary Figures S37–S41. Forest plots of controlled trials evaluating the effect of the Mediterranean diet on metabolic syndrome and/or related comorbidity treatment

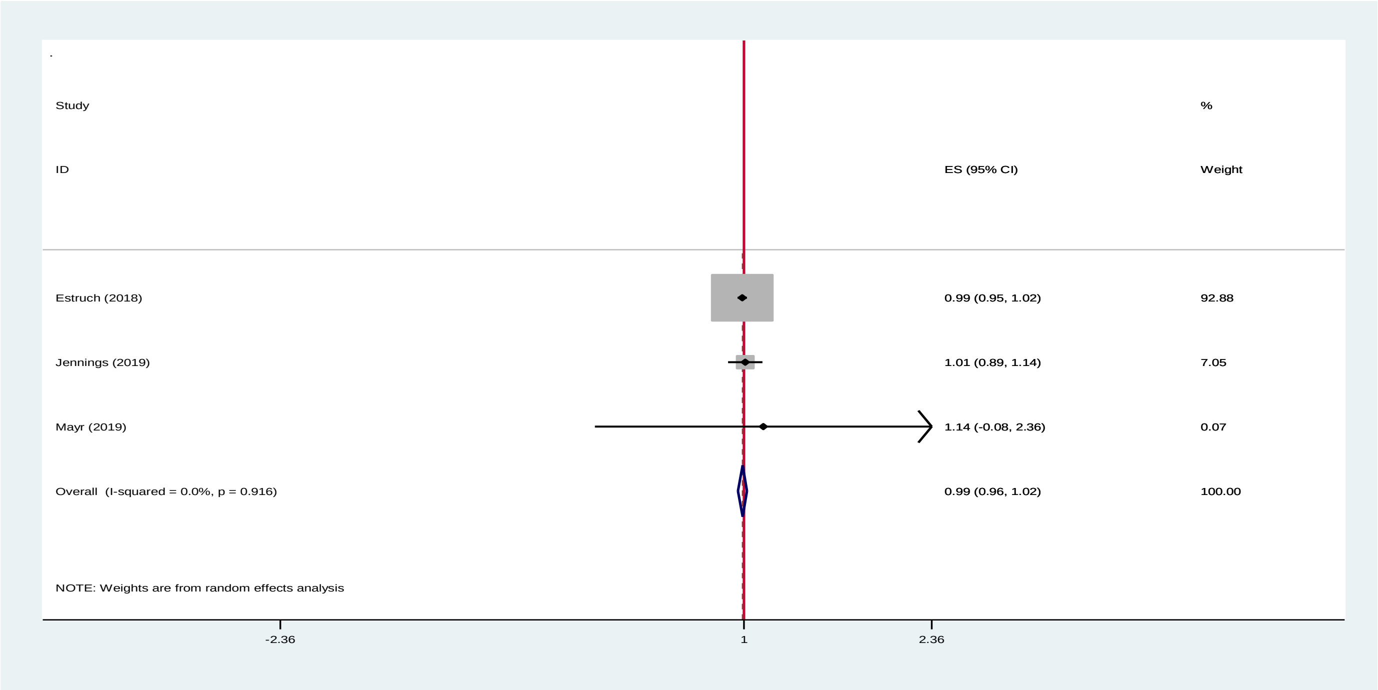

Risk ratios were calculated and pooled using random effects meta-analysis. Squares indicate relative risk of the Mediterranean diet compared to control treatment, with extended lines representing 95% confidence intervals (95% CIs). Diamonds indicate the overall weighted mean relative risk. I^2 indicates between-study heterogeneity.
Detailed study characteristics can be found in Table 1 (main manuscript).

Figure S37: **Forest plot for use of blood pressure lowering drugs**

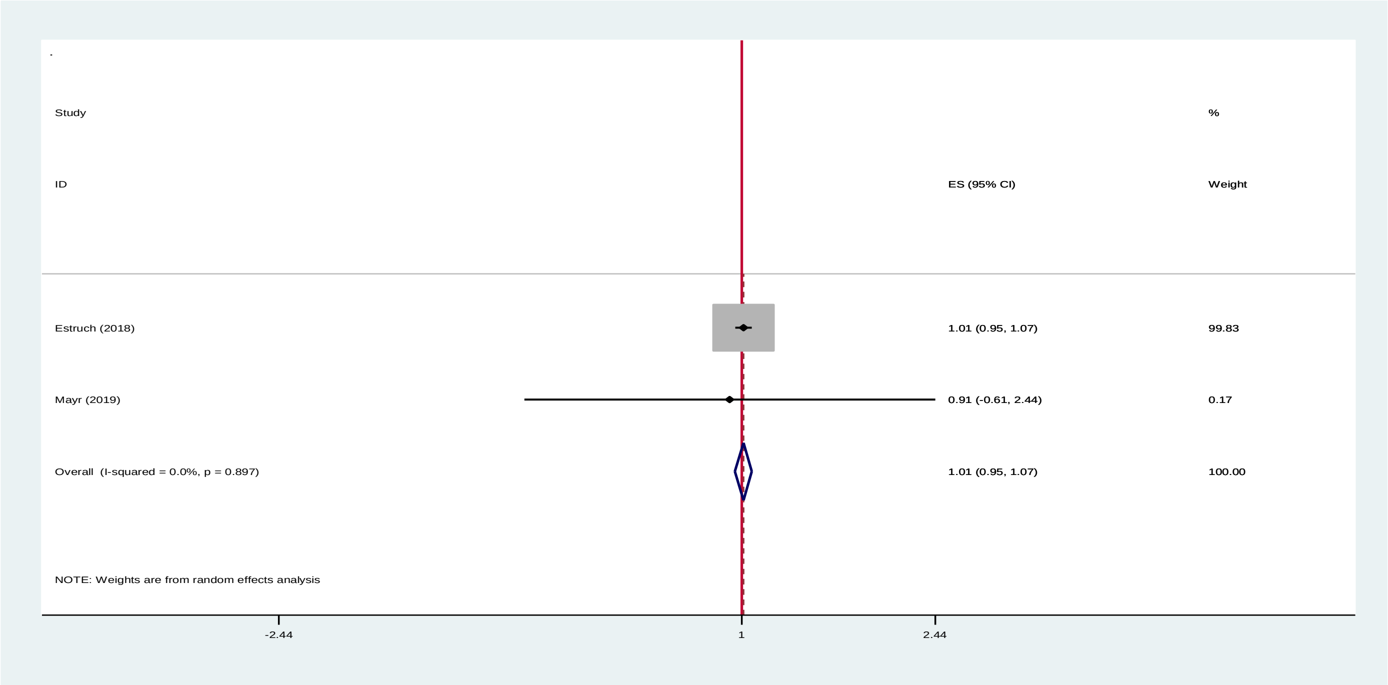

Risk ratios were calculated and pooled using random effects meta-analysis. Squares indicate relative risk of the Mediterranean diet compared to control treatment, with extended lines representing 95% confidence intervals (95% CIs). Diamonds indicate the overall weighted mean relative risk. I^2 indicates between-study heterogeneity.
Detailed study characteristics can be found in Table 1 (main manuscript).

Figure S38: **Forest plot for use of lipid-lowering agents**

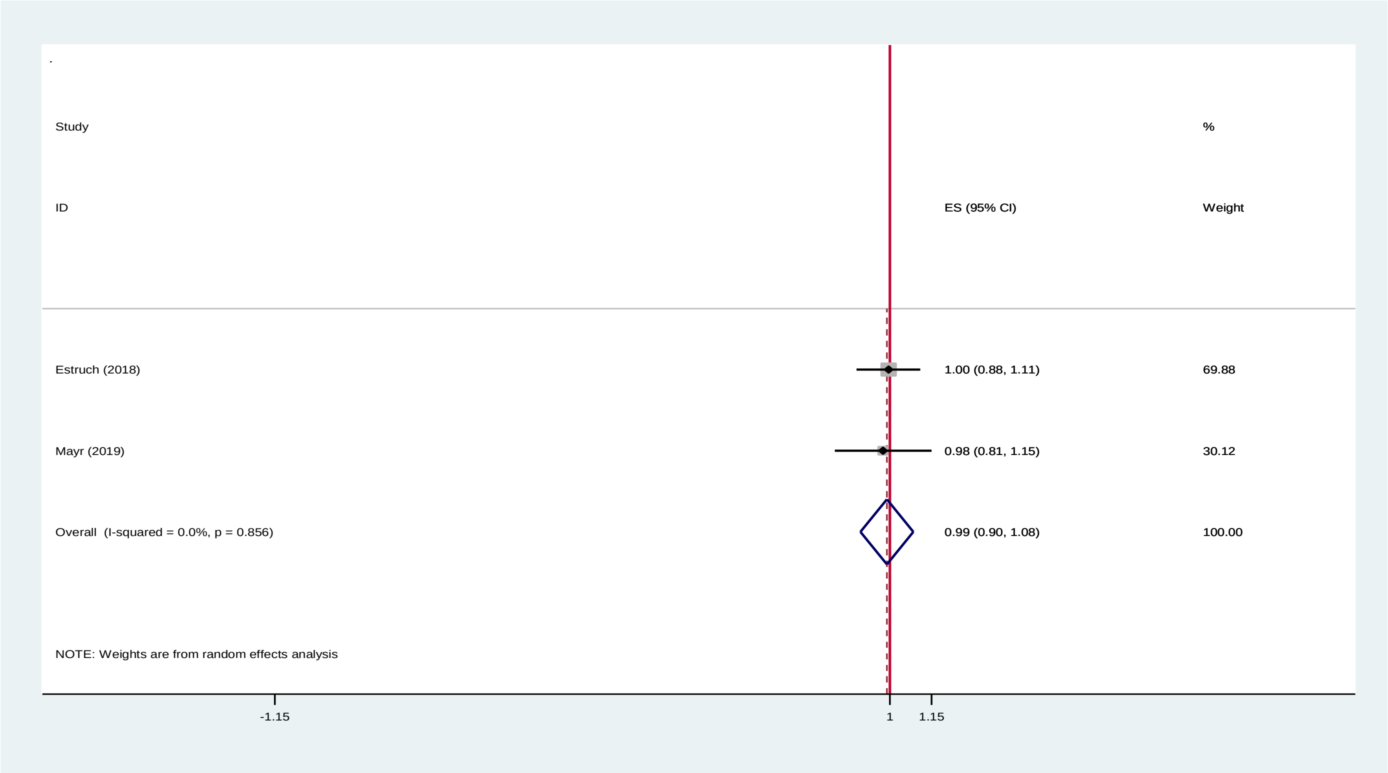

Risk ratios were calculated and pooled using random effects meta-analysis. Squares indicate relative risk of the Mediterranean diet compared to control treatment, with extended lines representing 95% confidence intervals (95% CIs). Diamonds indicate the overall weighted mean relative risk. I^2 indicates between-study heterogeneity.
Detailed study characteristics can be found in Table 1 (main manuscript).

Figure S39: **Forest plot for use of anti-platelet therapy**

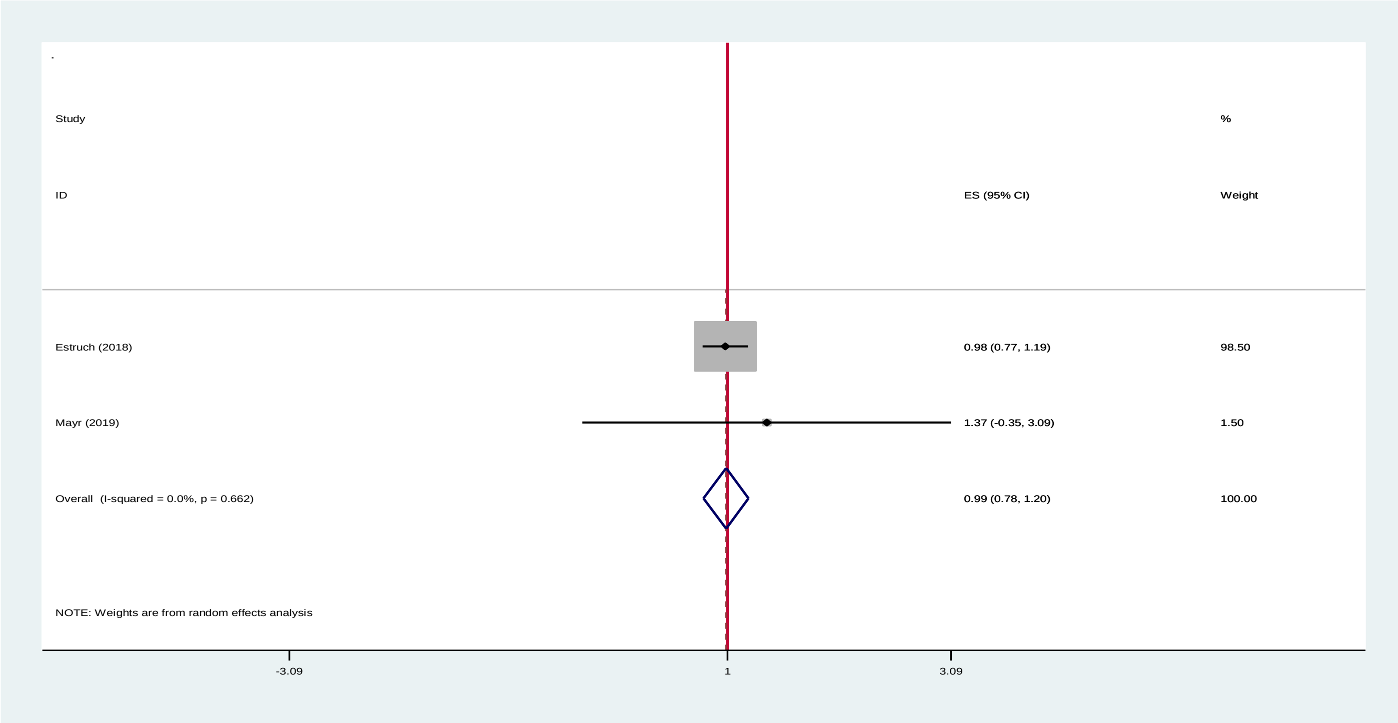

Risk ratios were calculated and pooled using random effects meta-analysis. Squares indicate relative risk of the Mediterranean diet compared to control treatment, with extended lines representing 95% confidence intervals (95% CIs). Diamonds indicate the overall weighted mean relative risk. I^2 indicates between-study heterogeneity.
Detailed study characteristics can be found in Table 1 (main manuscript).

Figure S40: **Forest plot for use of insulin**

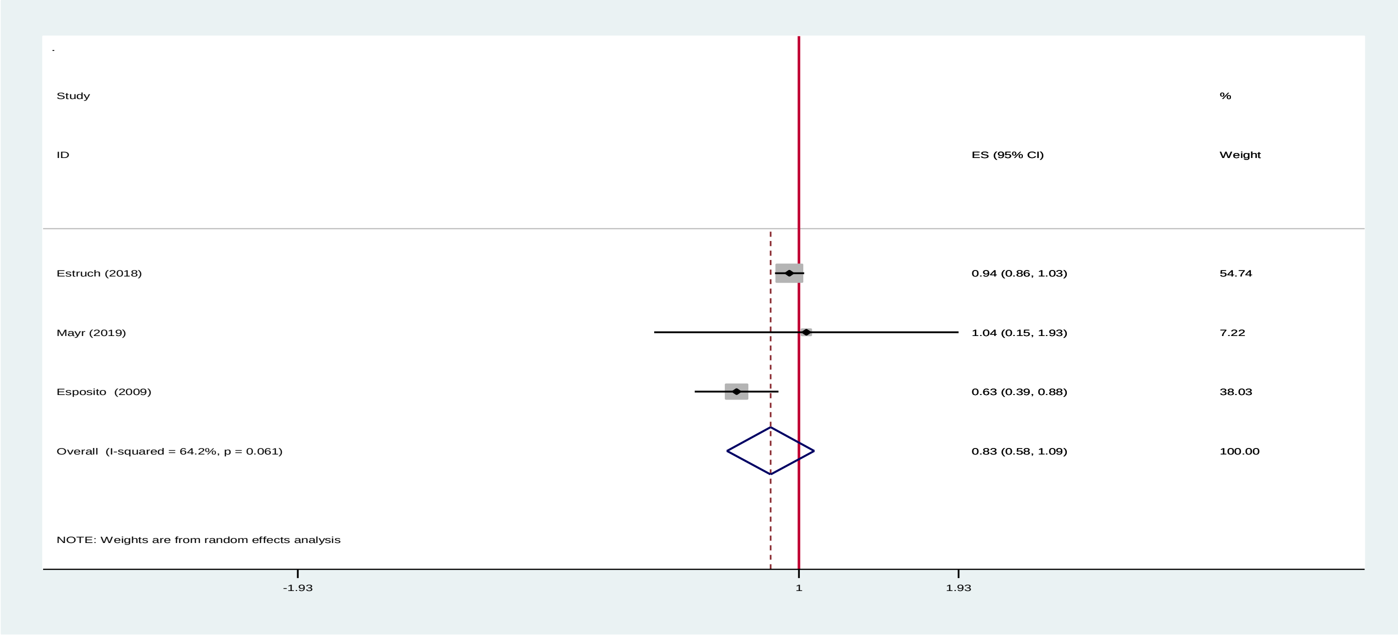

Risk ratios were calculated and pooled using random effects meta-analysis. Squares indicate relative risk of the Mediterranean diet compared to control treatment, with extended lines representing 95% confidence intervals (95% CIs). Diamonds indicate the overall weighted mean relative risk. I^2 indicates between-study heterogeneity.
Detailed study characteristics can be found in Table 1 (main manuscript).

Figure S41: **Forest plot for use of oral antidiabetic agents**

Supplementary Table S11. Summary of the findings on metabolic syndrome and/or related comorbidity treatment (between-group differences) from the papers (and/or outcomes) not included in the pooled analysis

	Need for antihyperglycemic drug therapy	Use of blood pressure-lowering drugs	Use of lipid-lowering agents	Use of nitrates	Use of verapamil (β-blocker)	Use of disopyramide (irregular heartbeat)
Esposito et al. 2014 (follow-up of Esposito et al. 2009) [48]	MD vs. CG: HR= 0.68, 95% CI 0.50-0.89; P <0.001	-	-	-	-	-
Shai et al. 2008 [91][*]	No change, no difference between groups (no data provided)	No change, no difference between groups (no data provided)	No change, no difference between groups (no data provided)	-	-	-
Singh et al. 2002 [93][*]	-	-	-	-20% vs. -7%, P<0.0001	-7% vs. -2%, P<0.001	-4% vs. 0%, P<0.0001

CG, control group; CI, confidence intervals; HR, hazard ratio; MD, Mediterranean diet.
[*] Percentage comparison data refer to between-groups changes in the proportion of participants from baseline to post-intervention

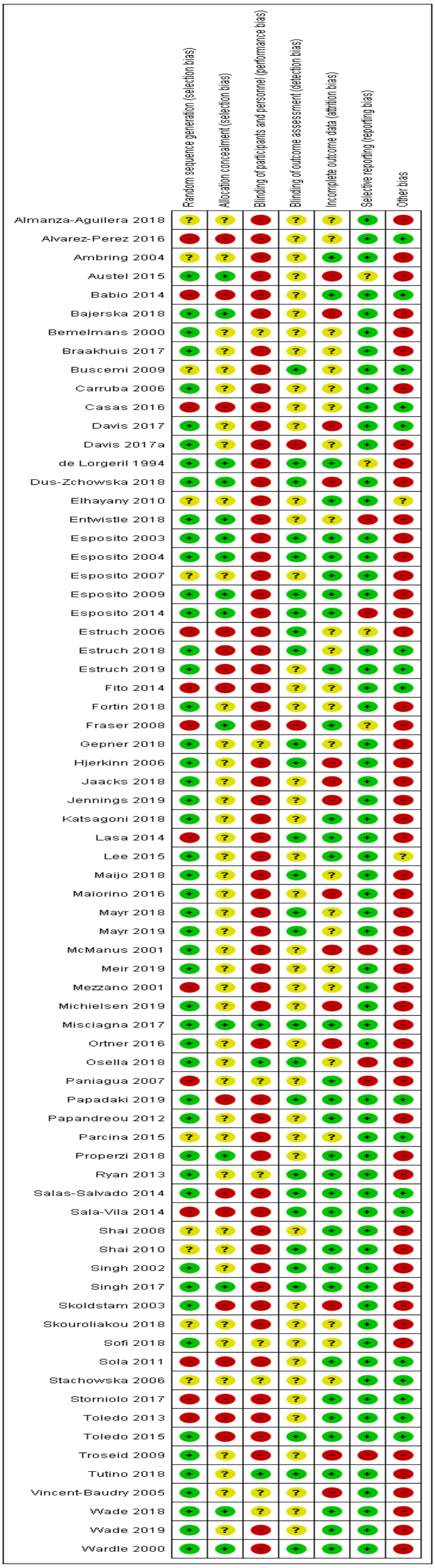

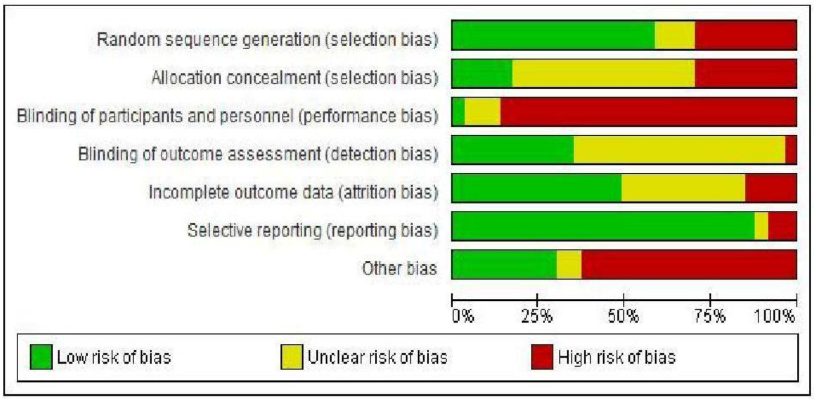

Supplementary Figure S42. Risk of bias for papers reporting a randomised controlled trial

Supplementary Figures S43–S83. Funnel plots and Egger test of studies evaluating the effect of the Mediterranean diet on anthropometric, blood pressure, biochemical, insulin resistance, oxidative stress, inflammatory and endothelial function markers related to the metabolic syndrome, metabolic syndrome-related comorbidities and metabolic syndrome and/or related comorbidity treatment (SE, standard error)

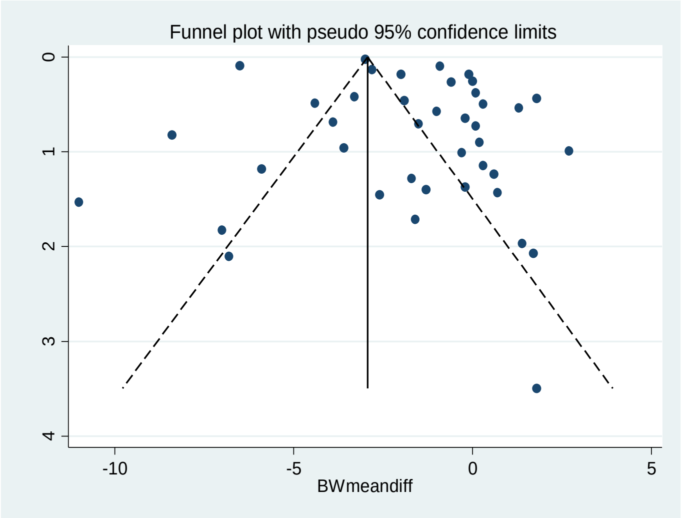

Egger's test P value= 0.112

Figure S43: **Funnel plot of effect of the Mediterranean diet on body weight**

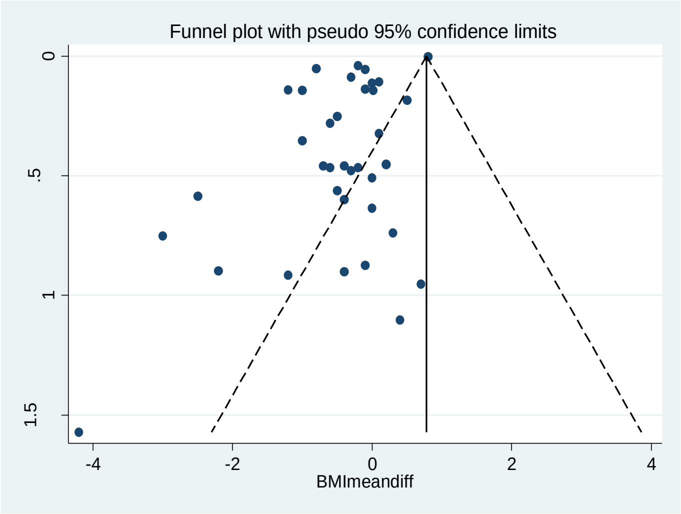

Egger's test P value= <0.001

Figure S44: **Funnel plot of effect of the Mediterranean diet on body mass index**

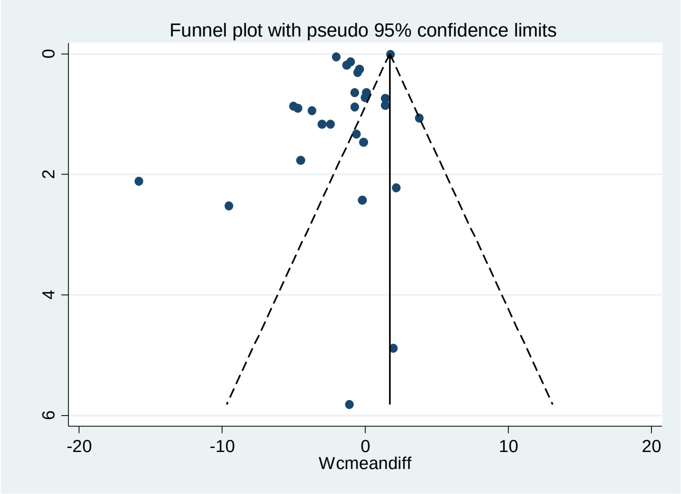

Egger's test P value= 0.026

Figure S45: **Funnel plot of effect of the Mediterranean diet on waist circumference**

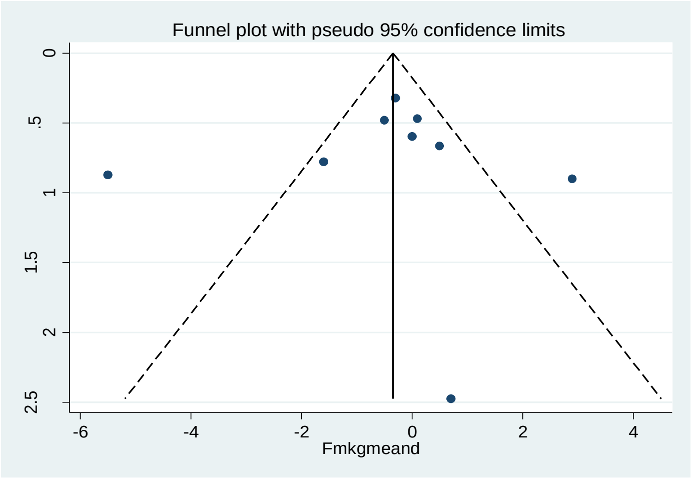

Egger’s test P value= 0.804

Figure S46: **Funnel plot of effect of the Mediterranean diet on total fat mass**

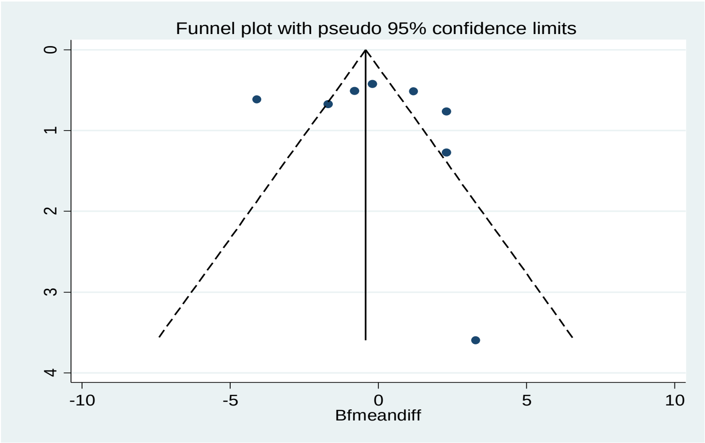

Egger’s test P value= 0.643

Figure S47: **Funnel plot of effect of the Mediterranean diet on total body fat %**

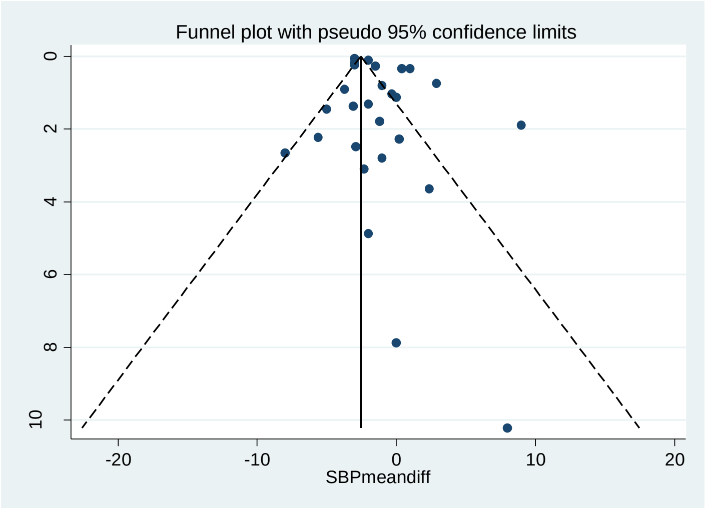

Egger’s test P value= 0.039

Figure S48: **Funnel plot of effect of the Mediterranean diet on systolic blood pressure**

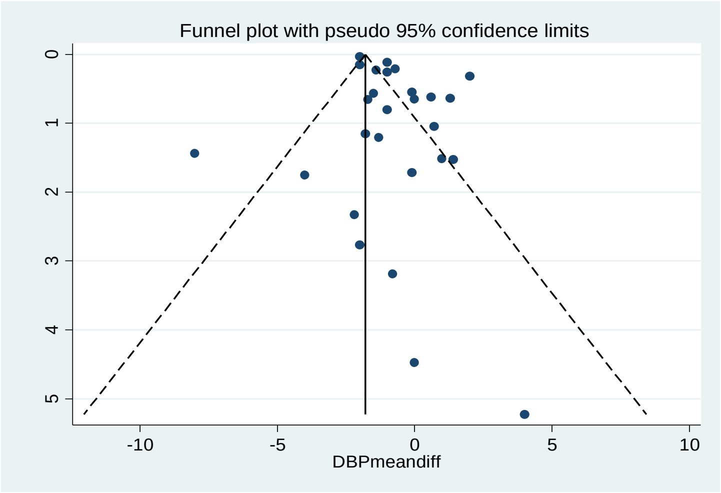

Egger's test P value= 0.011

Figure S49: **Funnel plot of effect of the Mediterranean diet on diastolic blood pressure**

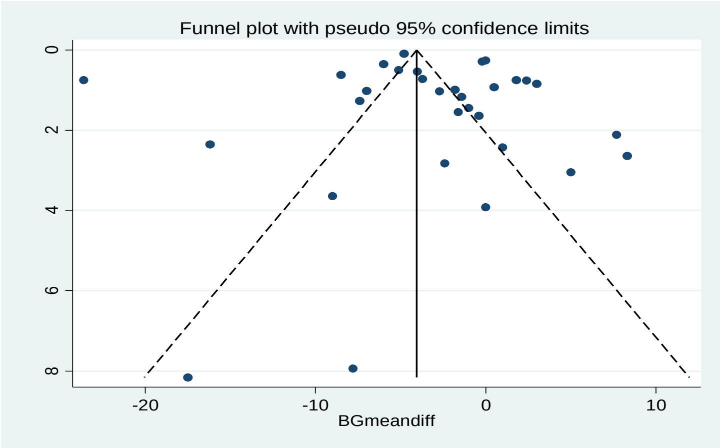

Egger's test P value= 0.464

Figure S50: **Funnel plot of effect of the Mediterranean diet on blood glucose concentrations**

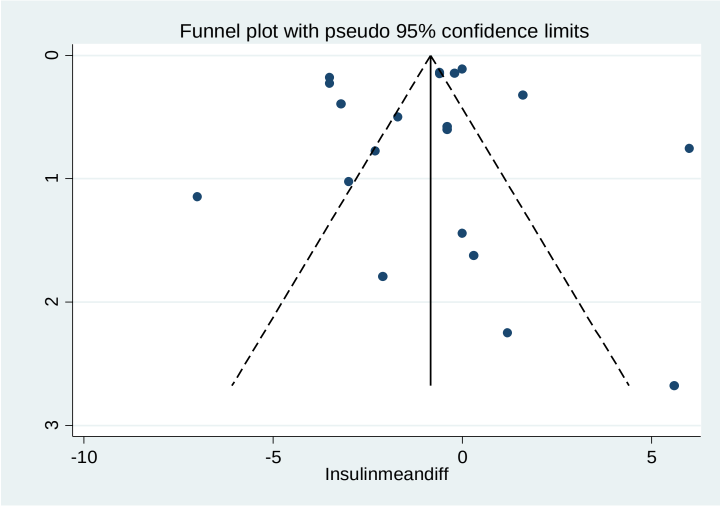

Egger's test P value= 0.737

Figure S51: **Funnel plot of effect of the Mediterranean diet on insulin concentrations**

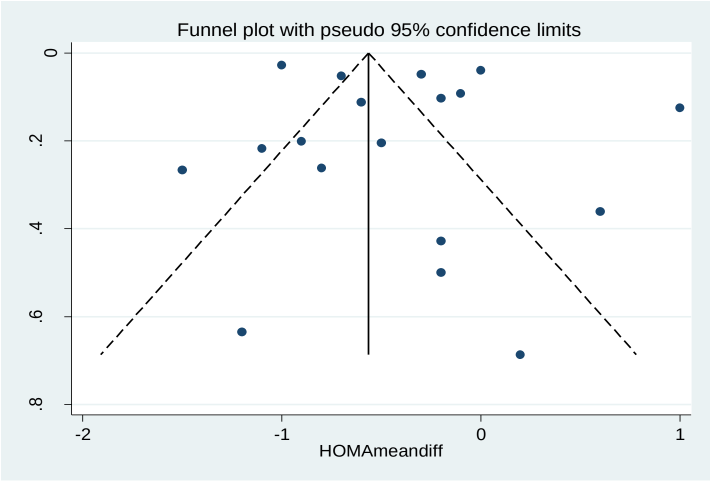

Egger’s test P value= 0.360

Figure S52: **Funnel plot of effect of the Mediterranean diet on HOMA-IR**

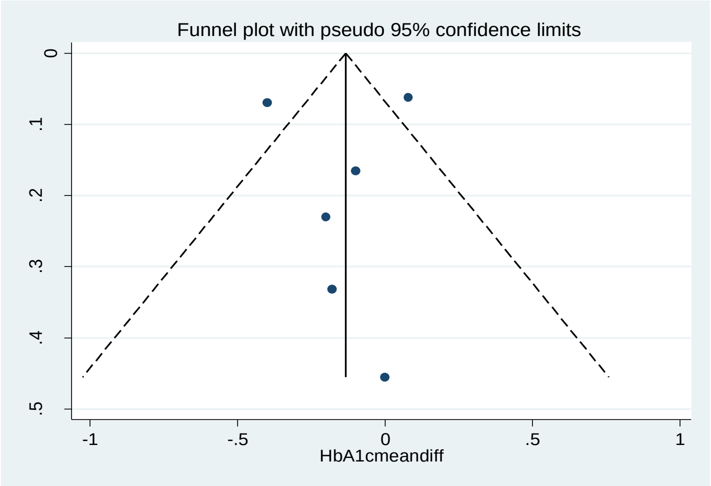

Egger’s test P value= 0.928

Figure S53: **Funnel plot of effect of the Mediterranean diet on HbA1c concentrations**

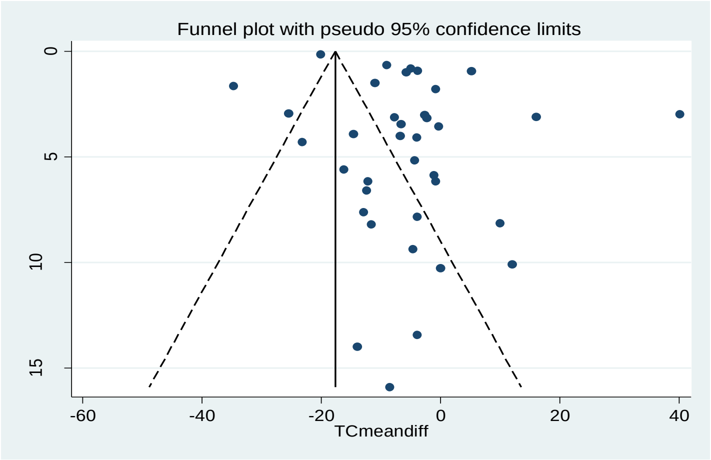

Egger’s test P value= 0.001

Figure S54: **Funnel plot of effect of the Mediterranean diet on total cholesterol concentrations**

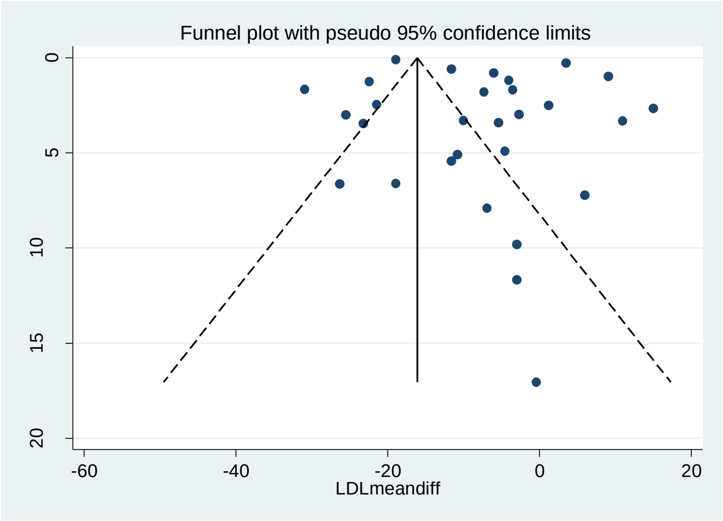

Egger's test P value= 0.089

Figure S55: **Funnel plot of effect of the Mediterranean diet on LDL-cholesterol concentrations**

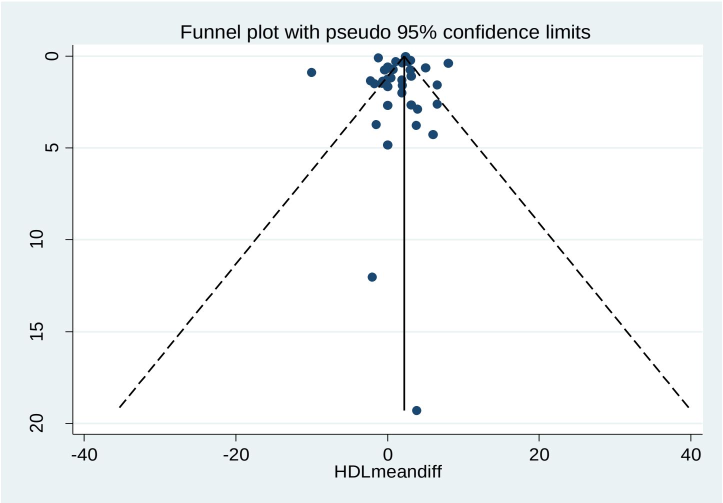

Egger's test P value= 0.326

Figure S56: **Funnel plot of effect of the Mediterranean diet on HDL-cholesterol concentrations**

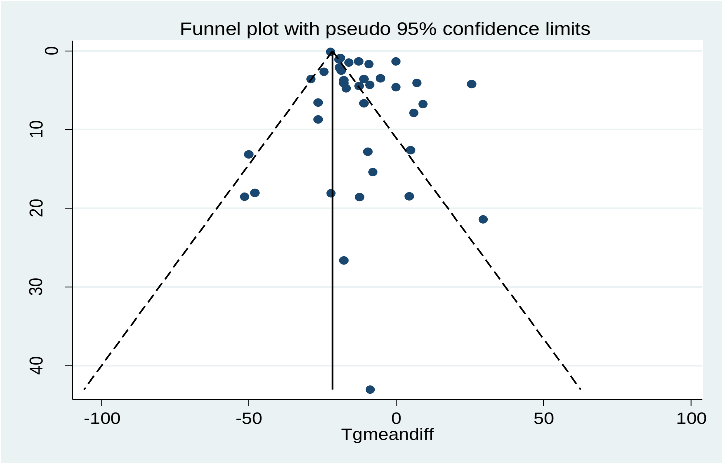

Egger's test P value= <0.001

Figure S57: **Funnel plot of effect of the Mediterranean diet on triglyceride concentrations**

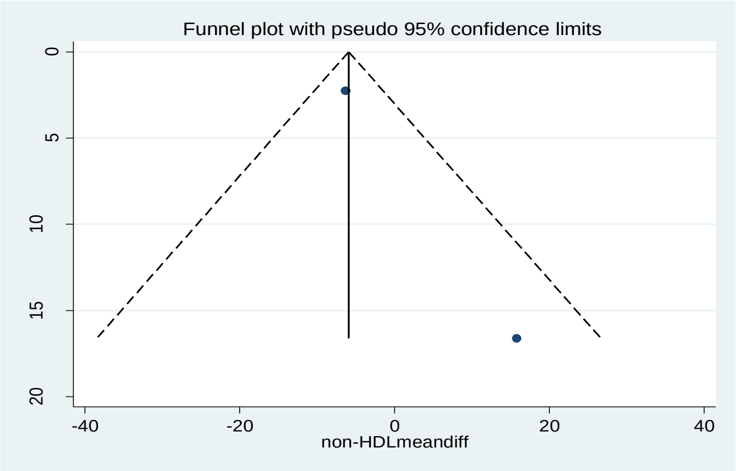

Figure S58: **Funnel plot of effect of the Mediterranean diet on non-HDL-cholesterol concentrations**

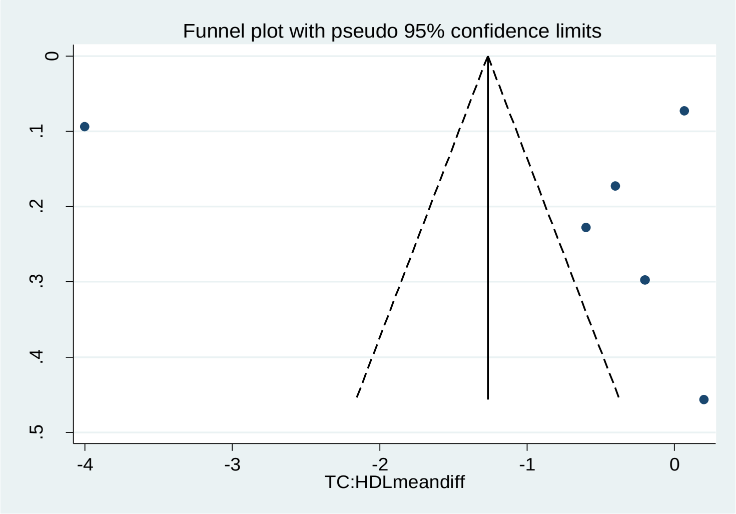

Egger's test P value= 0.872

Figure S59: **Funnel plot of effect of the Mediterranean diet on total:HDL-cholesterol ratio**

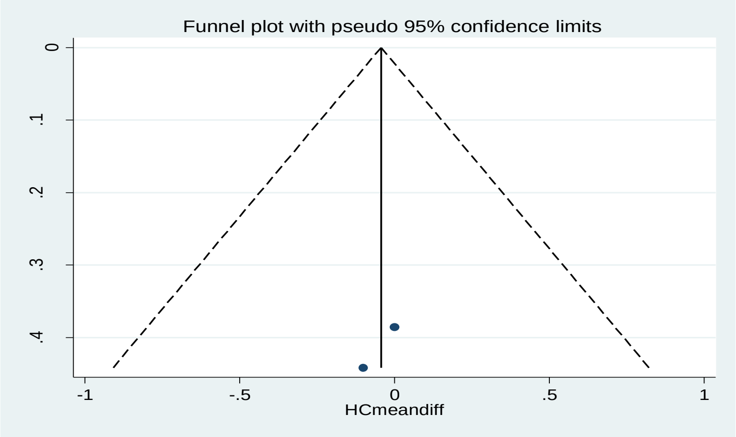

Figure S60: **Funnel plot of effect of the Mediterranean diet on homocysteine concentrations**

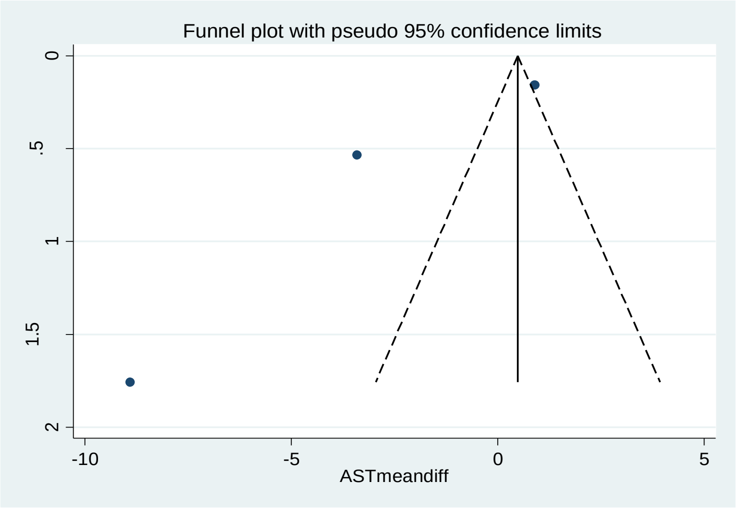

Egger's test P value= 0.200

Figure S61: **Funnel plot of effect of the Mediterranean diet on aspartame transaminase concentrations**

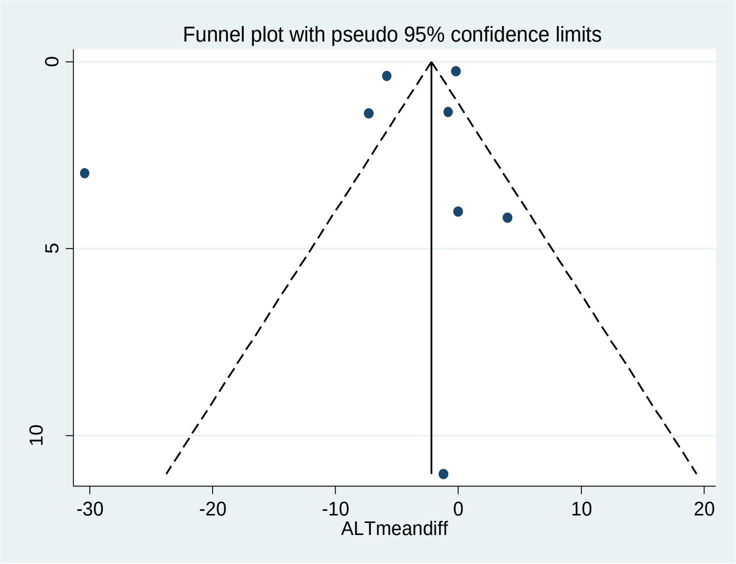

Egger's test P value= 0.402

Figure S62: **Funnel plot of effect of the Mediterranean diet on alanine transaminase concentrations**

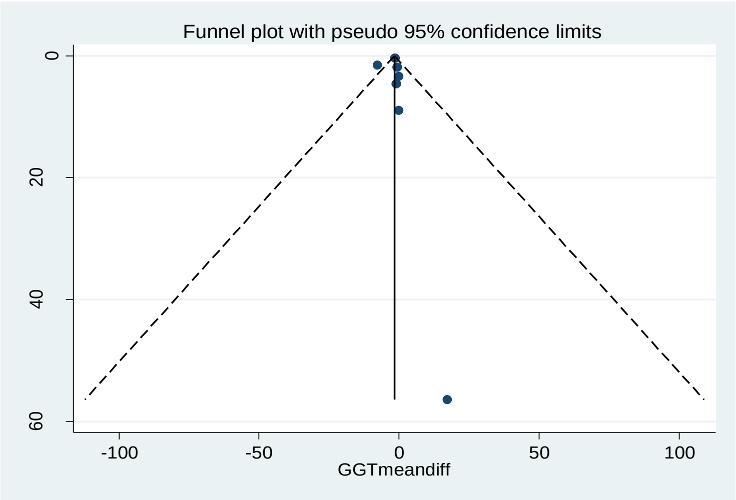

Egger's test P value= 0.700

Figure S63: **Funnel plot of effect of the Mediterranean diet on gamma glutamyl transferase concentrations**

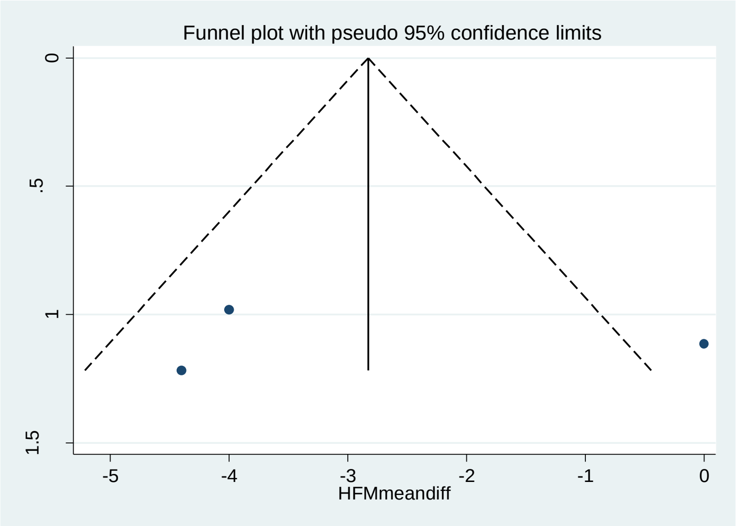

Egger's test P value= 0.927

Figure S64: **Funnel plot of effect of the Mediterranean diet on hepatic fat mass**

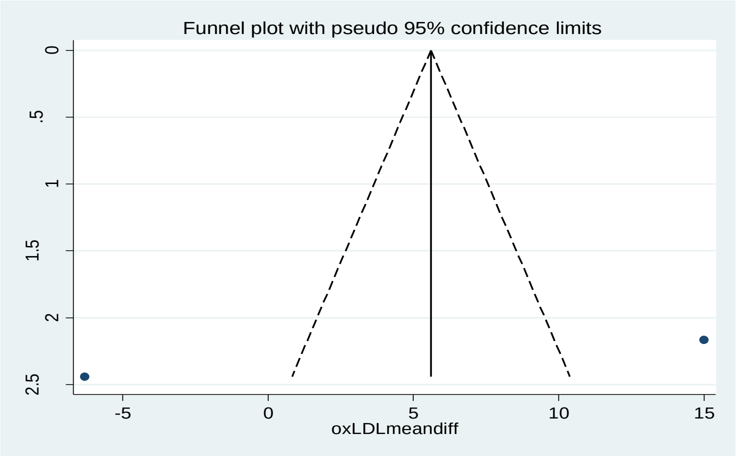

Figure S65: **Funnel plot of effect of the Mediterranean diet on oxidised LDL-cholesterol concentrations**

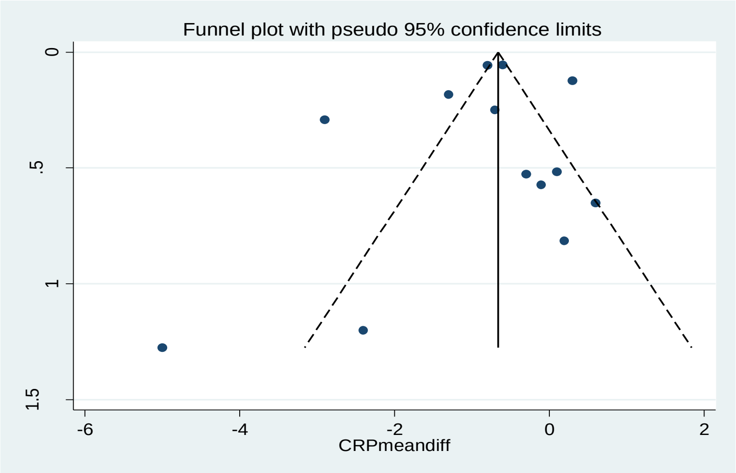

Egger's test P value= 0.728

Figure S66: **Funnel plot of effect of the Mediterranean diet on C-reactive protein concentrations**

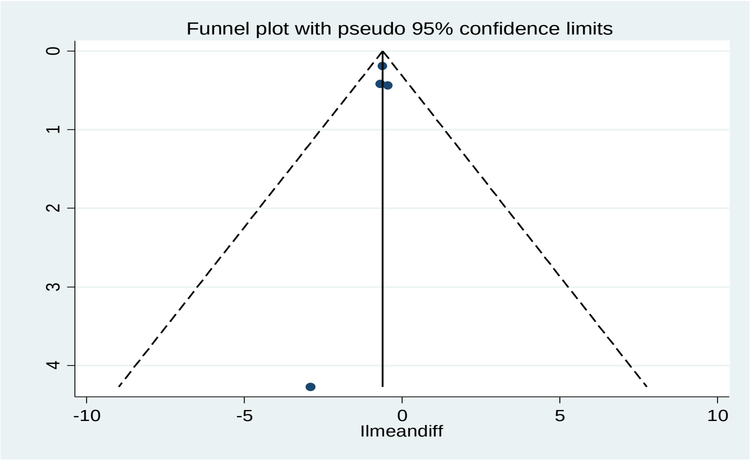

Egger’s test P value= 0.477

Figure S67: **Funnel plot of effect of the Mediterranean diet on interleukin-6 concentrations**

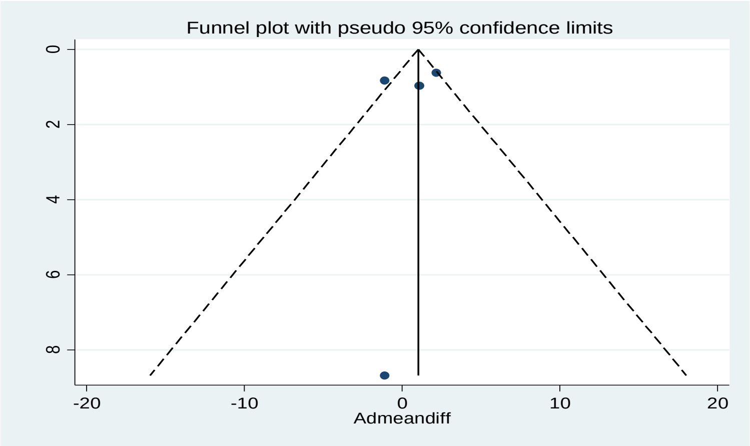

Egger’s test P value= 0.722

Figure S68: **Funnel plot of effect of the Mediterranean diet on adiponectin concentrations**

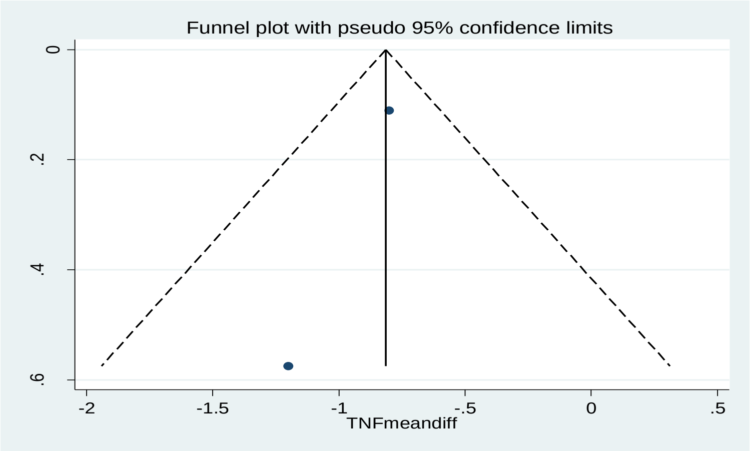

Figure S69: **Funnel plot of effect of the Mediterranean diet on tumour necrosis factor-a concentrations**

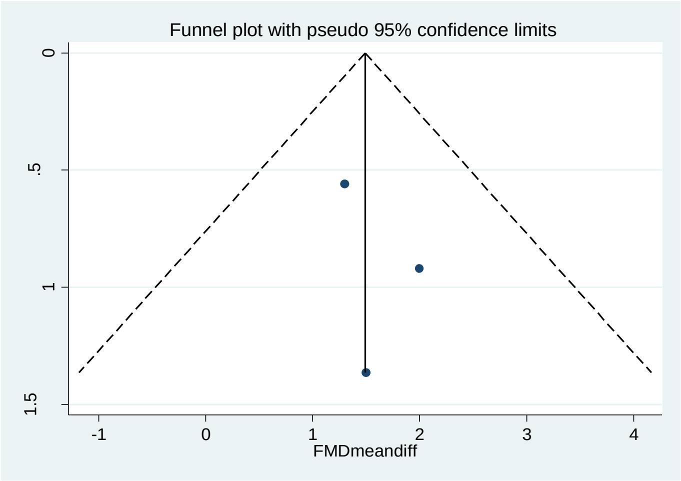

Egger's test P value= 0.611

Figure S70: **Funnel plot of effect of the Mediterranean diet on flow-mediated dilatation**

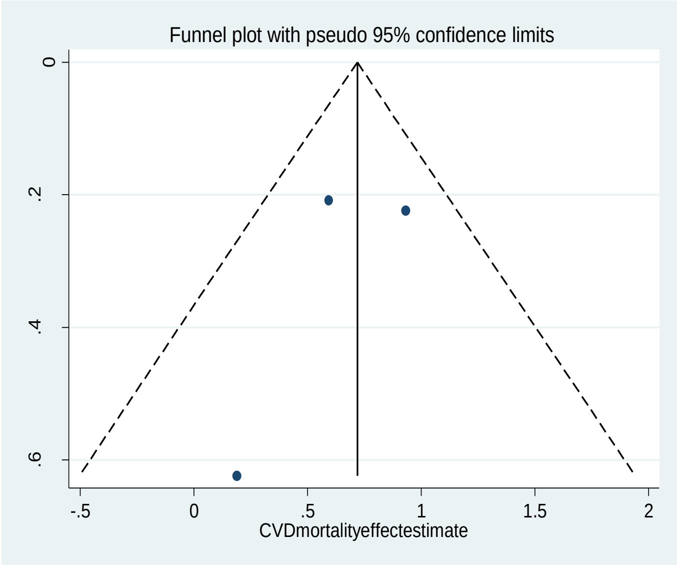

Egger's test P value= 0.624

Figure S71: **Funnel plot of effect of the Mediterranean diet on cardiovascular disease mortality**

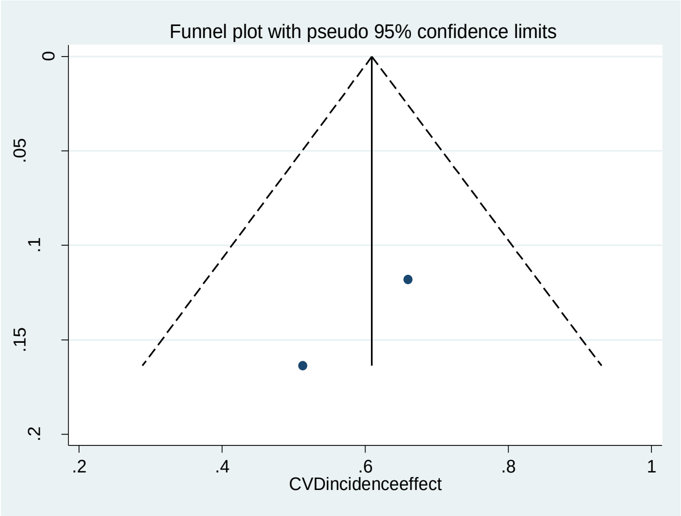

Figure S72: **Funnel plot of effect of the Mediterranean diet on cardiovascular disease incidence**

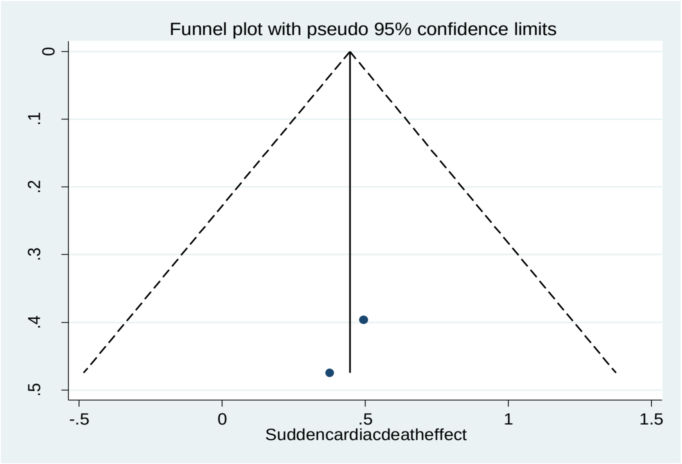

Figure S73: **Funnel plot of effect of the Mediterranean diet on sudden cardiac death**

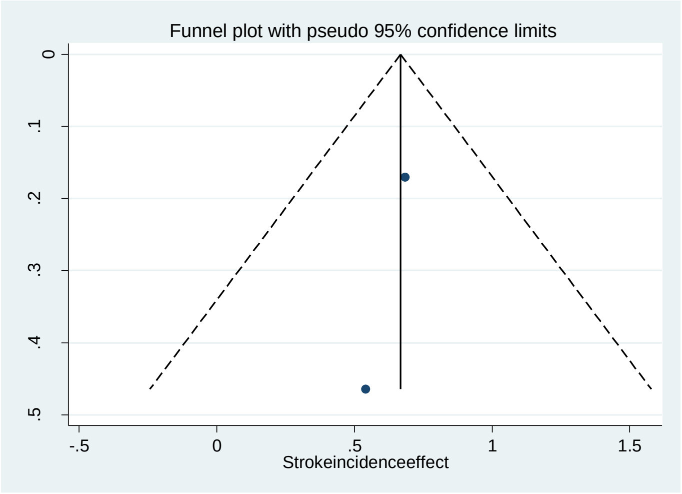

Figure S74: **Funnel plot of effect of the Mediterranean diet on stroke incidence**

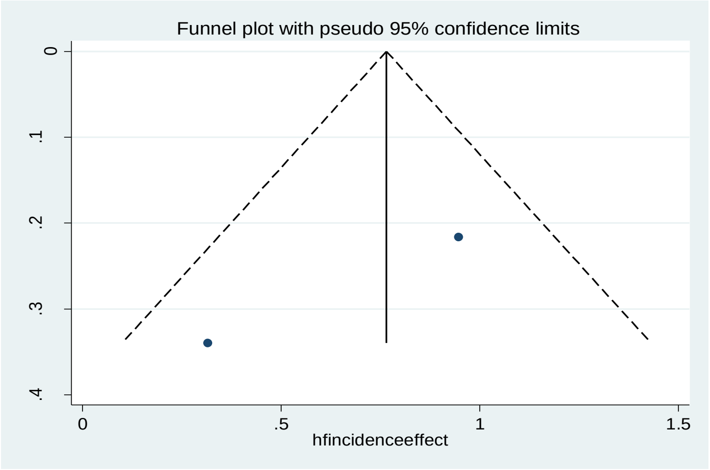

Figure S75: **Funnel plot of effect of the Mediterranean diet on heart failure incidence**

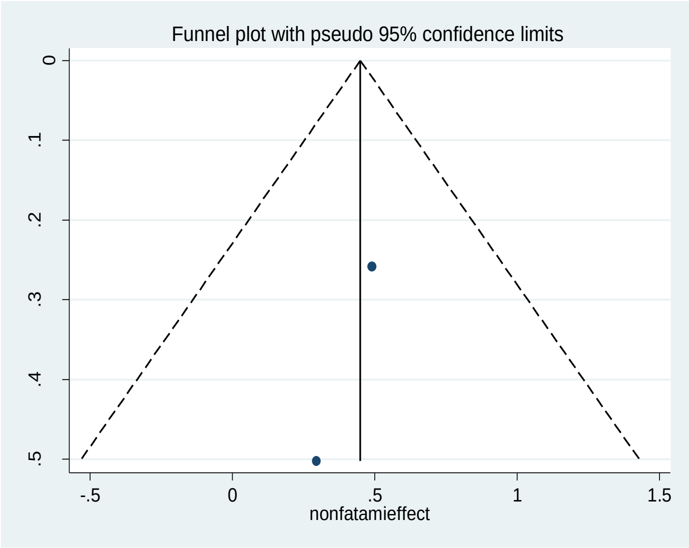

Figure S76: **Funnel plot of effect of the Mediterranean diet on non-fatal myocardial infarction**

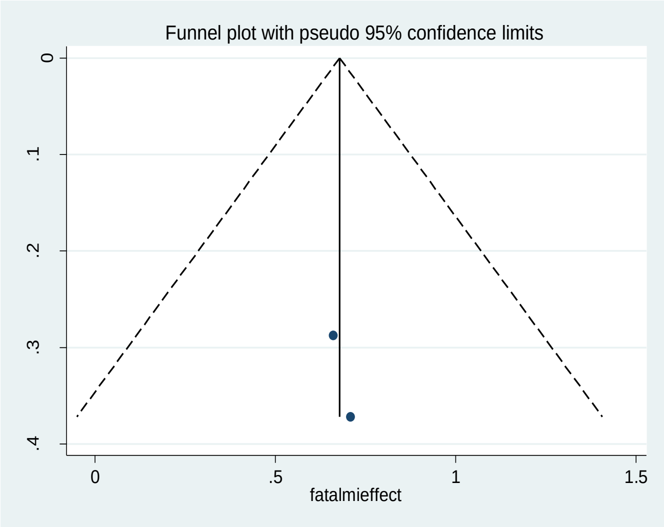

Figure S77: **Funnel plot of effect of the Mediterranean diet on fatal myocardial infarction**

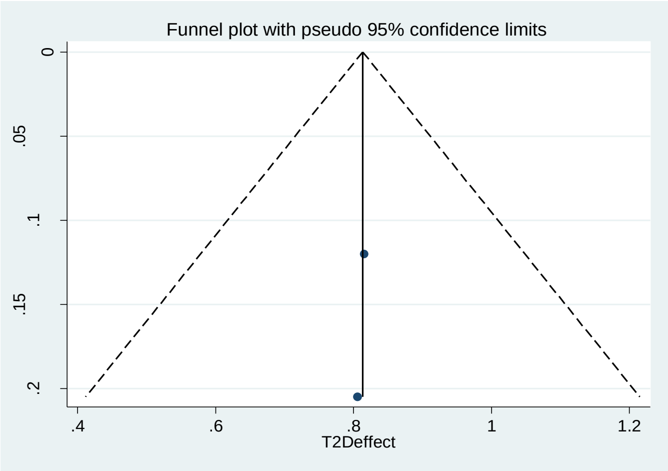

Figure S78: **Funnel plot of effect of the Mediterranean diet on type 2 diabetes incidence**

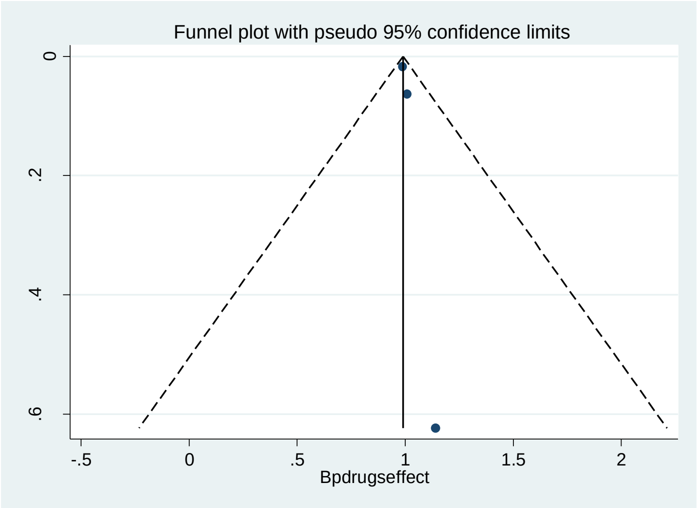

Egger's test P value= 0.211

Figure S79: **Funnel plot of effect of the Mediterranean diet on the use of blood pressure lowering drugs**

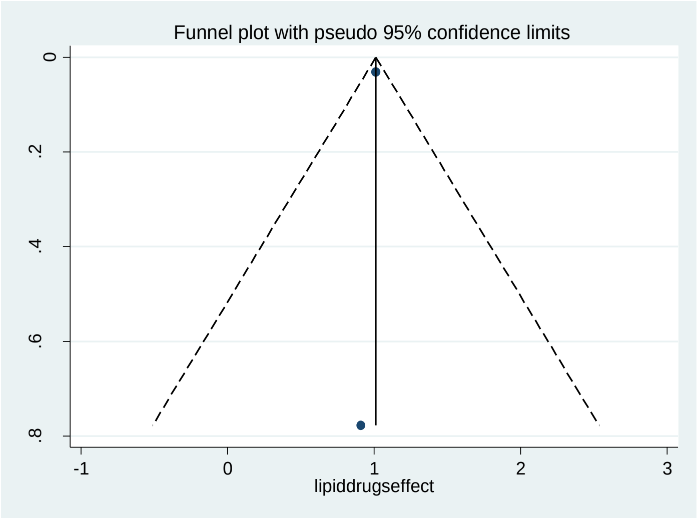

Figure S80: **Funnel plot of effect of the Mediterranean diet on the use of lipid-lowering agents**

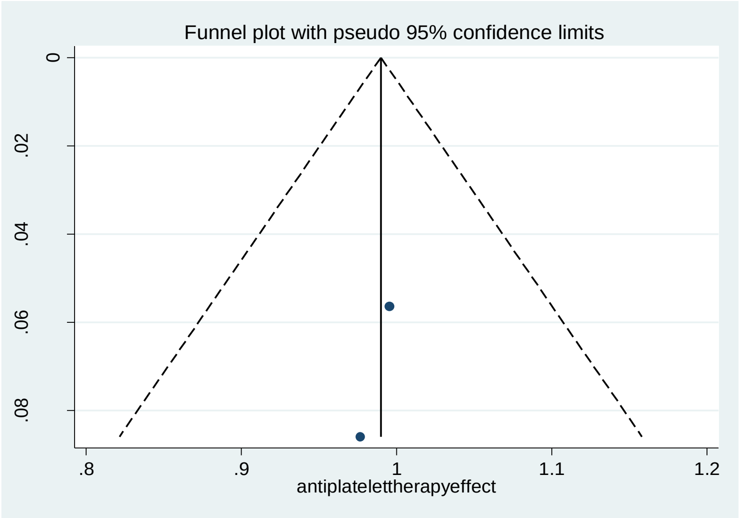

Figure S81: **Funnel plot of effect of the Mediterranean diet on the use of anti-platelet therapy**

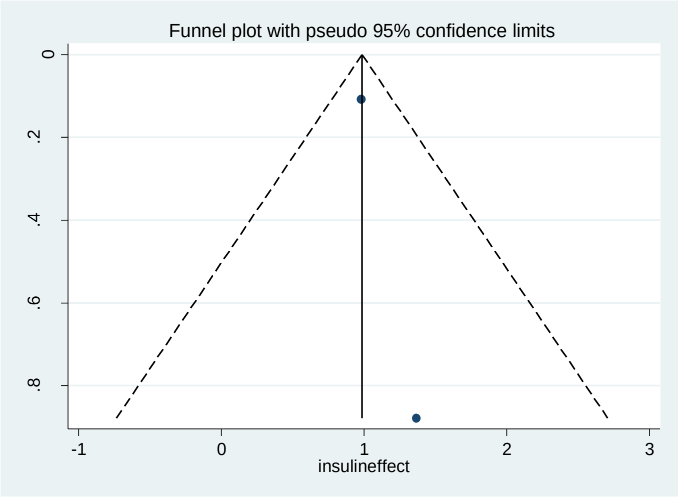

Figure S82: **Funnel plot of effect of the Mediterranean diet on the use of insulin**

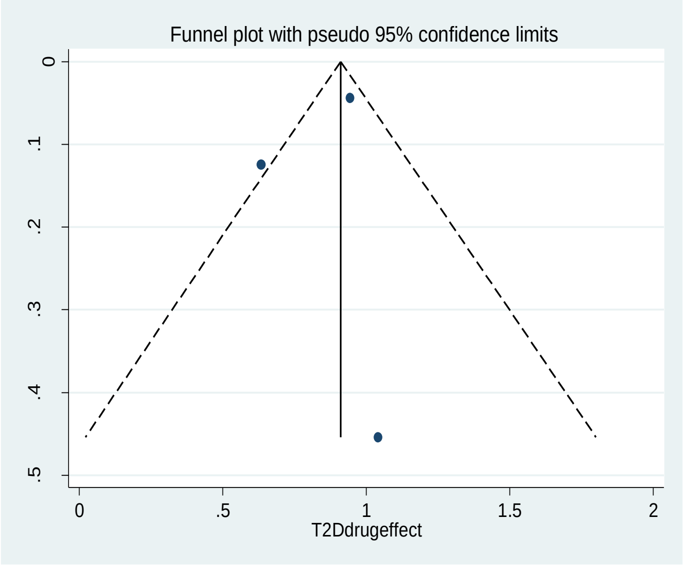

Figure S83: **Funnel plot of effect of the Mediterranean diet on the use of oral antidiabetic agents**

Supplementary Table S12. Detailed risk of bias for each included paper reporting a non-randomised controlled trial

	**Confounding**	**Selection for participants**	**Classification of interventions**	**Deviation from intended intervention**	**Missing data**	**Measurement of outcome**	**Selection of the reported result**	**Overall**
Biolato et al 2019 [35]	Serious	No information	Low	Low	Low	Low	Moderate	Serious
Papadaki & Scott 2005, 2008 [80, 81]	Low	Low	Low	Low	Low	Low	Moderate	No information
Richard et al 2011, 2013 [85, 86]	Low	Unclear	Low	Low	Low	Low	Moderate	No information
Rogerson et al 2018 [87]	Serious	Critical	Low	Low	Low	Low	Moderate	Critical
Thomazella et al 2011 [101]	Serious	Low	Low	No information	Low	Low	Moderate	Serious
Timar et al 2013 [102]	No information	No information	Low	No information	No information	Low	Moderate	No information

Supplementary Table S13. Effect of the Mediterranean diet on anthropometric, blood pressure, biochemical, insulin resistance, oxidative stress, inflammatory and endothelial function markers related to the metabolic syndrome, according to different subgroups*

Outcome and/or subgroup	Studies	Effect estimate (MD, 95% CI)	***P***	I^2	***P* across subgroups**	***P* for heterogeneity**	**% of between-study variance explained**
**Anthropometric markers**							
Body weight (kg)	40	-1·72 (-2·40, -1·05)	<0·001	98·6%			
**Food supplementation**							
Supplementation of foods	20	-0·41 (-0·98, 0·17)	0·167	83·7%			
No food supplementation	20	-2·82 (-3·75, -1·90)	<0·001	98·9%	0.011	<0.001	17.9%
**Location**							
Mediterranean	18	-2.29 (-3.04, -1.53)	<0.001	96.0%			
Non-Mediterranean	22	-1.16 (-2.28, -0.04)	0.043	98.8%	0.185	<0.001	2.6%
**Health status at baseline**							
Healthy	14	-2.10 (-3.36, -0.85)	0.001	93.8%			
Unhealthy	26	-1.55 (-2.39, -0.71)	<0.001	99.0%	0.466	<0.001	3.2%
**Intervention duration**							
<6 months	19	-1.01 (-2.23, 0.22)	0.109	93.3%			
≥6 months	21	-2.33 (-3.23, -1.43)	<0.001	99.2%	0.145	<0.001	3.4%
**Sample size**							
<150 participants	26	-1.70 (-2.89, -0.51)	0.005	92.1%			

≥150 participants	14	-1.82 (-2.83, -0.80)	<0.001	99.5%	0.911	<0.001	-3.3%
**Type of intervention**							
MD alone	27	-1.09 (-1.97, -0.21)	0.016	99.0%			
MD with other dietary component	13	-2.97 (-4.19, -1.75)	<0.001	95.2%	0.043	<0.001	7.7%
**Type of control treatment**							
No treatment	7	-2.18 (-3.23, -1.14)	<0.001	92.2%			
Low-fat diet	9	0.01 (-1.10, 1.12)	0.985	92.0%			
Reduced energy, low-fat diet	5	-4.07 (-7.15, -0.99)	0.010	95.8%			
Low-fat, high-carbohydrate diet	2	1.50 (-1.86, 4.86)	0.383	0.0%			
Healthy diet or dietary guidelines	5	-4.38 (-6.37, -2.40)	<0.001	92.9%			
NCEP diet	3	-3.22 (-6.06, -0.38)	0.026	99.8%	0.443	<0.001	-1.0%
Body mass index (kg/m^2)	37	-0·41 (-0·71, -0·10)	0·010	98·6%			
**Food supplementation**							
Supplementation of foods	16	0·11 (-0·24, 0·46)	0·531	96·2%			
No food supplementation	21	-0·70 (-0·94, -0·46)	<0·001	88·6%	<0.001	<0.001	55.3%
**Location**							
Mediterranean	16	-0.43 (-0.66, -0.19)	<0.001	90.5%			
Non-Mediterranean	21	-0.27 (-0.71, 0.17)	0.226	96.2%	0.298	<0.001	-2.8%
**Health status at baseline**							
Healthy	11	-0.62 (-1.11, -0.12)	0.014	90.3%			

Unhealthy	26	-0.30 (-0.66, 0.06)	0.107	98.9%	0.314	<0.001	-5.0%
**Intervention duration**							
<6 months	16	-0.17 (-0.65, 0.31)	0.481	95.6%			
≥6 months	21	-0.45 (-0.65, -0.25)	<0.001	88.6%	0.163	<0.001	4.8%
**Sample size**							
<150 participants	23	-0.35 (-0.73, 0.02)	0.067	97.2%			
≥150 participants	14	-0.43 (-0.69, -0.18)	0.001	92.9%	0.569	<0.001	1.4%
**Type of intervention**							
MD alone	24	-0.19 (-0.54, 0.16)	0.290	97.1%			
MD with other dietary component	13	-0.63 (-0.92, -0.34)	<0.001	90.1%	0.035	<0.001	8.0%
**Type of control treatment**							
No treatment	5	-0.42 (-0.91, 0.08)	0.098	92.9%			
Low-fat diet	8	-0.03 (-0.52, 0.46)	0.901	92.1%			
Reduced energy, low-fat diet	5	-0.72 (-1.30, -0.14)	0.015	71.4%			
Healthy diet or dietary guidelines	9	-0.65 (-1.06, -0.23)	0.002	92.6%			
NCEP diet	2	-0.69 (-1.60, 0.21)	0.134	57.6%	0.255	<0.001	6.1%
Waist circumference (cm)	27	-1.47 (-2.54, -0.39)	0.007	99.6%			
**Food supplementation**							
Supplementation of foods	11	-0.37 (-1.55, 0.80)	0.532	93.2%			
No food supplementation	16	-1.84 (-2.53, -1.14)	<0.001	92.4%	0.191	<0.001	5.3%

**Location**							
Mediterranean	14	-1.54 (-2.21, -0.86)	<0.001	93.6%			
Non-Mediterranean	13	-0.93 (-2.28, 0.42)	0.179	90.3%	0.554	<0.001	-3.5%
**Health status at baseline**							
Healthy	7	-2.53 (-4.63, -0.44)	0.018	85.1%			
Unhealthy	20	-1.11 (-2.35, 0.13)	0.079	99.7%	0.380	<0.001	-1.0%
**Intervention duration**							
<6 months	13	-1.42 (-3.11, 0.28)	0.102	94.8%			
≥6 months	14	-1.30 (-1.87, -0.73)	<0.001	89.8%	0.915	<0.001	-6.1%
**Sample size**							
<150 participants	19	-1.56 (-2.71, -0.41)	0.008	97.6%			
≥150 participants	8	-1.10 (-1.81, -0.40)	0.002	90.4%	0.693	<0.001	-6.1%
**Type of intervention**							
MD alone	11	-0.87 (-2.08, 0.35)	0.162	96.8%			
MD with other dietary component	16	-1.66 (-2.52, -0.80)	<0.001	94.8%	0.295	<0.001	-1.9%
**Type of control treatment**							
No treatment	2	-3.04 (-4.95, -1.13)	0.002	0.0%			
Low-fat diet	7	0.38 (-1.24, 1.99)	0.649	98.3%			
Reduced energy, low-fat diet	4	-5.67 (-9.49, -1.86)	0.004	95.4%			
Healthy diet or dietary guidelines	6	-1.64 (-2.60, -0.68)	0.001	94.8%	0.984	<0.001	-7.0%

**Blood pressure**							
Systolic blood pressure (mm Hg)	27	-1·34 (-2·00, -0·67)	<0·001	93·6%			
**Food supplementation**							
Supplementation of foods	13	-1·44 (-2·78, -0·11)	0·034	91·1%			
No food supplementation	14	-1·53 (-2·41, -0·65)	<0·001	92·2%	0.702	<0.001	-8.5%
**Location**							
Mediterranean	10	-1.19 (-2.17, -0.21)	0.017	94.4%			
Non-Mediterranean	17	-1.66 (-3.08, -0.25)	0.021	91.5%	0.472	<0.001	-6.7%
**Health status at baseline**							
Healthy	8	-1.43 (-2.52, -0.34)	0.010	0.0%			
Unhealthy	19	-1.28 (-2.02, -0.54)	<0.001	95.4%	0.806	0.043	-5.9%
**Intervention duration**							
<6 months	12	-0.62 (-2.58, 1.34)	0.534	87.4%			
≥6 months	15	-1.91 (-2.54, -1.29)	<0.001	92.3%	0.287	<0.001	3.0%
**Sample size**							
<150 participants	16	-1.01 (-2.73, 0.71)	0.250	87.4%			
≥150 participants	11	-1.50 (-2.36, -0.63)	0.001	96.5%	0.608	<0.001	-6.9%
**Type of intervention**							
MD alone	16	-1.55 (-2.45, -0.65)	0.001	94.3%			
MD with other dietary component	11	-0.87 (-2.12, 0.38)	0.172	92.5%	0.299	<0.001	-6.1%

**Type of control treatment**							
No treatment	4	-1.98 (-2.20, -1.76)	<0.001	0.0%			
Low-fat diet	6	-1.67 (-3.49, 0.15)	0.072	54.1%			
Reduced energy, low-fat diet	4	1.25 (-1.17 (3.68)	0.312	94.9%			
Healthy diet or dietary guidelines	5	-2.22 (-3.78, -0.66)	0.005	95.4%			
NCEP diet	2	-3.47 (-5.13, -1.81)	<0.001	46.6%	0.246	<0.001	4.0%
Diastolic blood pressure (mm Hg)	27	-0.81 (-1.30, -0.32)	0.001	92.8%			
**Food supplementation**							
Supplementation of foods	13	-0.42 (-1.41, 0.56)	0.399	90.6%			
No food supplementation	14	-1.43 (-1.87, -1.00)	<0.001	79.7%	0.165	<0.001	17.0%
**Location**							
Mediterranean	10	-0.87 (-1.67, -0.08)	0.030	93.6%			
Non-Mediterranean	17	-0.79 (-1.57, -0.00)	0.050	87.9%	0.915	<0.001	-8.0%
**Health status at baseline**							
Healthy	8	-1.01 (-2.19, 0.16)	0.091	48.7%			
Unhealthy	19	-0.77 (-1.31, -0.22)	0.006	94.7%	0.728	0.058	-6.6%
**Intervention duration**							
<6 months	12	-0.78 (-2.17, 0.62)	0.275	86.9%			
≥6 months	15	-1.12 (-1.57 (-0.67)	<0.001	90.7%	0.599	<0.001	-0.2%
**Sample size**							

<150 participants	16	-0.91 (-1.74, -0.07)	0.034	71.1%			
≥150 participants	11	-0.77 (-1.43, -0.12)	0.021	96.4%	0.784	<0.001	-8.3%
**Type of intervention**							
MD alone	16	-0.58 (-1.34, 0.19)	0.140	95.2%			
MD with other dietary component	11	-1.28 (-1.82, -0.74)	<0.001	72.7%	0.419	<0.001	1.9%
**Type of control treatment**							
No treatment	4	-0.56 (-1.76, 0.65)	0.365	56.2%			
Low-fat diet	6	-0.38 (-1.14, 0.38)	0.328	20.2%			
Reduced energy, low-fat diet	4	-0.15 (-2.54, 2.24)	0.904	96.1%			
Healthy diet or dietary guidelines	5	-1.36 (-2.08, -0.64)	<0.001	86.3%			
NCEP diet	2	-1.82 (-2.57, -1.07)	<0.001	35.6%	0.485	<0.001	1.5%
Glucose (mg/dL)	31	-2.98 (-4.54, -1.42)	<0.001	98.1%			
**Food supplementation**							
Supplementation of foods	16	-0.20 (-2.31, 1.91)	0.854	94.5%			
No food supplementation	15	-5.81 (-8.00, -3.63)	<0.001	98.5%	0.016	<0.001	18.9%
**Location**							
Mediterranean	17	-5.47 (-8.21, -2.72)	<0.001	98.5%			
Non-Mediterranean	14	-0.31 (-2.65, 2.03)	0.883	97.0%	0.025	<0.001	13.3%
**Health status at baseline**							
Healthy	10	-1.67 (-3.85, 0.51)	0.132	94.6%			

Unhealthy	21	-3.77 (-5.87, -1.68)	<0.001	98.4%	0.311	<0.001	-1.0%
**Intervention duration**							
<6 months	17	-0.18 (-1.78, 1.42)	0.824	94.6%			
≥6 months	14	-6.97 (-9.47, -4.46)	<0.001	98.1%	0.002	<0.001	27.0%
**Sample size**							
<150 participants	21	-1.72 (-4.56, 1.12)	0.235	98.3%			
≥150 participants	10	-5.15 (-7.25, -3.05)	<0.001	97.5%	0.103	<0.001	3.0%
**Type of intervention**							
MD alone	18	-2.45 (-5.10, 0.20)	0.070	98.7%			
MD with other dietary component	13	-3.67 (-5.45, -1.90)	<0.001	95.%	0.647	<0.001	-2.7%
**Type of control treatment**							
No treatment	2	3.16 (-6.33, 12.65)	0.514	91.1%			
Low-fat diet	5	1.69 (-1.06, 4.44)	0.229	77.5%			
Reduced energy, low-fat diet	5	-4.70 (-8.50, -0.91)	0.015	96.7%			
Low-fat, high-carbohydrate diet	2	0.72 (-3.33, 4.77)	0.726	0.0%			
Healthy diet or dietary guidelines	7	-7.83 (-13.13, -2.54)	0.004	98.9%			
NCEP diet	2	-0.95 (-8.59, 6.70)	0.809	98.8%	0.293	<0.001	1.0%
Insulin (μU/mL)	20	-0.94 (-1.72, -0.16)	0.019	97.2%			
**Food supplementation**							
Supplementation of foods	10	-0.31 (-1.70, 1.07)	0.657	94.5%			

No food supplementation	10	-1.54 (-2.64, -0.44)	0.006	98.1%	0.390	<0.001	-0.5%
**Location**							
Mediterranean	13	-1.55 (-2.43, -0.67)	0.001	97.5%			
Non-Mediterranean	7	0.27 (-1.95, 2.50)	0.809	95.6%	0.205	<0.001	4.6%
**Health status at baseline**							
Healthy	7	-0.24 (-0.67, 0.19)	0.273	58.3%			
Unhealthy	13	-1.26 (-2.40, -0.11)	0.031	97.7%	0.424	0.025	-3.5%
**Intervention duration**							
<6 months	12	-0.54 (-1.51, 0.42)	0.270	95.1%			
≥6 months	8	-1.49 (-2.80, -0.19)	0.025	97.9%	0.512	<0.001	-3.1%
**Sample size**							
<150 participants	12	-0.85 (-2.32, 0.62)	0.259	97.8%			
≥150 participants	8	-1.06 (-1.97, -0.15)	0.022	95.9%	0.959	<0.001	-6.9%
**Type of intervention**							
MD alone	11	-0.17 (-1.59, 1.25)	0.811	93.9%			
MD with other dietary component	9	-1.64 (-2.73, -0.56)	0.003	98.3%	0.253	<0.001	2.1%
**Type of control treatment**							
No treatment	2	2.03 (-3.74, 7.80)	0.491	79.2%			
Low-fat diet	4	0.89 (-2.08, 3.86)	0.558	96.8%			
Reduced energy, low-fat diet	3	-0.47 (-0.73, -0.21)	<0.001	61.3%			

Low-fat, high-carbohydrate diet	2	-4.74 (-9.53, 0.04)	0.052	81.2%			
Healthy diet or dietary guidelines	5	-3.37 (-3.76, -2.99)	<0.001	34.0%	0.179	<0.001	10.0%
HOMA-IR index	18	-0·42 (-0·70, -0·15)	0·003	97·7%			
**Food supplementation**							
Supplementation of foods	7	-0.45 (-0.77, -0.12)	0·007	86·5%			
No food supplementation	11	-0·39 (-0·73, -0·06)	0·022	97·6%	0.806	<0.001	-7.0%
**Location**							
Mediterranean	14	-0·38 (-0·69, -0·06)	0.019	98.1%			
Non-Mediterranean	4	-0.58 (-1.21, 0.05)	0.071	88.5%	0.590	<0.001	-5.1%
**Health status at baseline**							
Healthy	4	-0.26 (-0.57, 0.05)	0.096	84.6%			
Unhealthy	14	-0.49 (-0.83, -0.14)	0.006	98.0%	0.460	<0.001	-4.3%
**Intervention duration**							
<6 months	9	-0.23 (-0.62, 0.16)	0.247	93.3%			
≥6 months	9	-0.61 (-0.89, -0.34)	<0.001	96.1%	0.243	<0.001	7.4%
**Sample size**							
<150 participants	9	-0.36 (-0.86, 0.15)	0.164	96.0%			
≥150 participants	9	-0.49 (-0.87, -0.10)	0.013	98.4%	0.671	<0.001	-5.0%
**Type of intervention**							
MD alone	8	0.55 (-1.04, -0.05)	0.030	98.5%			

MD with other dietary component	10	-0.32 (-0.65, 0.02)	0.064	95.6%	0.471	<0.001	-2.1%
**Type of control treatment**							
Low-fat diet	4	-0.75 (-1.09, -0.41)	<0.001	66.1%			
Reduced energy, low-fat diet	5	-0.32 (-0.59, -0.04)	0.023	90.6%			
Healthy diet or dietary guidelines	5	-0.33 (-1.20, 0.54)	0.460	97.7%	0.389	<0.001	-1.6%
Total cholesterol (mg/dL)	37	-5·70 (-9·96, -1·43)	0·009	98·6%			
**Food supplementation**							
Supplementation of foods	19	-6·79 (-13·35, -0·23)	0·042	95·2%			
No food supplementation	18	-4·52 (-10·65, 1·60)	0·148	99·1%	0.616	<0.001	-2.2%
**Location**							
Mediterranean	16	-6.38 (-9.71, -3.06)	<0.001	93.6%			
Non-Mediterranean	21	-5.18 (-13.18, 2.83)	0.255	97.5%	0.700	<0.001	-2.5%
**Health status at baseline**							
Healthy	11	-1.74 (-14.63, 11.15)	0.791	98.6%			
Unhealthy	26	-7.29 (-11.66, -2.92)	0.001	98.2%	0.285	<0.001	0.0%
**Intervention duration**							
<6 months	18	-5.59 (-12.02, 0.85)	0.089	96.7%			
≥6 months	19	-5.75 (-11.36, -0.15)	0.044	98.6%	0.969	<0.001	-3.0%
**Sample size**							
<150 participants	24	-5.13 (-11.5, 1.24)	0.114	97.2%			

≥150 participants	13	-6.71 (-12.60, -0.82)	0.025	98.6%	0.756	<0.001	-2.7%
**Type of intervention**							
MD alone	23	-5.62 (-12.02, 0.77)	0.085	98.1%			
MD with other dietary component	14	-5.79 (-9.57, -2.01)	0.003	93.3%	0.985	<0.001	-3.0%
**Type of control treatment**							
No treatment	5	-3.68 (-7.96, 0.60)	0.092	28.4%			
Low-fat diet	7	-10.37 (-14.75, -5.60)	<0.001	0.0%			
Reduced energy, low-fat diet	4	-4.63 (-6.77, -2.49)	<0.001	41.8%			
Healthy diet or dietary guidelines	8	-3.22 (-10.70, 4.27)	0.400	97.8%			
NCEP diet	2	-2.18 (-37.56, 33.19)	0.904	99.3%	0.995	<0.001	-3.9%
LDL-cholesterol (mg/dL)	29	-8·24 (-13·50, -2·99)	0·002	99·6%			
**Food supplementation**							
Supplementation of foods	16	-9·01 (-15·16, -2·86)	0·004	98·5%			
No food supplementation	13	-7·31 (-14·50, -0·12)	0·046	99·1%	0.727	<0.001	-3.9%
**Location**							
Mediterranean	11	-7.94 (-14.58, -1.30)	0.019	99.1%			
Non-Mediterranean	18	-8.50 (-14.05, -2.96)	0.003	97.3%	0.915	<0.001	-3.9%
**Health status at baseline**							
Healthy	9	-8.61 (-21.49, 4.27)	0.190	98.7%			
Unhealthy	20	-8.03 (-14.36, -1.70)	0.013	99.7%	0.890	<0.001	-3.9%

**Intervention duration**							
<6 months	15	-7.48 (-14.70, -0.26)	0.042	98.3%			
≥6 months	14	-9.10 (-17.33, -0.88)	0.030	99.8%	0.724	<0.001	-3.4%
**Sample size**							
<150 participants	21	-7.95 (-14.14, -1.77)	0.012	97.6%			
≥150 participants	8	-9.00 (-19.20, 1.20)	0.084	99.9%	0.871	<0.001	-4.1%
**Type of intervention**							
MD alone	20	-10.03 (-16.53, -3.53)	0.003	99.7%			
MD with other dietary component	9	-4.44 (-12.68, 3.81)	0.291	98.2%	0.267	<0.001	0.6%
**Type of control treatment**							
No treatment	3	-1.35 (-8.38, 5.68)	0.707	83.7%			
Low-fat diet	8	-7.42 (-11.23, -3.62)	<0.001	56.0%			
Reduced energy, low-fat diet	2	-0.54 (-22.68, 21.61)	0.962	97.8%			
Healthy diet or dietary guidelines	6	-11.35 (-18.88, -3.81)	0.003	97.0%			
NCEP diet	2	-2.05 (-35.28, 31.17)	0.904	99.4%	0.402	<0.001	-0.2%
HDL-cholesterol (mg/dL)	36	1·30 (0·38, 2·21)	0·005	98·1%			
**Food supplementation**							
Supplementation of foods	19	0·96 (-0·24, 2·15)	0·116	88·7%			
No food supplementation	17	1·66 (0·51, 2·80)	0·004	96·3%	0.556	<0.001	-2.5%
**Location**							

Mediterranean	15	0.88 (-0.84, 2.61)	0.227	98.4%			
Non-Mediterranean	21	1.68 (0.75, 2.61)	0.002	77.0%	0.620	<0.001	-2.7%
**Health status at baseline**							
Healthy	11	1.55 (-1.91, 5.00)	0.380	97.6%			
Unhealthy	25	1.27 (0.24, 2.30)	0.015	98.3%	0.898	<0.001	-3.5%
**Intervention duration**							
<6 months	18	0.31 (-1.34, 1.95)	0.714	92.1%			
≥6 months	18	2.34 (1.05, 3.62)	<0.001	98.9%	0.081	<0.001	8.3%
**Sample size**							
<150 participants	24	1.29 (-0.40, 2.99)	0.135	95.3%			
≥150 participants	12	1.38 (-0.09, 2.86)	0.067	99.2%	0.916	<0.001	-3.5%
**Type of intervention**							
MD alone	23	1.15 (-0.07, 2.38)	0.065	98.4%			
MD with other dietary component	13	1.55 (-0.26, 3.36)	0.094	97.1%	0.753	<0.001	-3.4%
**Type of control treatment**							
No treatment	4	1.06 (-1.75, 3.87)	0.459	74.0%			
Low-fat diet	8	0.26 (-0.54, 1.05)	0.526	0.0%			
Reduced energy, low-fat diet	4	1.57 (-0.13, 3.27)	0.070	84.1%			
Healthy diet or dietary guidelines	8	1.48 (-1.52, 4.47)	0.334	98.2%			
NCEP diet	2	2.40 (2.35, 2.45)	<0.001	0.0%	0.898	<0.001	-4.0%

Triglycerides (mg/dL)	38	-12.30 (-15.60, -8.99)	<0.001	94.8%			
**Food supplementation**							
Supplementation of foods	19	-8.89 (-15.81, -1.97)	0.012	92.8%			
No food supplementation	19	-15.87 (-19.15, -12.59)	<0.001	91.7%	0.167	<0.001	5.4%
**Location**							
Mediterranean	16	-14.19 (-18.58, -9.80)	<0.001	93.1%			
Non-Mediterranean	22	-10.88 (-17.30, -4.46)	0.002	92.4%	0.527	<0.001	-1.4%
**Health status at baseline**							
Healthy	10	-10.80 (-15.20, -6.39)	<0.001	70.3%			
Unhealthy	28	-12.81 (-16.71, -8.91)	<0.001	95.1%	0.816	<0.001	-3.9%
**Intervention duration**							
<6 months	18	-8.75 (-14.72, -2.79)	0.004	91.1%			
≥6 months	20	-15.90 (-18.70, -13.09)	<0.001	87.1%	0.164	<0.001	4.1%
**Sample size**							
<150 participants	25	-11.20 (-15.99, -6.42)	<0.001	88.5%			
≥150 participants	13	-14.75 (-19.65, -9.86)	<0.001	96.2%	0.611	<0.001	-1.8%
**Type of intervention**							
MD alone	23	-21.91 (-22.13, -21.69)	<0.001	95.9%			
MD with other dietary component	15	-16.11 (-17.20, -15.03)	<0.001	82.9%	0.569	<0.001	-1.9%
**Type of control treatment**							

No treatment	5	-18.01 (-25.16, -10.86)	<0.001	65.1%			
Low-fat diet	8	-7.17 (-18.33, 3.99)	0.208	70.5%			
Reduced energy, low-fat diet	4	-14.86 (-28.86, -0.87)	0.037	96.5%			
Healthy diet or dietary guidelines	9	-13.71 (-18.26, -9.16)	<0.001	88.6%			
NCEP diet	2	-18.58 (-28.85, -8.32)	<0.001	64.6%	0.781	<0.001	-3.8%
**Inflammatory markers**							
C-reactive protein (mg/L)	13	-0·77 (-1·14, -0·39)	<0·001	92·6%			
**Food supplementation**							
Supplementation of foods	10	-0·95 (-1·78, -0·12)	0·024	94·0%			
No food supplementation	3	-0·69 (-0·88, -0·49)	<0·001	73·6%	0.610	<0.001	-15.0%
**Location**							
Mediterranean	4	-1.08 (-1.57, -0.58)	<0.001	95.5%			
Non-Mediterranean	9	-0.67 (-1.37, 0.03)	0.061	89.2%	0.761	<0.001	-10.8%
**Health status at baseline**							
Healthy	3	-0.63 (-0.85, -0.41)	<0.001	16.3%			
Unhealthy	10	-0.76 (-1.38, -0.13)	0.017	94.3%	0.818	<0.001	-16.0%
**Intervention duration**							
<6 months	7	-0.87 (-1.69, -0.05)	0.038	91.8%			
≥6 months	6	-0.90 (-1.35, -0.45)	<0.001	92.8%	0.745	<0.001	-19.3%
**Sample size**							

<150 participants	10	-0.63 (-1.14, -0.12)	0.016	90.8%			
≥150 participants	3	-1.03 (-2.79, 0.73)	0.253	96.9%	0.727	<0.001	-9.8%
**Type of intervention**							
MD alone	9	-1.09 (-1.99, -0.19)	0.018	94.6%			
MD with other dietary component	4	-0.65 (-0.87, -0.43)	<0.001	72.4%	0.377	0.012	-7.5%
**Type of control treatment**							
No treatment	2	-3.66 (-6.21, -1.12)	0.005	54.6%			
Low-fat diet	5	-0.51 (-2.08, 1.06)	0.522	96.1%			
Reduced energy, low-fat diet	2	-0.18 (-1.30, 0.95)	0.760	70.3%	0.700	<0.001	-18.2%

CI, confidence intervals; HDL, high density lipoprotein cholesterol; HOMA-IR, homeostatic model assessment of insulin resistance; LDL, low density lipoprotein cholesterol; MD, mean difference; NCEP, National Cholesterol Education programme.

[*]Findings are based on random-effects meta-analysis (inverse variance) and meta-regressions. I^2 represents the magnitude of heterogeneity. Subgroup analyses were conducted, with studies stratified according to food supplementation, location, health status at baseline, intervention duration, sample size, type of intervention and type of control treatment. For reference, the first line for each outcome presents the findings before the subgroup analysis took place. Subgroup analyses were only conducted for outcomes with at least 10 studies included.

Supplementary Table S14. Effect of the Mediterranean diet on anthropometric, blood pressure, biochemical, insulin resistance, oxidative stress, inflammatory and endothelial function markers related to the metabolic syndrome (sensitivity analysis, following the exclusion of non-randomised controlled trials, cross-over trials and trials with ≥1,000 participants)*

Outcome and/or subgroup	Studies	Participants	Effect estimate (MD, 95% CI)	*P*-value	I^2
**Anthropometric markers**					
Body weight (kg)	40	12571	-1.72 (-2.40, -1.04)	<0.001	99.0%
After exclusion of non-randomised trials	36	12421	-1.69 (-2.40, -0.98)	<0.001	99.0%
After exclusion of cross-over trials	33	12251	-1.84 (-2.57, -1.11)	<0.001	99.0%
After exclusion of both the above	30	12105	-2.04 (-2.80, -1.29)	<0.001	99.0%
After exclusion of studies with ≥1000 participants	38	4124	-1.75 (-2.72, -0.78)	<0.001	98.6%
Body mass index (kg/m^2)	37	5679	-0.41 (-0.71, -0.10)	0.010	99.0%
After exclusion of non-randomised trials	32	5373	-0.43 (-0.76, -0.10)	0.010	99.0%
After exclusion of cross-over trials	31	5381	-0.42 (-0.61, -0.23)	<0.001	89.0%
After exclusion of both the above	28	5150	-0.49 (-0.85, -0.14)	0.007	99.0%
After exclusion of studies with ≥1000 participants	36	4679	-0.38 (-0.69, -0.07)	0.015	98.6%
Waist circumference (cm)	27	9690	-1.47 (-2.54, -0.39)	0.007	100.0%
After exclusion of non-randomised trials	24	9564	-0.94 (-2.08, 0.19)	0.100	100.0%
After exclusion of cross-over trials	23	9525	-1.24 (-1.78, -0.70)	<0.001	90.0%
After exclusion of both the above	21	9433	-1.13 (-1.69, -0.58)	<0.001	90.0%
After exclusion of studies with ≥1000 participants	26	2243	-1.52 (-2.62, -0.41)	0.007	99.6%
Total fat mass (kg)	9	963	-0.47 (-1.53, 0.60)	0.390	85.0%
After exclusion of non-randomised trials	9	963	-0.47 (-1.53, 0.60)	0.390	85.0%
After exclusion of cross-over trials	6	716	-1.38 (-2.97, 0.21)	0.090	89.0%
After exclusion of both the above	6	716	-1.38 (-2.97, 0.21)	0.090	89.0%
After exclusion of studies with ≥1000 participants	9	963	-0.47 (-1.53, 0.60)	0.390	85.0%
Total body fat (%)	8	661	-0.12 (-1.60, 1.37)	0.880	90.0%
After exclusion of non-randomised trials	8	661	-0.12 (-1.60, 1.37)	0.880	90.0%
After exclusion of cross-over trials	6	554	-0.88 (-2.49,0.72)	0.280	87.0%
After exclusion of both the above	6	554	-0.88 (-2.49,0.72)	0.280	87.0%
After exclusion of studies with ≥1000 participants	8	661	-0.12 (-1.60, 1.37)	0.880	90.0%
**Blood pressure**					
Systolic blood pressure (mm Hg)	27	4930	-1.33 (-2.00, -0.67)	<0.001	94.0%
After exclusion of non-randomised trials	22	4624	-1.94 (-2.55, -1.33)	<0.001	91.0%

After exclusion of cross-over trials	22	4717	-1.27 (-1.97, -0.58)	<0.001	94.0%
After exclusion of both the above	18	4445	-1.34 (-2.02, -0.66)	<0.001	94.0%
After exclusion of studies with ≥1000 participants	25	2802	-1.10 (-1.89, -0.31)	0.006	91.3%
Diastolic blood pressure (mm Hg)	27	4930	-0.81 (-1.30, -0.32)	0.001	93.0%
After exclusion of non-randomised trials	22	4624	-0.94 (-1.48, -0.41)	<0.001	93.0%
After exclusion of cross-over trials	22	4717	-0.65 (-1.17, -0.13)	0.010	94.0%
After exclusion of both the above	18	4445	-0.75 (-1.32, -0.19)	0.009	94.0%
After exclusion of studies with ≥1000 participants	25	2802	-0.70 (-1.24, -0.15)	0.013	88.1%
**Biochemical markers**					
Glucose (mg/dL)	31	3662	-2.98 (-4.54, -1.42)	<0.001	98.0%
After exclusion of non-randomised trials	28	3536	-3.05 (-4.69, -1.40)	<0.001	98.0%
After exclusion of cross-over trials	24	3313	-4.07 (-5.87, -2.27)	<0.001	98.0%
After exclusion of both the above	22	3221	-4.21 (-6.09, -2.33)	<0.001	98.0%
After exclusion of studies with ≥1000 participants	30	2662	-2.91 (-4.89, -0.94)	0.004	98.0%
Insulin (μU/mL)	20	2184	-0.94 (-1.72, -0.16)	0.020	97.0%
After exclusion of non-randomised trials	20	2184	-0.94 (-1.72, -0.16)	0.020	97.0%
After exclusion of cross-over trials	15	1913	-0.77 (-1.71, 0.17)	0.110	97.0%
After exclusion of both the above	15	1913	-0.77 (-1.71, 0.17)	0.110	97.0%
After exclusion of studies with ≥1000 participants	20	2184	-0.94 (-1.72, -0.16)	0.020	97.0%
HOMA-IR index	17	2098	-0.44 (-0.72, -0.15)	0.003	98.0%
After exclusion of non-randomised trials	16	2064	-0.41 (-0.70, -0.12)	0.006	98.0%
After exclusion of cross-over trials	14	1999	-0.35 (-0.66, -0.04)	0.030	98.0%
After exclusion of both the above	14	1999	-0.35 (-0.66, -0.04)	0.030	98.0%
After exclusion of studies with ≥1000 participants	17	2098	-0.44 (-0.72, -0.15)	0.003	98.0%
HbA1c (%)	6	869	-0.16, (-0.37, 0.05)	0.140	78.0%
After exclusion of non-randomised trials	5	713	-0.29, (-0.40, -0.18)	<0.001	4.0%
After exclusion of cross-over trials	6	869	-0.16, (-0.37, 0.05)	0.140	78.0%
After exclusion of both the above	5	713	-0.29, (-0.40, -0.18)	<0.001	4.0%
After exclusion of studies with ≥1000 participants	6	869	-0.16, (-0.37, 0.05)	0.140	78.0%
Total cholesterol (mg/dL)	37	4603	-5.70 (-9.96, -1.43)	0.009	99.0%
After exclusion of non-randomised trials	32	4391	-8.86 (-13.07, -4.65)	<0.001	98.0%
After exclusion of cross-over trials	32	4221	-5.11 (-9.36, -0.86)	0.020	98.0%
After exclusion of both the above	28	4043	-8.43 (-12.41, -4.45)	<0.001	98.0%
After exclusion of studies with ≥1000 participants	36	3603	-5.22 (-8.93, -1.52)	0.006	95.9%
LDL-cholesterol (mg/dL)	29	3633	-8.24 (-13.50, -2.99)	0.002	100.0%
After exclusion of non-randomised trials	24	3289	-9.71 (-15.54, -3.87)	0.001	100.0%
After exclusion of cross-over trials	24	3330	-9.01 (-14.74, -3.29)	0.002	100.0%

After exclusion of both the above	20	3020	-9.93 (-16.30, -3.56)	0.002	100.0%
After exclusion of studies with ≥1000 participants	28	2633	-7.84 (-12.17, -3.5)	<0.001	98.3%
HDL-cholesterol (mg/dL)	36	4433	1.30 (0.38, 2.21)	0.005	98.0%
After exclusion of non-randomised trials	31	4221	0.93 (-0.06, 1.92)	0.060	98.0%
After exclusion of cross-over trials	30	4106	1.58 (0.56, 2.61)	0.002	98.0%
After exclusion of both the above	26	3928	1.16 (0.06, 2.26)	0.040	99.0%
After exclusion of studies with ≥1000 participants	35	3433	1.28 (0.12, 2.44)	0.031	96.8%
Triglycerides (mg/dL)	38	4658	-12.30 (-15.60, -8.99)	<0.001	95.0%
After exclusion of non-randomised trials	33	4314	-13.32 (-16.71, -9.93)	<0.001	94.0%
After exclusion of cross-over trials	32	4331	-11.56 (-15.23, -7.88)	<0.001	95.0%
After exclusion of both the above	28	4021	-13.47 (-17.17, -9.76)	<0.001	95.0%
After exclusion of studies with ≥1000 participants	37	3658	-11.86 (-15.44, -8.28)	<0.001	90.9%
Non-HDL-cholesterol (mg/dL)	2	584	-0.06 (-0.59, 0.47)	0.840	60.0%
After exclusion of non-randomised trials	2	584	-0.06 (-0.59, 0.47)	0.840	60.0%
After exclusion of cross-over trials	2	584	-0.06 (-0.59, 0.47)	0.840	60.0%
After exclusion of both the above	2	584	-0.06 (-0.59, 0.47)	0.840	60.0%
After exclusion of studies with ≥1000 participants	2	584	-0.06 (-0.59, 0.47)	0.840	60.0%
Total:HDL-cholesterol ratio	6	670	-0.83 (-2.67, 1.01)	0.380	100.0%
After exclusion of non-randomised trials	5	646	-1.03 (-3.06, 1.01)	0.320	100.0%
After exclusion of cross-over trials	5	629	-0.21 (-0.53, 0.11)	0.200	70.0%
After exclusion of both the above	4	605	-0.25 (-0.61, 0.10)	0.160	77.0%
After exclusion of studies with ≥1000 participants	6	670	-0.83 (-2.67, 1.01)	0.380	100.0%
Homocysteine (μmol/L)	2	171	-0.04 (-0.61, 0.53)	0.880	0.0%
After exclusion of non-randomised trials	2	171	-0.04 (-0.61, 0.53)	0.880	0.0%
After exclusion of cross-over trials	2	171	-0.04 (-0.61, 0.53)	0.880	0.0%
After exclusion of both the above	2	171	-0.04 (-0.61, 0.53)	0.880	0.0%
After exclusion of studies with ≥1000 participants	2	171	-0.04 (-0.61, 0.53)	0.880	0.0%
AST (UI/L)	3	193	-3.44 (-7.55, 0.68)	0.100	98.0%
After exclusion of non-randomised trials	2	159	-1.22 (-5.43, 2.99)	0.570	98.0%
After exclusion of cross-over trials	1	41	-3.40 (-4.45, -2.35)	<0.001	-
After exclusion of both the above	1	41	-3.40 (-4.45, -2.35)	<0.001	-
After exclusion of studies with ≥1000 participants	3	193	-3.44 (-7.55, 0.68)	0.100	98.0%
ALT (UI/L)	8	729	-5.66 (-9.44, -1.87)	0.003	97.0%
After exclusion of non-randomised trials	7	695	-2.39 (-5.77, 0.99)	0.170	96.0%
After exclusion of cross-over trials	5	553	-3.72 (-6.89, -0.55)	0.020	80.0%
After exclusion of both the above	5	553	-3.72 (-6.89, -0.55)	0.020	80.0%
After exclusion of studies with ≥1000 participants	8	729	-5.66 (-9.44, -1.87)	0.003	97.0%

GGT (UI/L)	7	393	-2.51 (-5.38, 0.35)	0.090	64.0%
After exclusion of non-randomised trials	6	359	-2.51 (-5.46, 0.44)	0.090	70.0%
After exclusion of cross-over trials	4	217	-2.98 (-7.94, 1.98)	0.240	71.0%
After exclusion of both the above	4	217	-2.98 (-7.94, 1.98)	0.240	71.0%
After exclusion of studies with ≥1000 participants	7	393	-2.51 (-5.38, 0.35)	0.090	64.0%
Hepatic fat mass (%)	3	224	-2.80 (-5.52, -0.08)	0.040	79.0%
After exclusion of non-randomised trials	3	224	-2.80 (-5.52, -0.08)	0.040	79.0%
After exclusion of cross-over trials	2	200	-2.04 (-5.95, 1.88)	0.310	86.0%
After exclusion of both the above	2	200	-2.04 (-5.95, 1.88)	0.310	86.0%
After exclusion of studies with ≥1000 participants	3	224	-2.80 (-5.52, -0.08)	0.040	79.0%
**Oxidative stress markers**					
Oxidised LDL-cholesterol (U/L)	2	970	4.38 (-16.49, 25.25)	0.680	98.0%
After exclusion of non-randomised trials	1	930	-6.30 (-11.08, -1.52)	0.010	-
After exclusion of cross-over trials	2	970	4.38 (-16.49, 25.25)	0.680	98.0%
After exclusion of both the above	1	930	-6.30 (-11.08, -1.52)	0.010	-
After exclusion of studies with ≥1000 participants	2	970	4.38 (-16.49, 25.25)	0.680	98.0%
**Inflammatory markers**					
C-reactive protein (mg/L)	13	1071	-0.77 (-1.14, -0.39)	<0.001	93.0%
After exclusion of non-randomised trials	12	1031	-0.70 (-1.10, -0.30)	<0.001	93.0%
After exclusion of cross-over trials	11	964	-0.99 (-1.35, -0.63)	<0.001	89.0%
After exclusion of both the above	10	924	-0.94 (-1.33, -0.55)	<0.001	89.0%
After exclusion of studies with ≥1000 participants	13	1071	-0.77 (-1.14, -0.39)	<0.001	93.0%
Interleukin-6 (pg/mL)	4	261	-0.61 (-0.93, -0.29)	<0.001	0.0%
After exclusion of non-randomised trials	4	261	-0.61 (-0.93, -0.29)	<0.001	0.0%
After exclusion of cross-over trials	4	261	-0.61 (-0.93, -0.29)	<0.001	0.0%
After exclusion of both the above	4	261	-0.61 (-0.93, -0.29)	<0.001	0.0%
After exclusion of studies with ≥1000 participants	4	261	-0.61 (-0.93, -0.29)	<0.001	0.0%
Adiponectin (µg/mL)	4	546	0.76 (-1.16, 2.67)	0.440	70.0%
After exclusion of non-randomised trials	4	546	0.76 (-1.16, 2.67)	0.440	70.0%
After exclusion of cross-over trials	4	546	0.76 (-1.16, 2.67)	0.440	70.0%
After exclusion of both the above	4	546	0.76 (-1.16, 2.67)	0.440	70.0%
After exclusion of studies with ≥1000 participants	4	546	0.76 (-1.16, 2.67)	0.440	70.0%
Tumor necrosis factor-a (pg/mL)	2	283	-0.81 (-1.03, -0.60)	<0.001	0.0%
After exclusion of non-randomised trials	2	283	-0.81 (-1.03, -0.60)	<0.001	0.0%
After exclusion of cross-over trials	1	165	-1.20 (-2.33, -0.07)	0.040	-
After exclusion of both the above	1	165	-1.20 (-2.33, -0.07)	0.040	-
After exclusion of studies with ≥1000 participants	2	283	-0.81 (-1.03, -0.60)	<0.001	0.0%

**Markers of endothelial function**					
Flow-mediated dilatation (%)	3	206	1·49 (0·61, 2·37)	<0·001	0·0%
After exclusion of non-randomised trials	3	206	1·49 (0·61, 2·37)	<0·001	0·0%
After exclusion of cross-over trials	3	206	1·49 (0·61, 2·37)	<0.001	0·0%
After exclusion of both the above	3	206	1·49 (0·61, 2·37)	<0.001	0·0%
After exclusion of studies with ≥1000 participants	3	206	1·49 (0·61, 2·37)	<0·001	0·0%

ALT, alanine transaminase; AST, aspartame transaminase; CI, confidence intervals; GGT, gamma glutamyl transferase; HDL, high density lipoprotein cholesterol; HbA1c, glycosylated haemoglobin; HOMA-IR, homeostatic model assessment of insulin resistance; LDL, low density lipoprotein cholesterol; MD, mean difference.

* Findings are based on random-effects meta-analysis (inverse variance). I^2 represents the magnitude of heterogeneity. For reference, the first line for each outcome presents the findings before the sensitivity analysis took place.
